# Deoxygenation of Epoxides with Carbon Monoxide†

**DOI:** 10.1002/chem.202002651

**Published:** 2020-07-23

**Authors:** Theo Maulbetsch, Eva Jürgens, Doris Kunz

**Affiliations:** ^1^ Institut für Anorganische Chemie University of Tübingen Auf der Morgenstelle 18 72076 Tübingen Germany

**Keywords:** deoxygenation, epoxides, homogeneous catalysis, iridium, pincer ligands

## Abstract

The use of carbon monoxide as a direct reducing agent for the deoxygenation of terminal and internal epoxides to the respective olefins is presented. This reaction is homogeneously catalyzed by a carbonyl pincer‐iridium(I) complex in combination with a Lewis acid co‐catalyst to achieve a pre‐activation of the epoxide substrate, as well as the elimination of CO_2_ from a γ‐2‐iridabutyrolactone intermediate. Especially terminal alkyl epoxides react smoothly and without significant isomerization to the internal olefins under CO atmosphere in benzene or toluene at 80–120 °C. Detailed investigations reveal a substrate‐dependent change in the mechanism for the epoxide C−O bond activation between an oxidative addition under retention of the configuration and an S_N_2 reaction that leads to an inversion of the configuration.

## Graphical abstract

**Deoxygenate it**! Carbon monoxide is the direct and environmentally benign deoxygenation agent for epoxides. This reaction is catalyzed by an iridium pincer complex and co‐catalyst LiNTf_2_ via an unprecedented γ‐2‐iridabutyrolactone intermediate. LiNTf_2_ is not only necessary for the epoxide activation, but also for the decarboxylation of the iridalactone (see scheme).

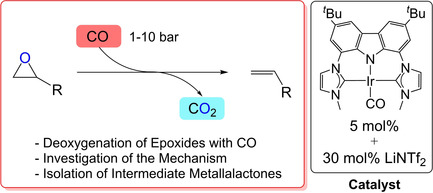

## Introduction

**Scheme 1:**
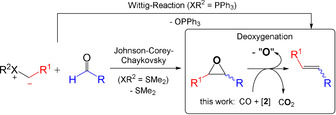
Deoxygenation of epoxides as part of a two‐step Wittig reaction.

Apart from the water‐gas shift reaction itself as well as reductions using hydrogen produced by this reaction in situ,^1^ the use of carbon monoxide as a direct deoxygenation agent is very rare in homogenous catalysis and hard to distinguish from the former one depending on the system.^1^, ^2^, ^3^, ^4^ The deoxygenation of epoxides (Scheme 1) to alkenes is an important reaction in organic chemistry and some non‐catalytic as well as catalytic reactions (homogeneous and heterogeneous) are known. One early report on the deoxygenation of epoxides was in 1955 by Wittig and Haag,^5^ who used triphenylphosphine as a deoxygenation reagent at 180 °C to deoxygenate α,β‐epoxy esters that were obtained from the Darzens reaction. They recognized that the temperature can be reduced when adding hydroquinone. Vedejs and Fuchs developed this reaction further by reacting *cis* or *trans* epoxides to the betaines with lithium diphenylphosphide and methyl iodide, which subsequently led to formation of the olefin with inversion of the configuration at room temperature.^6^, ^7^ Later, rhenium and molybdenum catalyzed variations were developed.^8^, ^9^ In these cases, the stoichiometric amounts of triorganophosphine oxide as a side product is problematic to get recycled back to the phosphine or phosphide. Other stoichiometric or over‐stoichiometric deoxygenation reagents for epoxides^10^, ^11^ are Co_2_(CO)_8_,^12^ Fe(CO)_5_,^13^ SmI_2_,^14^ In/InCl,^15^ lithium amide bases with silylboranes^16^ or diazomalonate.^17^ The latter two are catalyzed by copper‐nanoparticles and copper complexes, respectively. Not yet fully understood is the deoxygenation of arene oxides and their oxepine tautomers by [Rh(CO)_2_Cl]_2_ or [Rh(C_2_H_4_)_2_Cl]_2_ in CHCl_3_.^18^, ^19^ Environmentally much more benign are variations—in addition to electrochemical methods^20^—in which hydrogen is used as a deoxygenation agent to obtain water as a byproduct. Methylrheniumtrioxide (Re(CH_3_)O_3_) is a suitable catalyst for this reaction that is carried out at 150 °C. However, the hydrogenation of the product olefins can be an unwanted side reaction.^21^ Silver and gold nanoparticles are heterogeneous catalysts in a process that uses hydrogen directly^22^ or generates it in situ from alcohols.^23^ The currently most efficient system uses reactive hydrogen species that are produced in an in situ water gas shift reaction from carbon monoxide by a hydrotalcite‐supported gold nanoparticle catalyst.^24^, ^25^ However, above 50 °C the formation of free hydrogen is observed, which increases the CO consumption and requires additional security measures. While aryl oxiranes already react at room temperature, alkyl oxiranes require temperatures of 110 °C and an organic solvent.

**Figure 1:**
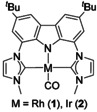
Structure of the pincer complexes **1** and **2** bearing the bimca ligand.

In the following, we will report on the efficient and stereoselective deoxygenation of terminal and internal alkyl oxiranes as well as aryl oxiranes with carbon monoxide as a direct deoxygenation agent catalyzed by the nucleophilic carbonyl pincer‐complexes **1** 26 and **2** 27 (Figure 1). Using CO directly is advantageous as the formation of hydrogen, which can cause further side reactions, is avoided *per se*. In combination with the Johnson–Corey–Chaykovsky reaction of aldehydes with sulfur ylides to epoxides, this sequence equals to the Wittig reaction, but with formation of CO_2_ instead of triphenylphosphine oxide (Scheme 1).

## Results and Discussion

**Scheme 2:**
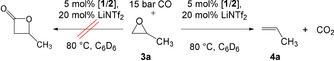
Deoxygenation of epoxides (right) with CO catalyzed by the electron rich pincer complexes **1** and **2**. The formation of β‐lactones was not observed.

In 2015 we reported on the nucleophilic isomerization of terminal epoxides to methyl ketones using the electron rich carbonyl pincer‐rhodium catalyst **1** in combination with LiNTf_2_ as a Lewis acid co‐catalyst.^28^ As one of the observed side reactions was a CO insertion into the ring‐opened intermediate, we envisaged the possibility of a catalytic reaction to obtain β‐lactones, as it is known for Co_2_(CO)_8_.^29^, ^30^ However, we were surprised, when we found propylene as the only organic product when reacting propylene oxide, 5 mol % of **1** and 20 mol % of LiNTf_2_ under 15 bar of CO in C_6_D_6_ at 80 °C (Scheme 2). The formation of CO_2_ was recognized in the ^13^C NMR spectrum.

This prompted us to investigate this unusual reactivity further.^31^ While the rhodium catalyst **1** seemed to be unstable at the elevated temperature, we were pleased to find that the analogous iridium complex **2** was not only more stable, but also considerably more active (Table 1, entry 5). Other (commercially available) rhodium, iridium, cobalt or iron complexes were much less active under the identical conditions (entries 2–4 and 7–9). With the pincer complex [Ir(bimca^C5^)CO], in which the carbene moieties are connected via a 1,5‐pentadiyl chain (bimca^C5^), mainly the epoxide isomerization product, methyl butyl ketone, was detected (entry 6).

**Table 1:** Catalyst screening for the catalytic deoxygenation of 1,2‐epoxyhexane with CO.^[a]^

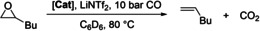				
Entry	Catalyst	*t* [h]	Yield (alkene) [%]^[b]^	Side product^[b]^
1	[Rh(bimca^Me^)CO] (**1**)	24	50	–
2	[RhCl(PPh_3_)_3_]	24	0	–
3	[Ir(CO)Cl(PPh_3_)_2_]	24	0	–
4	[Ir(acac)(CO)_2_]	24 90	31 68	–
5	[Ir(bimca^Me^)CO] (**2**)	24	98	2 % int. olefin
6	[Ir(bimca^C5^)(CO)]	24	8	87 % methyl butyl ketone
7	[Co(Cp)(CO)_2_]	24	0	–
8	[Co_2_(CO)_8_]	24	0	–
9	[Fe(CO)_5_]	24	0	–

[a] Reaction was carried out in a pressure NMR tube (Wilmad) at 0.2 m epoxide concentration. [b] From ^1^H NMR calibrated to 1,3,5‐trimethoxybenzene as internal standard.

Therefore, we optimized the reaction conditions for catalyst **2** (Table 2). Without catalyst or without co‐catalyst, the reaction did not proceed (entries 1 and 2). THF as a solvent is not favorable, presumably as it reduces the Lewis acidity of the LiNTf_2_ co‐catalyst (entry 7). Afterwards we probed the pressure dependence of carbon monoxide. The reaction rate is roughly pressure‐independent (entries 8–10). However, at low pressure, the amount of CO (1.2 equivalents) comes close to the required stoichiometric amounts in our setup (Wilmad Pressure NMR tubes) and thus the insufficient amount of CO dissolved in benzene explains the lower yields. At 15 bar the yield seems to decrease slightly (entry 10). Therefore, we chose 10 bar as an optimal pressure in all further experiments. To shorten the reaction time, we also increased the temperature and found full conversion at 100 °C after 8 h and at 120 °C after 2 h (entry 11–12). Even at room temperature some product formed, but at a very slow reaction rate (entry 13). With 1‐hexene oxide, we recognized a slow isomerization of 1‐hexene to internal hexenes. The degree of isomerization increases, when the reaction mixture is kept at the reaction conditions after full conversion of the epoxide. This isomerization also requires the presence of the Lewis acid as a co‐catalyst as it was confirmed by independent measurements (Supporting Information).

**Table 2:** Optimizing the reaction conditions for the catalytic deoxygenation of 1,2‐epoxyhexane with CO.^[a]^

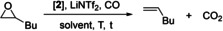									
Entry	[**2**] [mol %]	LiNTf_2_ [mol %]	CO [bar]	Solvent	*T* [°C]	*t* [h]	Conv.^[b]^ [%]	Yield^[b]^ [%]	Isomer^[b]^ [%]
1	5	–	10	C_6_D_6_	80	24	0	0	
2	–	30	10	C_6_D_6_	80	24	0	0	
3	1	6.0	10	C_6_D_6_	80	24	32	32	
4	2.5	15	10	C_6_D_6_	80	24	81	70	
5	5	30	10	C_6_D_6_	80	24	100	97	2
6	5	30	10	[D_8_]Tol	80	24	92	91	
7	5	30	10	[D_8_]THF	80	24	7	3	–
8	5	30	2.0	C_6_D_6_	80	24	83	79	4
9	5	30	5.9	C_6_D_6_	80	24	94	90	4
10	5	30	15	C_6_D_6_	80	24	94	91	3
11	5	30	10	[D_8_]Tol	100	8	100	93	
12	5	30	10	[D_8_]Tol	120	0.5 2	96 100	92 82	
13	5	30	10	C_6_D_6_	rt	168	6	6

[a] Reaction was carried out in a pressure NMR tube (Wilmad) at 0.2 m epoxide concentration. [b] From ^1^H NMR calibrated to 1,3,5‐trimethoxybenzene as internal standard.

**Figure 2:**
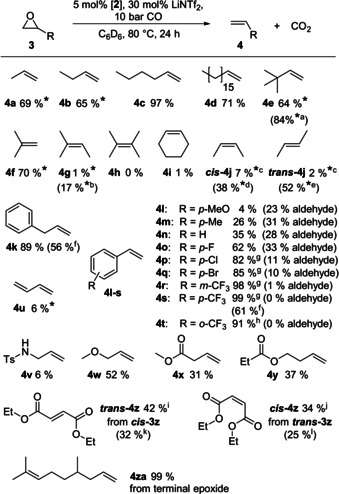
Chemo‐ and stereoselective deoxygenation of various epoxides. Reactions were carried out in a medium‐wall NMR tube with pressure valve (Wilmad) at 0.2 m epoxide concentration. Yields were determined by ^1^H NMR spectroscopy calibrated to 1,3,5‐trimethoxybenzene as internal standard after release of the overpressure. * indicates the ^1^H NMR yield of the dissolved amount of the gaseous reaction product, measured under CO pressure. [a] 120 °C, 24 h; [b] 120 °C, 96 h; [c] 224 h; [d] 120 °C, 72 h, 11 % ***trans*** **‐4 j**; [e] 120 °C, 72 h, 2 % ***cis*** **‐4 j**; [f] isolated yield, 1 mmol scale, 1 bar CO; 72 h; [g] 100 °C, 24 h; [h] 100 °C, 48 h; [i] 240 h, 9 % ***cis*** **‐3 z**; [j] 240 h; 8 % ***trans*** **‐3 z**; [k] 120 °C, 48 h, 7 % ***cis*** **‐3 z**; [l] 120 °C, 48 h, 16 % ***trans*** **‐3 z**.

We screened a broad range of substrates and found that terminal epoxides react most readily (Figure 2). The reaction under the optimized conditions works very well for aliphatic, terminal epoxides (**3 a**–**3 e**) including benzyl epoxide (**3 k**) (no isomerization to methyl styrene is observed), and 1,1‐disubstituted epoxides (**3 f**), whereas internal epoxides (**3 g**–**3 j**) are much harder to deoxygenate as well as terminal epoxides bearing functional groups (**3 l**–**3 y**). In case of styrene oxide (**3 n**) the formation of 28 % of benzyl aldehyde indicates a Lewis acid catalyzed epoxide isomerization as side reaction. The use of LiBr (solubilized with 4 equiv of tetrahydrofuran) instead of LiNTf_2_, which circumvented this reaction in the nucleophilic epoxide isomerization,^32^ did not have any beneficial effect. Although the amount of the aldehyde by‐product could be reduced pronouncedly using LiBr or LiI, the reaction did not go to completion due to deactivation of the catalyst by formation of [Ir(bimca)(CO)X_2_] (X=Br, I) (see Supporting Information). While electron rich styrene oxides (**3 l**, **3 m**) lead to an increase of side reactions, electron poor styrene oxides **3 o–3 t** react smoothly at 100 °C and good to excellent yields were obtained with few or negligible formation of the aldehyde. Moreover, also the sterically hindered *o*‐CF_3_ substituted styrene oxide **3 t** can be deoxygenated to **4 t** in very high yield, albeit at a lower reaction rate. Most striking is the stereochemistry when 1,2‐disubstituted epoxides are reacted: deoxygenation of *cis* epoxide ***cis*** **‐3 j** led to full retention of the configuration in olefin ***cis*** **‐4 j**. Slow subsequent isomerization of the olefin into the more stable *trans*‐butene, however, can occur towards the end of the reaction. Respectively, *trans*‐butene oxide (***trans*** **‐3 j**) is converted into *trans*‐butene (***trans*** **‐4 j**), albeit at a slower reaction rate. The opposite selectivity, inversion of the configuration, is observed with the doubly ester functionalized substrates ***cis*** **‐3 z** and ***trans*** **‐3 z**. Although the reaction is extremely slow at 80 °C in benzene (10 d) it proceeds with moderate yields and low isomerization. At 120 °C the epoxy succinate ***cis*** **‐3 z** reacted to diethyl fumarate (***trans*** **‐4 z**) in 32 % yield and the *trans* epoxide ***trans*** **‐3 z** to diethyl maleate (***cis*** **‐4 z**) in 25 % already after 24 h and with still good selectivity. The selectivity of the deoxygenation can be explained with a substrate‐dependent change in the epoxide activation mechanism (vide infra) from oxidative addition in the case of alkyl epoxides to an S_N_2 mechanism for the C−O bond activation in the case of ethyl carboxylates (**3 z**; see below).

To demonstrate the applicability of our reaction to natural product derived substrates, we obtained **4 za** in 99 % yield from deoxygenation of **3 za**, which can be easily obtained from citronellal in a Johnson‐Corey‐Chaykovsky reaction with dimethylsulfonium ylide (Scheme 1). For practical reasons, we also found that the reaction can be run at 1 bar of CO on a preparative scale, to avoid the necessity of using special autoclaves. On a 1 mmol scale (at cost of a longer reaction time of 72 h) we isolated the products **4 k** (56 %) and **4 s** (61 %) by transfer of the volatiles in vacuo and subsequent removal of benzene (except of 0.5 equiv remaining). Product loss (24 % (**4 k**) and 17 % (**4 s**)) was found in the benzene fraction as the scale was too small for a fractional distillation.

### Mechanistic investigations

First, we tested whether a second CO ligand coordinates to **2** to form an 18 VE complex **2+CO**. However, under 10 bar of CO no change of the ^1^H NMR signals of **2** was observed. Moreover, in the ^13^C NMR spectrum we see two separate CO signals, one for the iridium complex **2** and one for free CO. This confirms that no fast exchange of the CO ligand on the NMR timescale occurs. In the IR spectrum (toluene) at atmospheric pressure of CO, neither a shift nor an additional band was observed, and under CO atmosphere only complex **2** crystallized out. Therefore, we conclude that the 16 VE complex **2** is the catalytic active species. Also for rhodium complex **1** we had not observed any exchange of the CO ligand by ^13^CO under 3 bar, neither under UV light nor by heating at 60–70 °C.^26^

**Scheme 3:**
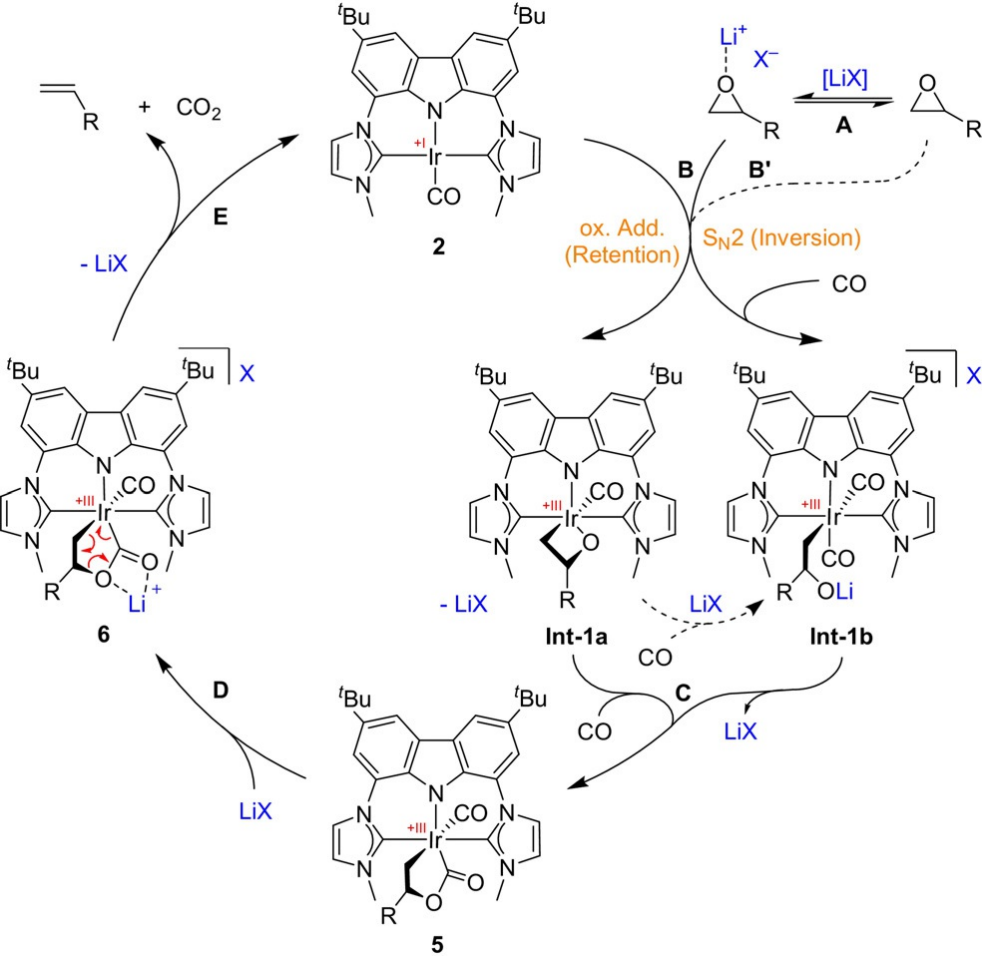
Proposed mechanism of the deoxygenation of epoxides with CO catalyzed by iridium complex **2**. The Lewis acid co‐catalyst is necessary to activate the epoxide (**A**) as well as to induce the decarboxylation (**D**, **E**). The involvement of **Int‐1 a** depends on the substrate: 1,2‐dialkyl epoxides react to **Int‐1 a** in an oxidative addition of the epoxide C−O bond, whereas *cis*‐ and *trans*‐1,2‐diethylcarboxyl epoxide get activated through an S_N_2 mechanism.

The first step of the catalytic cycle (Scheme 3) is the cleavage of the C−O bond of the epoxide (**B**). This step requires pre‐activation of the epoxide by the Lewis acid (step **A**), as it is already known from the nucleophilic epoxide isomerization with rhodium catalyst **1** and its congeners.^28^, ^32^ Although the complete catalytic cycle does not operate in absence of the Lewis acid (Table 2, entry 1), the C−O bond cleavage of propylene oxide still occurs at 80 °C, albeit extremely slowly (**B’**). We took advantage of this observation and reacted **2** with propylene oxide (**3 a**) and 10 bar of CO at 80 °C for 10 days, and were able to isolate intermediate **5 a**. Due to the presence of the CO atmosphere, further reaction of **Int‐1** to acetone or a hydridoalkyl complex^33^ is blocked. The identity of **5 a**, which contains a hitherto unprecedented 2‐irida‐γ‐lactone moiety, was proven by spectroscopic methods. The chiral center of the metallalactone moiety reduces the symmetry of the complex so that 8 signals for the aromatic H atoms and two for the *N*‐methyl groups are observed. The two signals of the diastereomeric methylene protons of the lactone ring are detected at 1.71 and 1.80 ppm and the methine signal at 3.92 ppm. From the ^13^C NMR spectrum further evidence for the formation of the CO complex **5 a** was obtained (172.6 (IrCO_2_R), 182.8 (CO), 78.8 (O‐*C*H(CH_3_)‐), and 27.6 (Ir‐CH_2_) ppm). The IR spectrum (ATR) confirms this with characteristic bands at 2014 (CO) and 1630 cm^−1^ (IrCO_2_R). The latter value is similar to the one of a 2‐platina‐γ‐lactone (1644 cm^−1^ (NaCl)), the only structurally characterized 2‐metalla‐γ‐lactone in literature,^34^ and to an acyclic iridaester (1638 (benzene) and 1650 cm^−1^ (KBr)).^35^ When monitoring the catalytic reaction NMR‐spectroscopically (with Lewis acid) at 60 °C over a period of 23 h, this intermediate can also be detected. Its concentration reaches quickly a maximum of 5 mol % and slowly declines during the course of the reaction (see Supporting Information). This shows that the epoxide activation step **A** and the decarboxylation step **E** have about roughly the same reaction rate under the applied conditions for substrate **3 a**.

**Figure 3:**
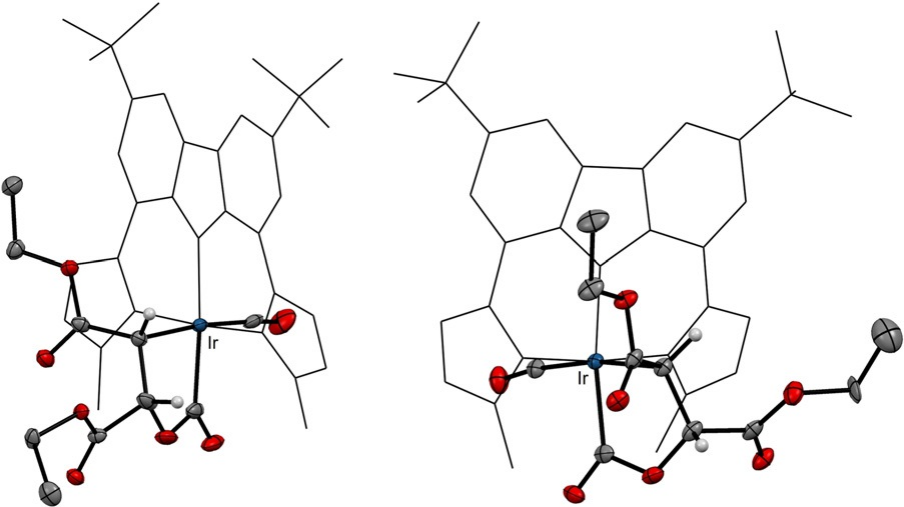
Molecular structure of the isolated intermediates ***cis*** **‐5 z** from (***trans*** **‐3 z**) (left) and ***trans*** **‐5 z** (from ***cis*** **‐3 z**) (right) from reactions of **2** and **3 z** without added Lewis acid. Atoms are shown with anisotropic atomic displacement parameters at the 50 % probability level for the lactone moiety and the rest in wire‐frame style for clarity reasons. Hydrogen atoms (except for the iridacycle) as well as co‐crystallized benzene molecules are omitted for clarity.

The reaction of *cis*‐ and *trans*‐1,2‐dialkyl‐substituted epoxide (***cis*** **‐3 j**, ***trans*** **‐3 j**) led to retention of the configuration in the respective products (***cis*** **‐4 j** and ***trans*** **‐4 j**) and to inversion in the case of 1,2‐diethylcarboxylate‐substituted epoxides (**3 z**). As an epoxide opening that follows an S_N_2 mechanism would lead to inversion of the configuration in a possible intermediate **Int‐1 b**, we conclude that the activation step **B** proceeds via oxidative addition under C−O bond cleavage to the iridaoxetane^10^, ^11^, ^36^ **Int‐1 a** with the substrates ***cis*** **‐3 j** and ***trans*** **‐3 j**. This is followed by CO induced migration of the alkoxide from Ir to a CO ligand^35^, ^37^, ^38^ to form **5 j**. Under the applied conditions, **Int‐1 b** could also be formed from **Int‐1 a** by Lewis acid ring opening followed by CO coordination and lactone formation by nucleophilic addition of the alkoxide to CO^39^, ^40^ to form **5 j** without change of the configuration. Attempts to detect **5 j** from 1,2‐dialkylsubstituted epoxides **3 j** failed, as these substrates do not react without presence of the Lewis acid. During the catalytic conditions, no intermediate was observed which counts for the oxidative addition **B** to be the rate‐determining step with these substrates. In case of the ester functionalized epoxides **3 z** the intermediate **5 z** was obtained much easier without Lewis acid compared to propylene oxide (**3 a**) as a substrate. The ^3^ *J*_HH_ coupling between the methine protons of the metallalactone moiety of ***trans*** **‐5 z** (obtained from ***cis*** **‐3 z**) of 6.8 Hz and that of ***cis*** **‐5 z** (obtained from ***trans*** **‐3 z**) of 3.2 Hz, confirm the inversion of the configuration. Proof for the configuration as well as for the formation of the 2‐irida‐γ‐lactone was obtained from the X‐ray crystal structure analyses (Figure 3). To our knowledge, this is the second example of a structurally characterized 2‐metalla‐γ‐lactone.

An inversion of the configuration was also observed by Dowd and Kang, using stoichiometric amounts (referring to CO) of Co_2_(CO)_8_ in the reaction with **3 z**.^12^ They suggested an analogous intermediate. Their reaction occurred already at room temperature, albeit in neat epoxide. S_N_2 reactions with Rh^I^ and Ir^I^ complexes are typically observed in the oxidative addition of alkyl iodide in the Monsanto or Cativa acetic acid process,^41^ while metallaoxetane formation from oxiranes with Rh^I^ and Ir^I^ was investigated by Milstein and co‐workers.^33^, ^42^ In our case, it seems energetically favored for iridium complex **2** to open the electron poor epoxide **3 z** in an S_N_2 fashion and the more electron rich internal epoxide **3 j** by oxidative addition of the C−O bond.

The accumulation of intermediate **5** in absence of a Lewis acid also means that the subsequent decarboxylation step **E** requires the presence of a Lewis acid as well. To confirm this, we heated intermediate **5 a** in C_6_D_6_ (without CO atmosphere) up to 80 °C and found no CO_2_ elimination, while addition of the Lewis acid led to slow formation of the signals of propene (**4 a**) already at room temperature along with the signals of **2**. When stoichiometric amounts of LiNTf_2_ were added, we observed a slight shift in the ^1^H NMR signals, which is most pronounced for the *N*‐CH_3_ and the adjacent imidazole signals (see Supporting Information Figure S1). Therefore, we propose formation of Lewis acid adduct **6** (step **D**) to be mandatory for the CO_2_ elimination (step **E**). In the case of the isolated intermediates ***cis*** **‐5 z** and ***trans*** **‐5 z** the CO_2_ elimination step required even heating to 60 °C and thus is the rate limiting step using these substrates.

**Scheme 4:**
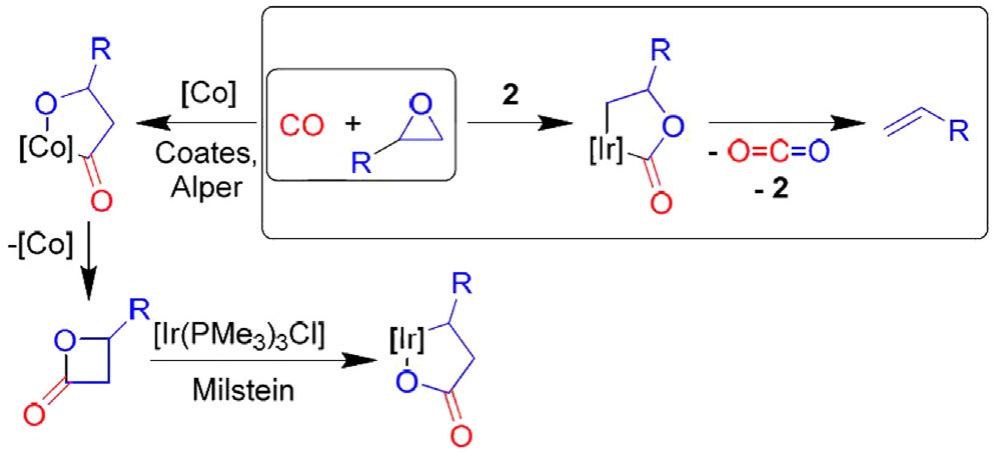
Literature known formation of β‐lactones (left) and their C−O activation with iridium(I) complexes (bottom). The deoxygenation of epoxides (right, this work) proceeds via a new isomeric iridalactone which does not reductively eliminate β‐lactones.

As β‐lactones are known to easily eliminate CO_2_ under elevated temperatures or Lewis acidic conditions, the reductive elimination of β‐lactones from intermediate **6** and subsequent CO_2_ elimination could also be a possible way to obtain the olefin. However, as we observed olefin formation from intermediate **5 a** in the presence of a Lewis acid already at room temperature without detecting any signals of a β‐lactone, we are convinced that the olefins are formed by direct CO_2_ elimination from intermediate **6**. In addition, literature known syntheses of β‐lactones often proceed under elevated temperatures as well. Moreover, we could not observe the reverse reaction, the oxidative addition at **2**, neither with nor without the presence of the Lewis acid, and only very slow deoxygenation at 80 °C of the β‐lactones to the olefin (after several days). In contrast, Milstein and co‐workers have shown that electron rich 16 VE iridium(I) complexes oxidatively add β‐propriolactones readily at low temperatures by C_alkyl_−O bond cleavage, thus forming 4‐irida‐γ‐lactones (Scheme 4, bottom).^43^ C−C_acyl_ bond activation, which would form 2‐irida‐γ‐lactones (like in **5**), had not been observed.

To answer the question about the particularity of our systems **1** and **2** in comparison with Co_2_CO_8_ catalyzed CO/epoxide reactions that produce polymers or β‐lactones is the fact that no migratory insertion step of the alkyl group to the carbonyl ligand is involved after epoxide opening as the molecular structure of intermediate **5** revealed. Fast CO migratory insertion after epoxide opening and C−O bond formation to obtain a 2‐metallaoxolan‐3‐one (Scheme 4, left) is usually considered the key step in the formation of β‐lactones.^11^, ^30^ In contrast, a nucleophilic attack of the alkoxide O‐atom at the CO ligand forms the 2‐irida‐γ‐lactone in our case (Scheme 4, right). This can be explained with a stronger Ir‐alkyl bond. It is known that migratory insertion to form acyl ligands occurs slowly in iridium complexes and is e.g., the rate limiting step in the Cativa process.^44^, ^45^

## Conclusion

We presented a new homogeneous catalyzed deoxygenation of epoxides that uses CO directly as a traceless and environmentally benign deoxygenation agent. Especially terminal alkyl epoxides react smoothly and without significant isomerization to the internal olefins. Internal epoxides react under either retention or inversion of the configuration, depending on their substituents.^46^ This can be explained by two different modes of the epoxide opening. Either by an oxidative addition of the epoxy‐CO bond which leads to retention of the configuration or by an S_N_2‐pathway under inversion of the configuration. Various iridalactones **5** were isolated and in some cases structurally characterized. Under stoichiometric conditions, the coordination of the Lewis acid to **5** forming **6** is observed, from which the olefin is released. Thus, the role of the Lewis acid is not only pre‐activation of the epoxide, but also inducing the CO_2_ elimination to produce the product olefin.

## Experimental Section

### General

Unless otherwise stated, all reactions were carried out under an argon atmosphere in dried and degassed solvents using Schlenk technique. Toluene, pentane, were purchased from Sigma Aldrich and dried using an MBraun SPS‐800 solvent purification system. All lithium salts used were obtained from commercial suppliers, dried in vacuum and used without further purification. Chemicals from commercial suppliers were degassed through freeze‐pump‐thaw cycles prior to use. Carbon monoxide was purchased from Westfalen with a purity of 99.97 %. High pressure NMR scale experiments were performed in heavy or medium wall pressure valve NMR tubes (Wilmad). ^1^H NMR spectra of catalytic experiments were recorded with an increased delay time d_1_ of 60 s to insure reliable integration values. See Supporting Information for the numbering scheme of the compounds.

### Synthesis and characterization

**Synthesis of the catalyst [Ir(bimcaMe)(CO)] (2)**: Benzyl potassium (58.6 mg, 450 μmol) and [Ir(acac)(CO)_2_] (52.1 mg, 150 μmol) were added to a suspension of HbimcaMe⋅2 HI (104.3 mg, 150 μmol) in 12 mL of toluene at room temperature and stirred for 24 h. The resulting yellow suspension was filtered, and the filtrate dried in vacuo to obtain the desired product as a yellow solid (96 mg, 91 %). ^1^H NMR (400 MHz, C_6_D_6_): *δ*=1.54 (s, 18 H, H‐11), 3.81 (s, 6 H, H‐14), 6.14 (d, *J=*2.2 Hz, 2 H, H‐2), 7.31 (d, *J=*2.2 Hz, 2 H, H‐4), 7.64 (d, *J=*1.6 Hz, 2 H, H‐4’), 8.48 (d, *J=*1.6 Hz, 2 H, H‐5’). The NMR data in thf‐d_8_ is identical to that of a sample obtained with a Li‐base^27^ however, using benzyl potassium gives a much cleaner reaction.

**General procedure for the catalytic deoxygenation**: **2** (3.3 mg, 5.0 μmol), lithium bis(trifluoromethylsulfonyl)imide (8.6 mg, 30 μmol) and a certain amount of 1,3,5‐trimethoxybenzene as internal standard were dissolved in 0.5 mL of [D_6_]benzene or [D_8_]toluene in a pressure NMR tube. Then 100 μmol of epoxide were added and the NMR tube was pressurized with 10 bar CO, and heated in an oil bath at 80 °C, if not otherwise noted. The yield was determined via ^1^H NMR.

**General procedure for the 1 mmol‐scale catalytic deoxygenation**: **2** (33 mg, 50 μmol), lithium bis(trifluoromethylsulfonyl)imide (86 mg, 0.30 mmol) were dissolved in 5 mL of benzene in a 100 mL Schlenk flask. Then 1.0 mmol of epoxide was added, the argon atmosphere exchanged for CO at ambient pressure and the reaction mixture heated in an oil bath at 80 °C for 3 days. The product was then vacuum transferred from the reaction into a trap cooled with liquid nitrogen and solvent then distilled off. Due to the lack of fractional distillation the product still contains 0.5 equivalents of benzene and losses of about 20 % of product in the benzene fraction.

**Synthesis of 5 a**: **2** (6.6 mg 10 μmol) and epoxypropane (1.4 μL, 20 μmol) were dissolved in 0.5 mL of C_6_D_6_ in a pressure NMR tube and pressurized with 10 bar CO. The reaction mixture was heated to 80 °C for 10 d. The solvent was evaporated and the residue extracted with DCM. After concentration to dryness the residue was washed with pentane to obtain **5 a** as a pale‐yellow solid (Yield: NMR: 89 %; isolated: 1.6 mg, 23 %). ^1^H NMR (C_6_D_6_, 500 MHz): *δ*=1.27 (d, ^3^ *J*_HH_=6.0 Hz, 3 H, H‐20), 1.50 (s, 9 H, H‐11), 1.51 (s, 9 H, H‐13), 1.71 (dd, ^2, 3^ *J*_HH_=11.0, 5.8 Hz, 1 H, H‐19), 1.80 (dd, ^2, 3^ *J*_HH_=11.0 Hz, 11.0 Hz, 1 H, H‐19), 3.77 (s, 3 H, H‐14), 3.89 (s, 3 H, H‐15), 3.92 (ddq, ^3^ *J*_HH_=11.0, 5.8 Hz, 6.0 Hz, 1 H, H‐18), 5.91 (d, ^3^ *J*_HH_=2.2 Hz, 1 H, H‐4’ or 9’), 5.93 (d, ^3^ *J*_HH_=2.2 Hz, 1 H, H‐4’ or 9’), 7.17 (d, ^3^ *J*_HH_=2.2 Hz, 1 H, H‐5’ or 10’), 7.20 (d, ^3^ *J*_HH_=2.2 Hz, 1 H, H‐5’ or 10’), 7.42 (d, ^4^ *J*_HH_=1.5 Hz, 1 H, H‐2 or 7), 7.43 (d, ^4^ *J*_HH_=1.5 Hz, 1 H, H‐2 or 7), 8.37 (d, ^4^ *J*_HH_=1.5 Hz, 1 H, H‐4 or 5), 8.38 (d, ^4^ *J*_HH_=1.5 Hz, 1 H, H‐4 or 5). ^13^C NMR (C_6_D_6_, 125 MHz): *δ*=23.7 (C20), 27.6 (C19), 32.8 (C11+C13), 35.3 (C10+C12), 40.6 (C15), 41.1 (C14), 78.8 (C18), 110.7, 110.8 (C2, C7), 115.8, 116.0 (C4, C5), 117.1, 117.1 (C5’, C10’), 124.7 (C4’), 125.1 (C1+C8), 125.3 (C9’), 128.0 (C4a+5a), 134.6, 135.9 (C1a, C8a), 138.8, 138.9 (C3, C6), 147.5 (C7’), 148.6 (C2’), 172.6 (C16), 182.8 (CO). ESI^+^ (MeCN): *m*/*z* 718.3 [M‐CO+H]^+^. Anal. Calcd. for C_33_H_38_IrN_5_O_3_: C, 53.21; H, 5.14; N, 9.40. Found: C, 53.27; H, 5.24; N, 9.52. IR (ATR, cm^−1^): 2014 (m, CO), 1630 (w, lactone).

**Synthesis of the Intermediates** ***cis*** **‐ and** ***trans*** **‐5 z**: **2** (9.9 mg, 15 μmol) and 2.8 mg (15 μmol) of either *cis*‐ or *trans*‐diethyl epoxy succinate (**3 z**) were dissolved in 0.5 mL of C_6_D_6_ in a pressure NMR tube and pressurized with 10 bar CO. The reaction mixture was heated to 80 °C for 1 d. Single crystals suitable for X‐ray diffraction were obtained by evaporation of the solvent at room temperature.

***cis*** **‐5 z**: ^1^H NMR ([D_8_]Tol, 600 MHz): *δ*=−0.11 (t, *J=*7.2 Hz, 3 H, H‐29), 0.87 (t, *J=*7.0 Hz, 3 H, H‐24), 1.46 (s, 9 H, H‐11), 1.49 (s, 9 H, H‐13), 2.43 (dq, *J=*10.5, 7.2 Hz, 1 H, H‐28), 3.15 (dq, *J=*10.5, 7.2 Hz, 1 H, H‐28), 3.44 (d, *J=*6.8 Hz, 1 H, H‐19), 3.78 (s, 3 H, H‐14/15), 3.85 (dq, *J=*10.9, 7.0 Hz, 1 H, H‐23), 4.07 (dq, *J=*10.9, 7.0 Hz, 1 H, H‐23), 4.09 (s, 3 H, H‐14/15), 4.25 (d, *J=*6.8 Hz, 1 H, H‐18), 6.33 (s, 2 H, H‐5’ and 10’), 7.42 (d, *J=*1.7 Hz, 1 H, H‐4’ or 9’), 7.47 (s, 2 H, H‐4/5 or 2/7), 7.49 (d, *J=*1.7 Hz, 1 H, H‐4’ or 9’), 8.32 (d, *J=*1.1 Hz, 1 H, H‐4/5 or 2/7), 8.32 (d, *J=*1.1 Hz, 1 H, H‐4/5 or 2/7). ^13^C NMR ([D_8_]Tol, 151 MHz): *δ*=13.0 (C29), 14.6 (C24), 32.7, 32.8 (C11, C13), 35.3 (C10+C12), 39.4 (C19), 41.2, 41.7 (C14, C15), 59.1 (C28), 60.5 (C23), 79.8 (C18), 110.7, 110.8, 115.6, 116.2 (C2+C4+C5+C7), 116.4, 117.9 (C5’, C10’), 124.2, 124.8 (C1+C8 or C4a+C5a), 125.8, 127.0 (C4’+C9’), 127.9, 128.0 (C1+C8 or C4a+C5a), 134.1, 134.9 (C1a+C8a), 139.0 (C3+C6), 144.3, 144.7 (C2’, C7’), 168.8 (C16), 170.4 (C21), 179.3 (CO), 180.3 (C26). ESI^+^ (MeCN): *m*/*z* 875.28 [M]^+^, 848.31 [M‐CO+H]^+^. IR (ATR, cm^−1^): 2034 (s, CO), 1747 (m, ester), 1691 (m, ester), 1645 (m, lactone).

***trans*** **‐5 z**: ^1^H NMR (C_6_D_6_, 400 MHz): *δ*=−0.17 (t, *J=*7.2 Hz, 3 H, H‐29), 0.76 (t, *J=*7.2 Hz, 3 H, H‐24), 1.47 (s, 9 H, H‐11 or 13), 1.48 (s, 9 H, H‐11 or 13), 2.36 (dq, *J=*10.6, 7.1 Hz, 1 H, H‐28), 3.20 (dq, *J=*10.5, 7.3 Hz, 1 H, H‐28), 3.58 (dq, *J=*10.4, 7.1 Hz, 1 H, H‐23), 3.60 (d, *J=*3.2 Hz, 1 H, H‐19), 3.76 (dq, *J=*10.7, 7.1 Hz, 1 H, H‐23), 4.04 (s, 3 H, H‐14 or 15), 4.05 (s, 3 H, H‐14 or 15), 5.46 (d, *J=*3.2 Hz, 1 H, H‐18), 6.12 (d, *J=*2.0 Hz, 1 H, H‐4’ or 9’), 6.16 (d, *J=*1.9 Hz, 1 H, H‐4’ or 9’), 7.14 (d, *J=*2.1 Hz, 1 H, H‐5’ or 10’), 7.37 (d, *J=*2.1 Hz, 1 H, H‐5’ or 10’), 7.41 (d, *J=*1.1 Hz, 1 H, H‐2 or 7), 7.43 (d, *J=*1.1 Hz, 1 H, H‐2 or 7), 8.36 (d, *J=*1.3 Hz, 1 H, H‐4 or 5), 8.38 (d, *J=*1.4 Hz, 1 H, H‐4 or 5). ^13^C NMR (C_6_D_6_,101 MHz): *δ*=12.8 (C29), 14.5 (C24), 32.8 (C11+C13), 35.3 (C10+C12), 38.7 (C19), 41.4 (C14+C15), 59.6 (C28), 60.8 (C23), 79.6 (C18), 110.6, 110.7 (C2, C7), 115.7, 115.9 (C4, C5), 116.4, 117.5 (C5’, C10’), 124.7, 124.9 (C4’, C9’), 125.9, 126.4, 127.9, 128.0 (C1, C8, C4a, C5a), 134.6, 134.8 (C1a, C8a), 139.0 (C3+C6), 142.8, 144.5 (C2’, C7’), 168.7 (C16), 173.0 (C21), 181.5, 181.7 (CO, C26). ESI^+^ (MeCN): *m*/*z* 875.28 [M]^+^, 848.31 [M‐CO+H]^+^. IR (ATR, cm^−1^): 2035 (s, CO), 1738 (m, ester), 1683 (m, ester), 1645 (m, lactone).

### X‐ray structure analysis

Data collection was carried out on a Bruker APEX Duo CCD with an Incoatec IμS Microsource with a Quazar MX mirror using Mo K_*α*_ radiation (*λ*=0.71073 Å) and a graphite monochromator. Corrections for absorption effects were applied using SADABS.^47^ All structures were solved by direct methods using SHELXS and refined using SHELXL.^48^ Deposition Numbers 1951759, 1951760, 1951761, and 1951762 (***trans*** **‐5 z**, ***cis*** **‐5 z**, [Ir(bimca)(CO)Br_2_] and [Ir(bimca)(CO)I_2_], respectively) contain the supplementary crystallographic data for this paper. These data are provided free of charge by the joint Cambridge Crystallographic Data Centre and Fachinformationszentrum Karlsruhe Access Structures service www.ccdc.cam.ac.uk/structures.

## Conflict of interest

The authors declare no conflict of interest.

## Supporting information

As a service to our authors and readers, this journal provides supporting information supplied by the authors. Such materials are peer reviewed and may be re‐organized for online delivery, but are not copy‐edited or typeset. Technical support issues arising from supporting information (other than missing files) should be addressed to the authors.

Supplementary
