# Supplementary material for: Deoxygenation of Epoxides with Carbon Monoxide
Source: Chemistry. 2020 Jul 23;26(46):10634–40. doi: 10.1002/chem.202002651 (PMC7496459; doi:10.1002/chem.202002651)
Supplement: Supplementary file 1 — Supplementary [file CHEM-26-10634-s001.pdf]

Chemistry–A European Journal

Supporting Information

Deoxygenation of Epoxides with Carbon Monoxide**

Theo Maulbetsch, Eva Jürgens, and Doris Kunz*[a]

Supporting Information

Table of Contents

1. General information S2
2. Mechanistic studies...S4
3. NMR spectra S9
4. References...S64

1. General information

Unless otherwise stated, all reactions were carried out under an argon atmosphere in dried and degassed solvents using Schlenk technique. Toluene, pentane, were purchased from Sigma Aldrich and dried using an MBraun SPS-800 solvent purification system. All lithium salts used were obtained from commercial suppliers, dried in vacuum and used without further purification. Chemicals from commercial suppliers were degassed through freeze-pump-thaw cycles prior to use. Carbon monoxide was purchased from Westfalen with a purity of 99.97 %. All epoxides were purchased from commercial suppliers, except epoxides **3l**, **3m**, **3o-3t** and **3za** which were synthesized from the respective aldehydes[1], **3k**, **3v**[2], **3x**[3] and **3y**[4] from the olefins, and **3z** which was synthesized from the corresponding acid[5] according to literate procedures. High pressure NMR scale experiments were performed in Wilmad Heavy/Medium Wall Precision Pressure/Vacuum Valve NMR Sample Tubes. ^{1}H and ^{13}C NMR spectra were recorded using a Bruker AVANCE II+ 400 spectrometer, a Bruker AVANCE AVII+ 500 or a Bruker Avance III HDX 600. Chemical shifts δ (ppm) are reported relative to the solvent's residual proton and carbon signal respectively: THF-d_8: 3.58 ppm (^{1}H NMR) and 67.57 ppm (^{13}C NMR); C_6D_6: 7.16 ppm (^{1}H NMR) and 128.39 ppm (^{13}C NMR); DMSO-d_6: 2.50 ppm (^{1}H NMR) and 39.51 ppm (^{13}C NMR), $CDCl_3$: 7.27 ppm (^{1}H NMR) and 77.0 ppm (^{13}C NMR), toluene-d_8: 6.97 ppm (^{1}H NMR) and 125.96 ppm (^{13}C NMR). Coupling constants (*J*) are expressed in Hz. Signals were assigned as s (singlet), d (doublet), t (triplet), q (quartet), m (multiplet) and variations thereof. Assignment of the peaks was made using 2D NMR correlation spectra. ^{1}H NMR spectra of catalytic experiments were recorded without CO atmosphere, after confirming no difference in accuracy, and under CO atmosphere in presence of volatile compounds (indicated with "*" in the manuscript). An increased delay time d_1 of 60 s ensured reliable integration values. The mass spectra were recorded on a Bruker Esquire 3000 Plus ion trap mass spectrometer. IR spectra were recorded with a Bruker Vertex 70 or with a Mettler Toledo ReactIR 15. X-ray structure analysis: Crystallographic data collection was carried out on a Bruker APEX Duo CCD with an Incoatec IµS Microsource with a Quazar MX mirror using Mo K_{α} radiation (λ = 0.71073 Å) and a graphite monochromator.

Corrections for absorption effects were applied using SADABS.[6] All structures were solved by direct methods using SHELXS and refined using SHELXL.[7] CCDC 1951759 (**trans-5z**), 1951760 (**cis-5z**), 1951761 ([Ir(bimca)(CO)Br_2]) and 1951762 ([Ir(bimca)(CO)I_2]) contain the supplementary crystallographic data. These data can be obtained free of charge from the Cambridge Crystallographic Data Centre at www.ccdc.cam.ac.uk/data_request/cif.

Numbering Scheme: For the assignment of the peaks, the following numbering scheme was used.

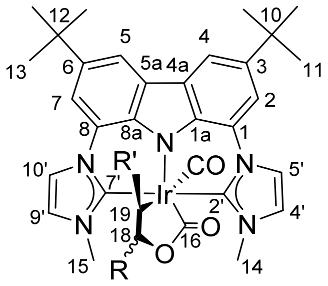

5a: R = CH_3 (21), R' = H

cis,trans-5z:

R =

R' =

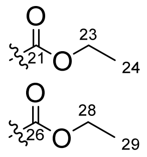

2. Mechanistic studies

Reaction of 5a with $LiNTf_2$

$LiNTf_2$ (2.8 mg, 10 µmol) was added to a solution of the Intermediate **5a** (7.4 mg, 10 µmol) in C_6D_6 and the reaction was observed via ^1H NMR spectroscopy.

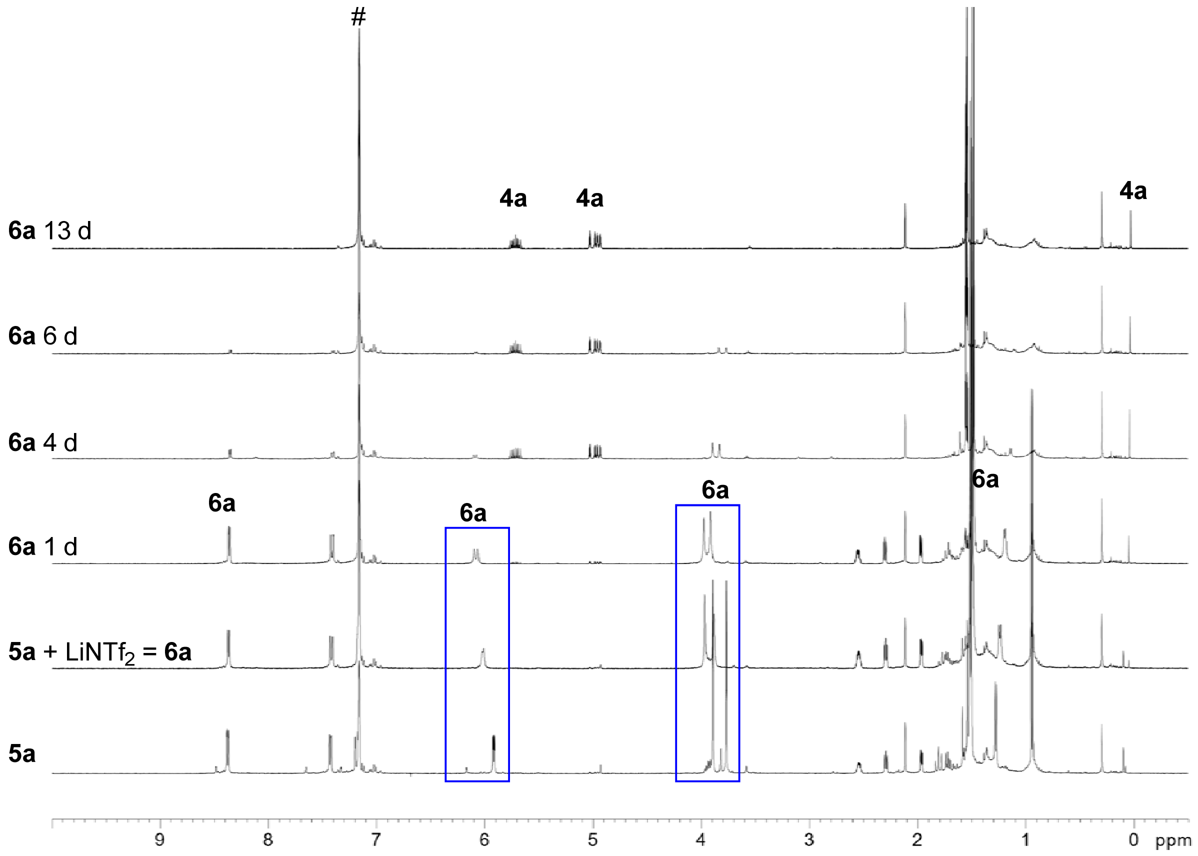

Figure S1. ^1H *NMR (C_6D_6 (#), 400 MHz) spectra:* ***5a*** *with $LiNTf_2$ at room temperature. Addition of $LiNTf_2$ leads to adduct* ***6a****, indicated by a shift of signals of the N-CH_3 group and the adjacent imidazole protons (blue squares). Afterwards, slow formation of propene (**4a**) by decarboxylation is detected already at room temperature.*

Selectivity and isomerization of 4j and 4z

In the case of **4j** no *cis*/*trans* isomerization is observed at a reaction temperature of 80 °C. At 120 °C slow isomerization is observed. After 24 h 6% of the total amount of olefin **4j** are the *trans*-isomer starting from ***cis*-3j** and 4% are the *cis*-isomer when starting form ***trans*-3j**. After full conversion of ***cis*-3j** (after 72 h) the amount of ***trans*-4j** increased to 22 % due to the ability of **2** to catalyze olefin-isomerization (*vide infra* for 1-hexene). ***Trans*-3j** was not fully converted after 72 h the isomerized product amounts to 4 % of the total amount of the olefin.

Similar observations were made for **4z**. At 80 °C a ratio ***cis*-4z** : ***trans*-4z** of 81 : 19 was observed starting from ***trans*-3z** and a ratio ***cis*-4z** : ***trans*-4z** of 18 : 82 when ***cis*-3z** is reacted. At the elevated temperature (120 °C) the isomerization was more pronounced for ***trans*-3z** (***cis*-4z** : ***trans*-4z** = 61 : 39) and unchanged for ***cis*-3z** (***cis*-4z** : ***trans*-4z** of 18 : 82) after full conversion of the epoxides.

Reaction Monitoring

A catalytic experiment was set up according to the general procedure with propylene oxide as substrate. After pressurizing with carbon monoxide, the thick-wall NMR tube is placed into the spectrometer and heated to 60 °C. The temperature was maintained for 23 hours and ^{1}H NMR spectra were recorded every 11 minutes during this time.

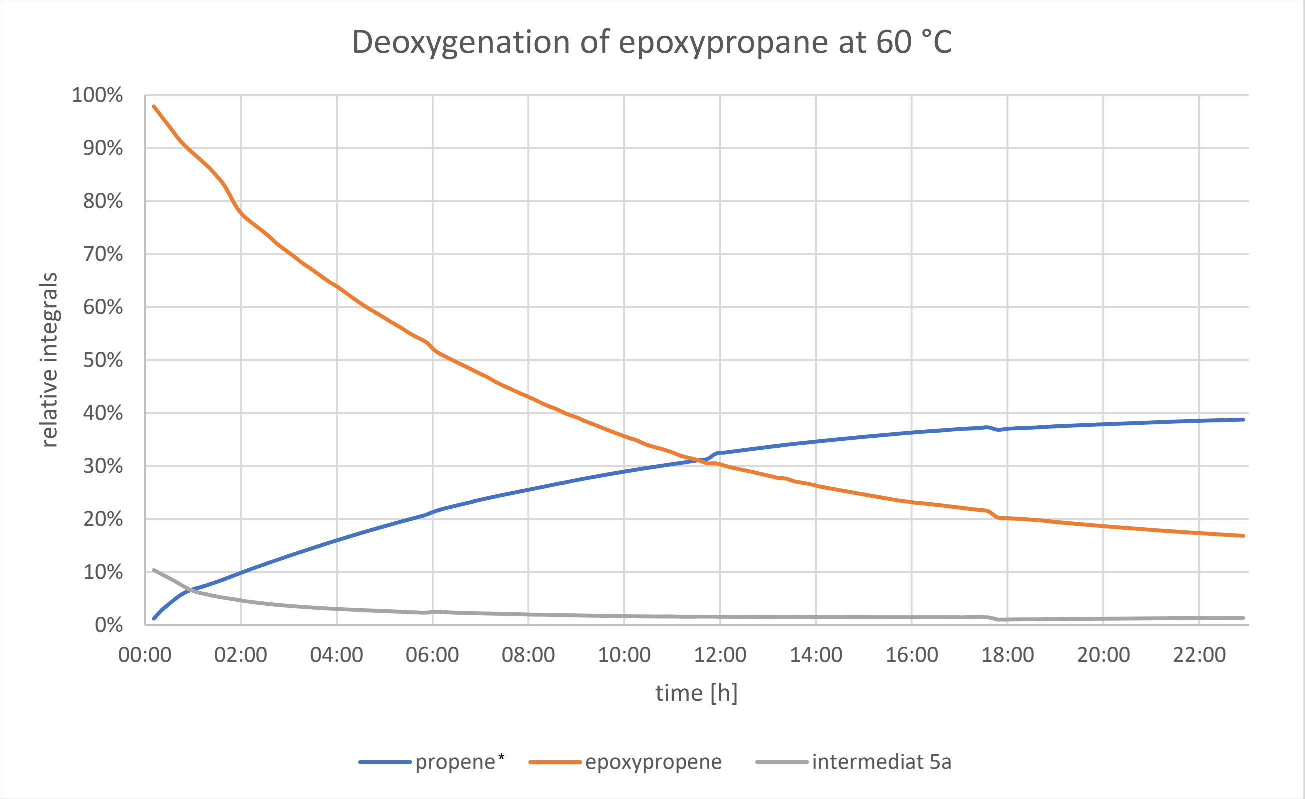

* only the amount of dissolved propene was recorded.

Figure S2. Monitoring of the deoxygenation reaction of propylene oxide by ^{1}H NMR spectroscopy.

Deactivation of the catalyst

When trying to suppress the aldehyde formation in the case of styrene oxides by using weaker Lewis acids such as LiI or LiBr (with and without addition of thf), the reaction did not go to completion. Instead we could observe the formation of [Ir(bimca)(CO)I_2] or [Ir(bimca)(CO)Br_2] respectively. Single crystals suitable for X-ray diffraction were obtained by slow cooling of the solution down to room temperature.

[Ir(bimca)(CO)I_2]: ^{1}H NMR (THF-d_8, 400 MHz): δ = 1.56 (s, 18 H, H-11), 4.28 (s, 6 H, H-12), 7.60 (d, $^{3}J_{HH}$ = 2.3 Hz, 2 H, H-4'), 7.80 (d, $^{4}J_{HH}$ = 1.6 Hz, 2 H, H-2/7), 8.15 (d, $^{4}J_{HH}$ = 1.6 Hz, 2 H, H-4/5), 8.44 (d, $^{3}J_{HH}$ = 2.3 Hz, 2 H, H-5'). **^{13}C NMR** (THF-d_8, 100 MHz): δ = 32.7 (C-11), 35.7 (C-10), 42.3 (C-12), 111.7 (C-2), 115.8 (C-4), 118.2 (C-5'), 125.0 (C-1a), 126.7 (C-4'), 128.6 (C-4a), 134.9 (C-1), 140.4 (C-3), 142.9 (C-2'), 170.2 (CO).

[Ir(bimca)(CO)Br_2]: ^{1}H NMR (DMSO-d_6, 400 MHz): δ = 1.51 (s, 18H, H-11), 4.25 (s, 6H, H-12), 7.93 (br s, 4H, H-4' und H-2/7), 8.21 (d, $^{4}J_{HH}$ = 1.5 Hz, 2H, H-4/5), 8.82 (d, $^{3}J_{HH}$ = 2.3 Hz, 2H, H-5'). **^{13}C NMR** (DMSO-d_6, 100 MHz): δ = 32.0 (C10), 34.8 (C11), 40.4 (C12), 111.5 (C2/7), 115.0 (C4/5), 118.0 (C5'), 119.5 (C4a/5a), 123.4 (C1/8), 126.4 (C4'), 132.9 (C1a/8a), 140.0 (C3/6), 143.0 (C2'), 166.5 (CO).

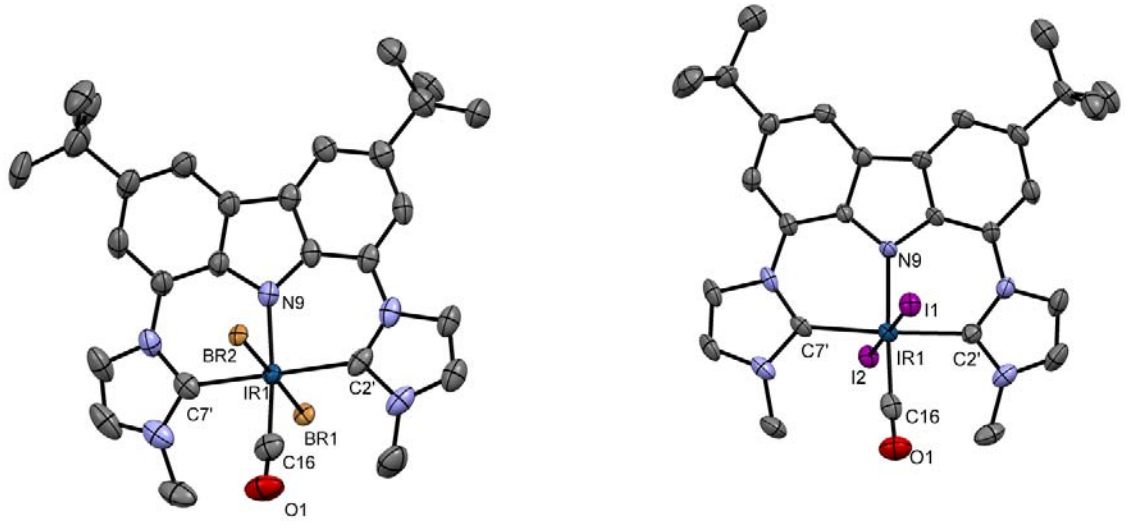

Figure S3. Molecular structure of the side products [Ir(bimca)(CO)Br_2] (left) and [Ir(bimca)(CO)I_2] (right). Atoms are shown with anisotropic atomic displacement parameters at the 50 % probability level. In both cases the hydrogen atoms as well as two co-crystallized benzene molecules are omitted for clarity.

Isomerization of α-Hexane

In further experiments the isomerization of the olefinic product was investigated.

2 (3.3 mg, 5.0 µmol) and/or lithium bis(trifluoromethylsulfonyl)imide (8.6 mg, 30 µmol) with a certain amount of the internal standard 1,3,5-trimethoxybenzene were dissolved in 0.5 mL of benzene-d_6 in a J-Young valve NMR tube. Then 100 µmol of hexene was added and the reaction mixture was kept in an oil bath at 80 °C. The yield was determined by ^{1}H NMR spectroscopy against an internal standard.

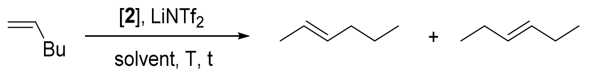

Entry	Cat. [mol%]	$LiNTf_2$ [mol%]	Solvent	Temp. [°C]	Time [d]	Isomer [%]
1	5	--	C_6D_6	80	7	2
2	--	30	C_6D_6	80	7	0
3	5	30	C_6D_6	80	1	15
4	5	30	C_6D_6	80	7	53

The results indicate that the isomerization of the olefin occurs under the same conditions as the deoxygenation. Furthermore, the isomerization requires both in catalytic amounts, the iridium complex and the Lewis acid.

3. NMR spectra

3.1 Catalyst **2** and Intermediates.

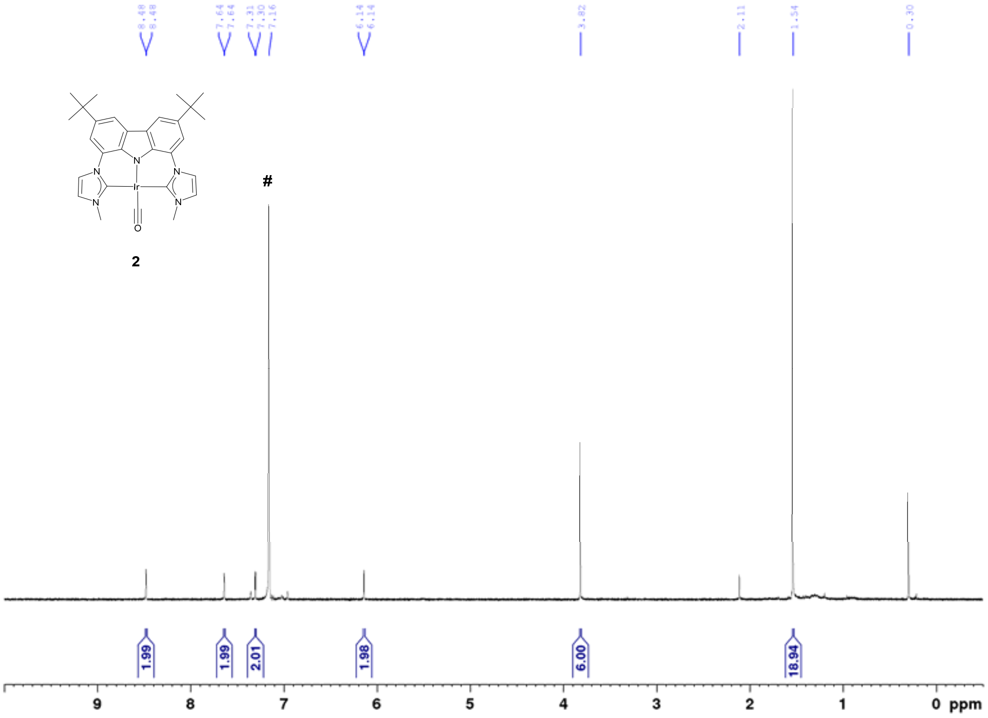

Figure S4. *¹H NMR (C_6D_6 (#), 400 MHz) spectrum of* ***2.***

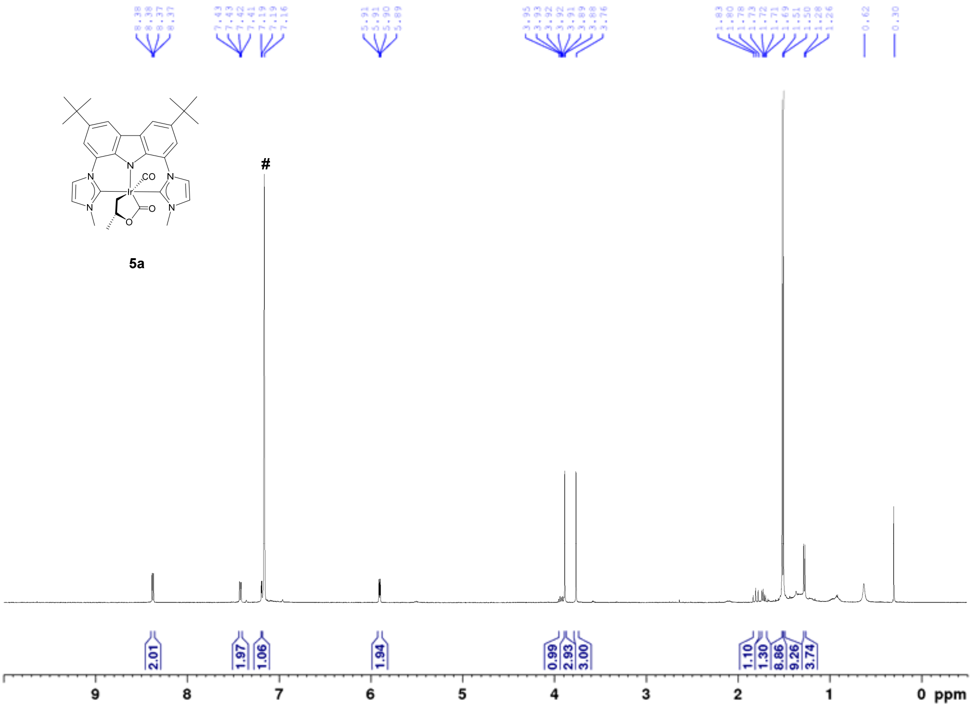

*Figure S5. ¹H NMR (C_6D_6 (#), 400 MHz) spectrum of **5a** (see general information for the peak assignment).*

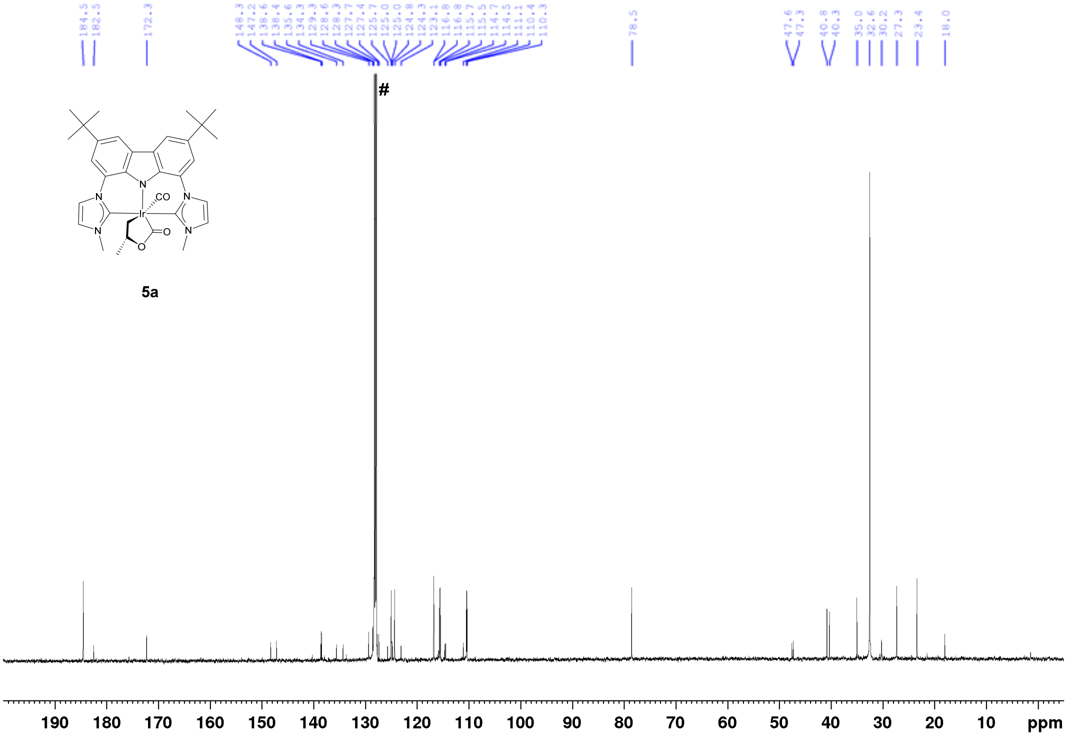

*Figure S6. ¹³C NMR (C_6D_6 (#), 100 MHz) spectrum of **5a** (see general information for the peak assignment).*

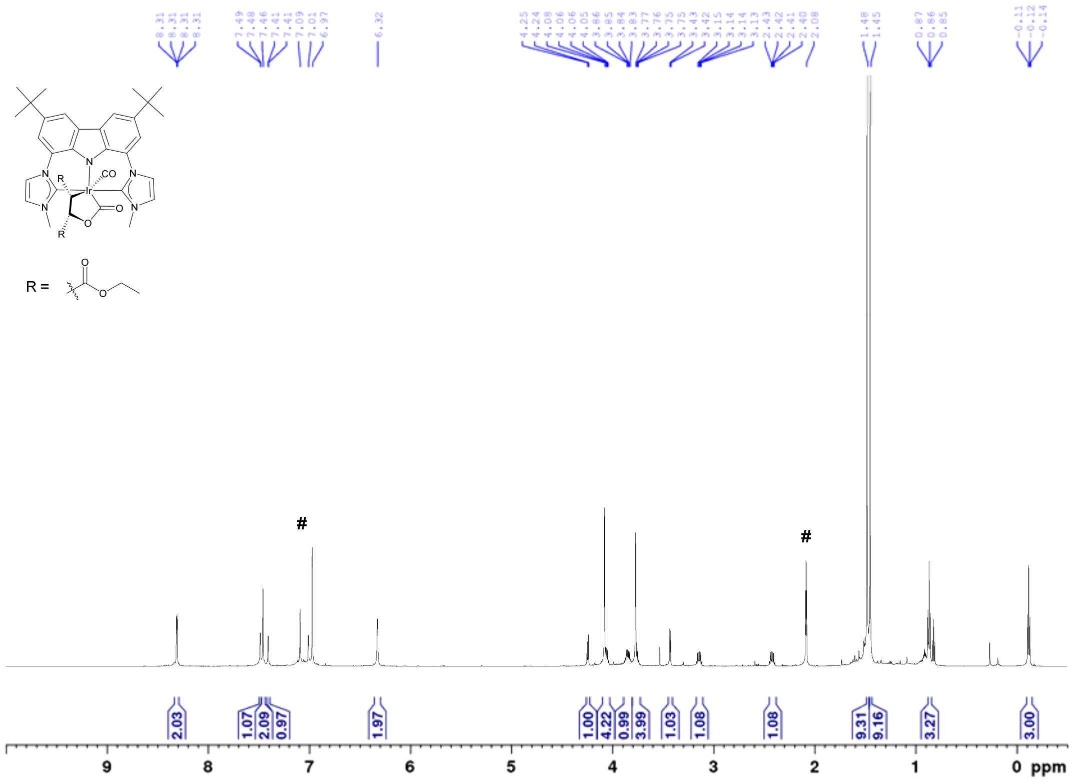

Figure S7. ^{1}H NMR (toluene-d_8 (#), 600 MHz) spectrum of **cis-5z** (see general information for the peak assignment).

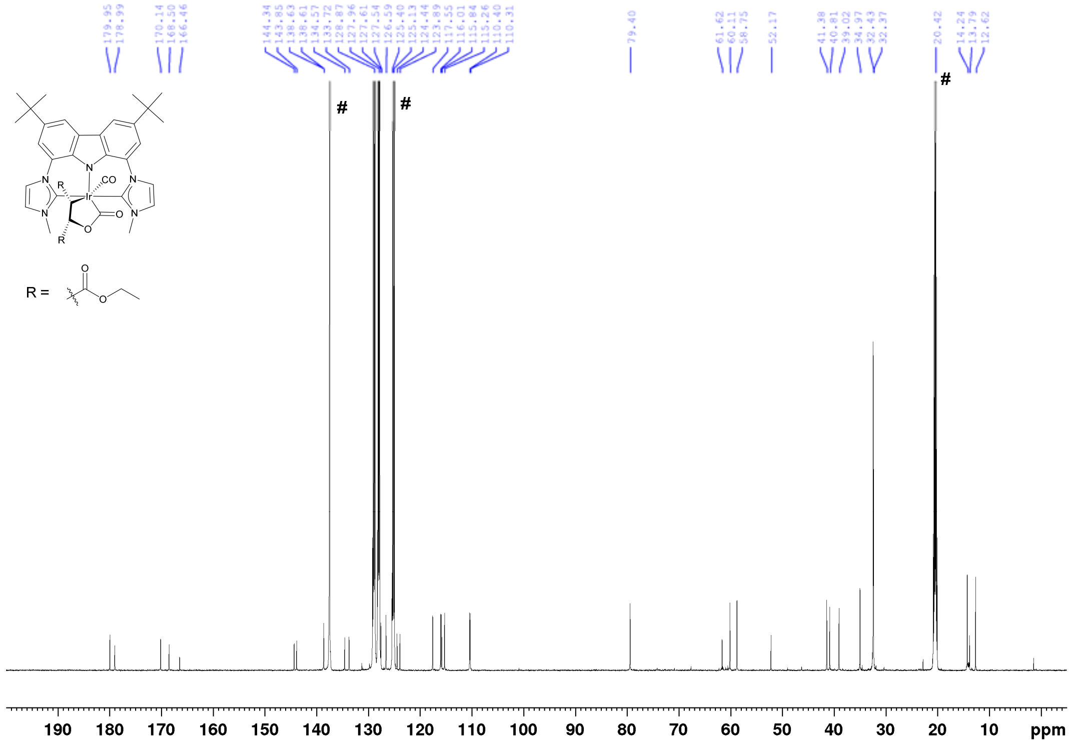

Figure S8. ^{13}C NMR (toluene-d_8 (#), 150 MHz) spectrum of **cis-5z** (see general information for the peak assignment).

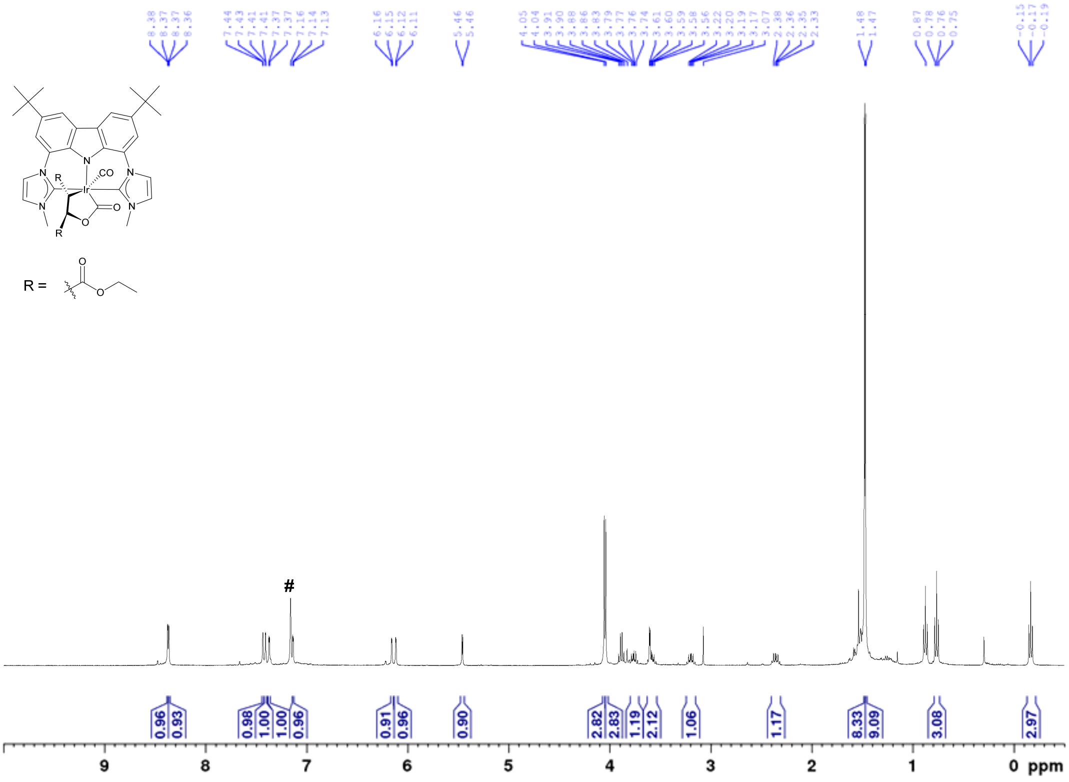

*Figure S9. ^1H NMR (C_6D_6 (#), 400 MHz) spectrum of **trans-5z** (see general information for the peak assignment).*

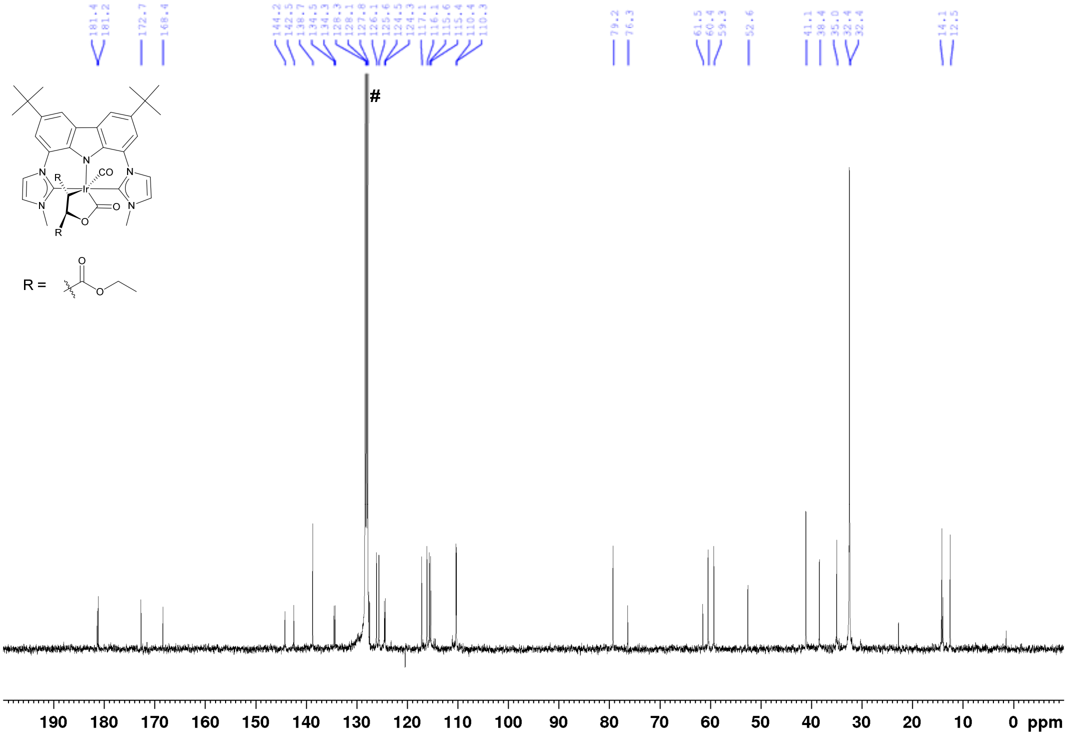

*Figure S10. ^{13}C NMR (C_6D_6 (#), 100 MHz) spectrum of **trans-5z** (see general information for the peak assignment)**.***

3.2 Catalysis experiments

Unless otherwise noted, all catalytic reactions were carried out under standard conditions (at 80 °C with 10 bar CO for 24 h at a 0.2 M epoxide concentration with a 5% catalyst loading).

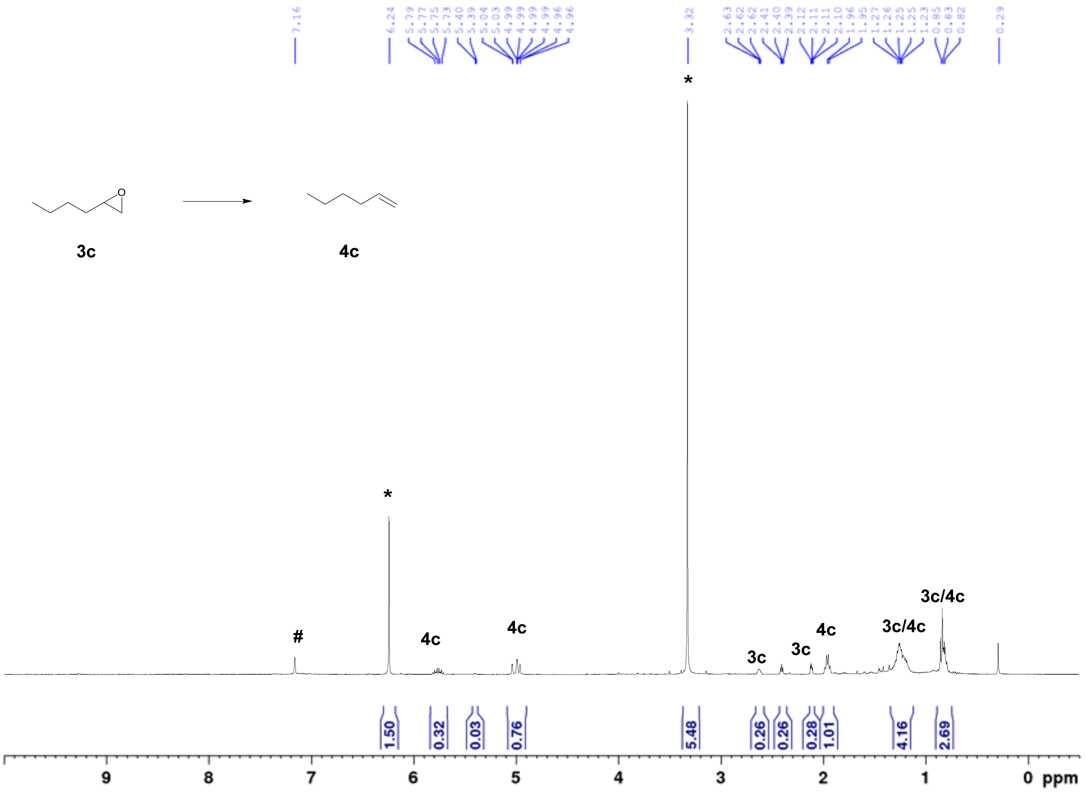

Figure S11. [1]*H NMR (C_6D_6 (#), 400 MHz) spectrum: deoxygenation of* ***3c*** *into* ***4c*** *with* ***1*** *as a catalyst; internal standard (*).*

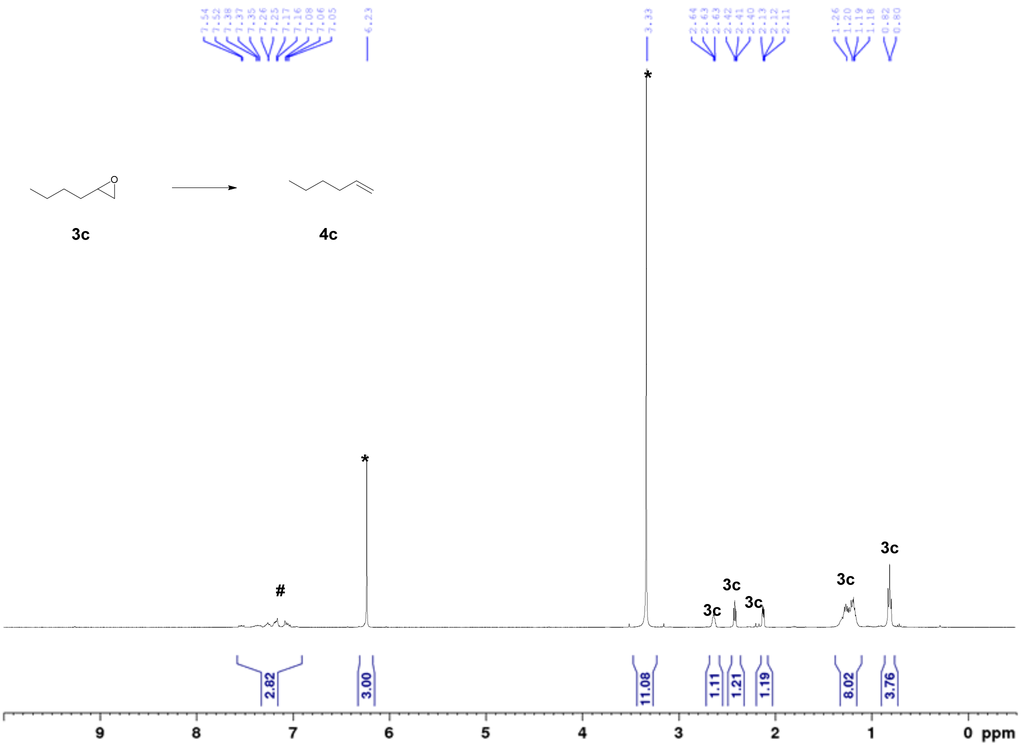

Figure S12.[1]*H NMR (C_6D_6 (#), 400 MHz) spectrum: deoxygenation of* ***3c*** *into* ***4c*** *with [RhCl(PPh_3)$_3$] as a catalyst; internal standard (*).*

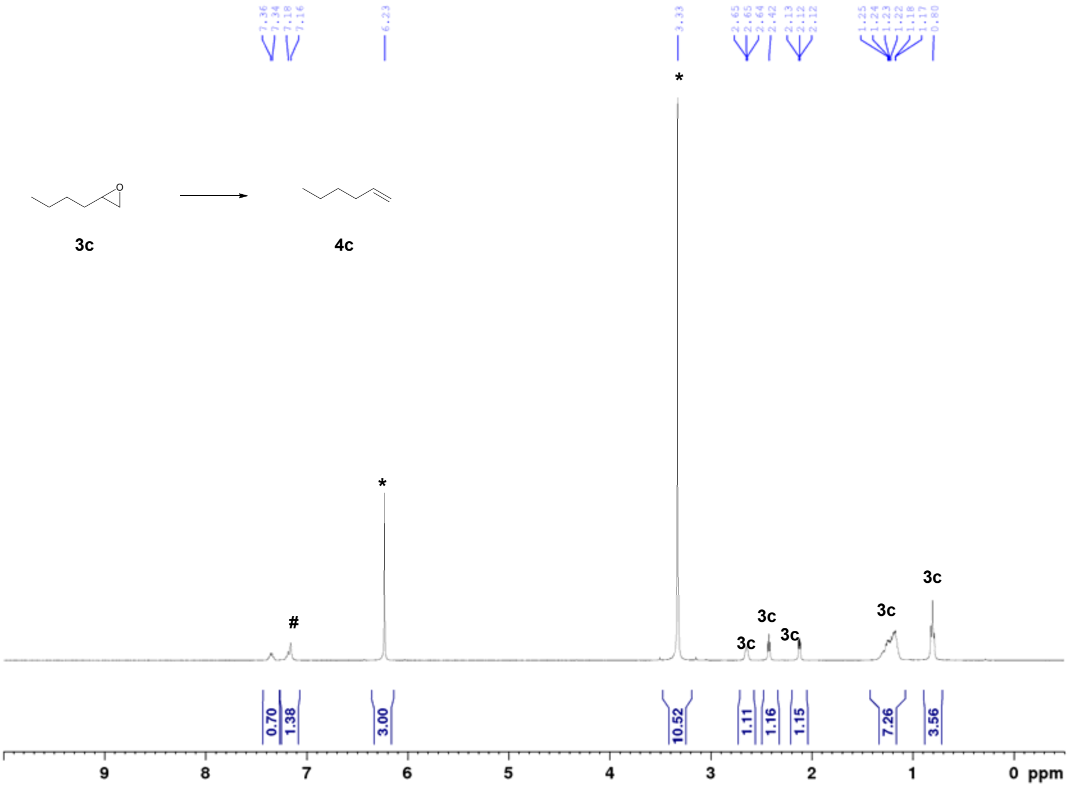

Figure S13[1]*H NMR (C_6D_6 (#), 400 MHz) spectrum: deoxygenation of* ***3c*** *into* ***4c*** *with [Ir(CO)Cl(PPh_3)$_2$] as a catalyst; internal standard (*).*

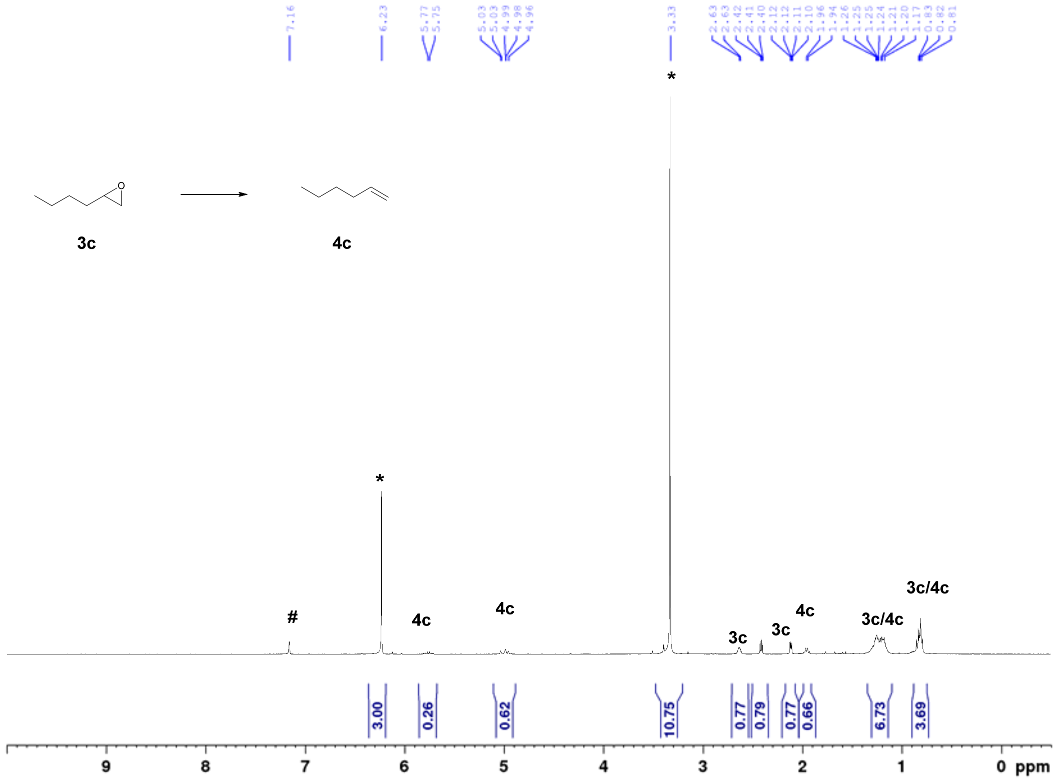

*Figure S14. ^{1}H NMR (C_6D_6 (#), 400 MHz) spectrum: deoxygenation of **3c** into **4c** after 24 **h** with [Ir(acac)(CO)$_2$] as a catalyst; internal standard (*).*

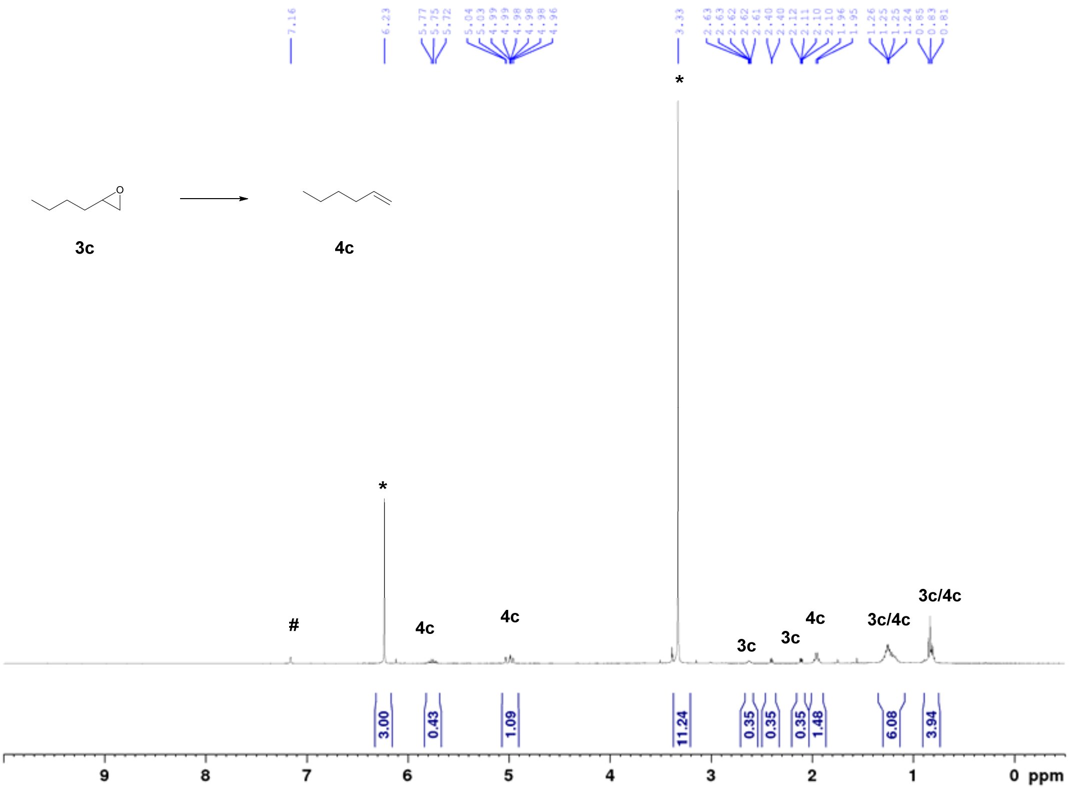

*Figure S15. ¹H NMR (C_6D_6 (#), 400 MHz) spectrum: deoxygenation of **3c** into **4c** after 90 **h** with [Ir(acac)(CO)$_2$] as a catalyst; internal standard (*).*

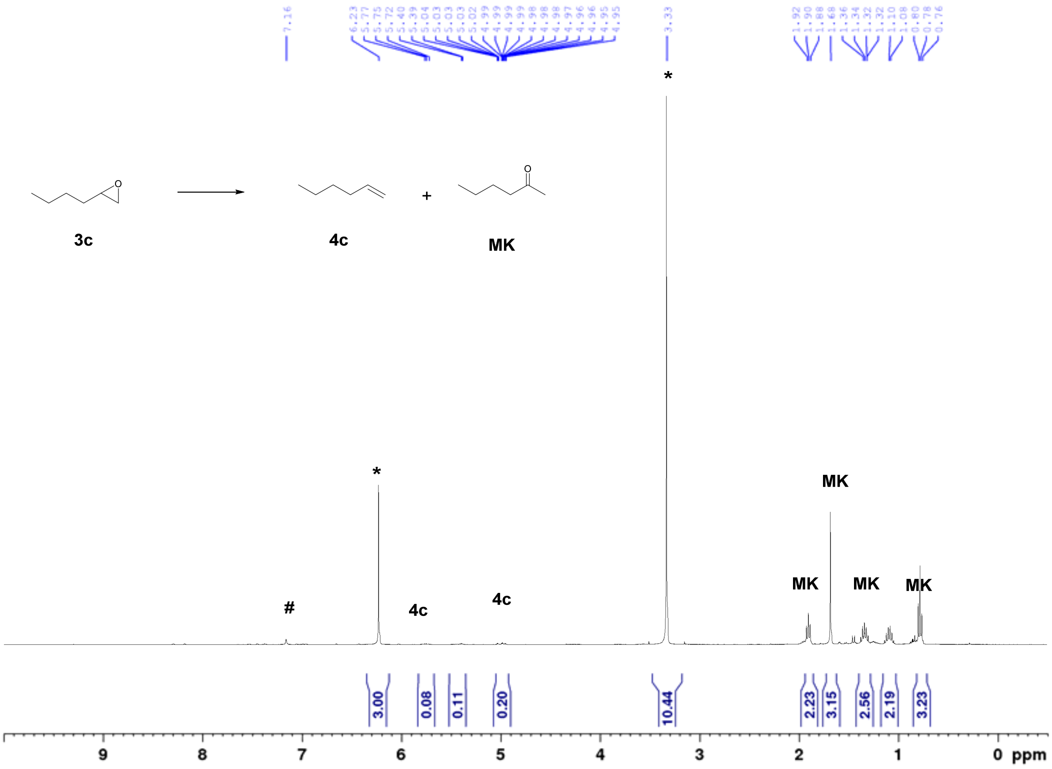

*Figure S16. ^{1}H NMR (C_6D_6 (#), 400 MHz) spectrum: deoxygenation of **3c** into **4c** after 90 **h** with [Ir(bimca^{C5})(CO)] as a catalyst; internal standard (*).*

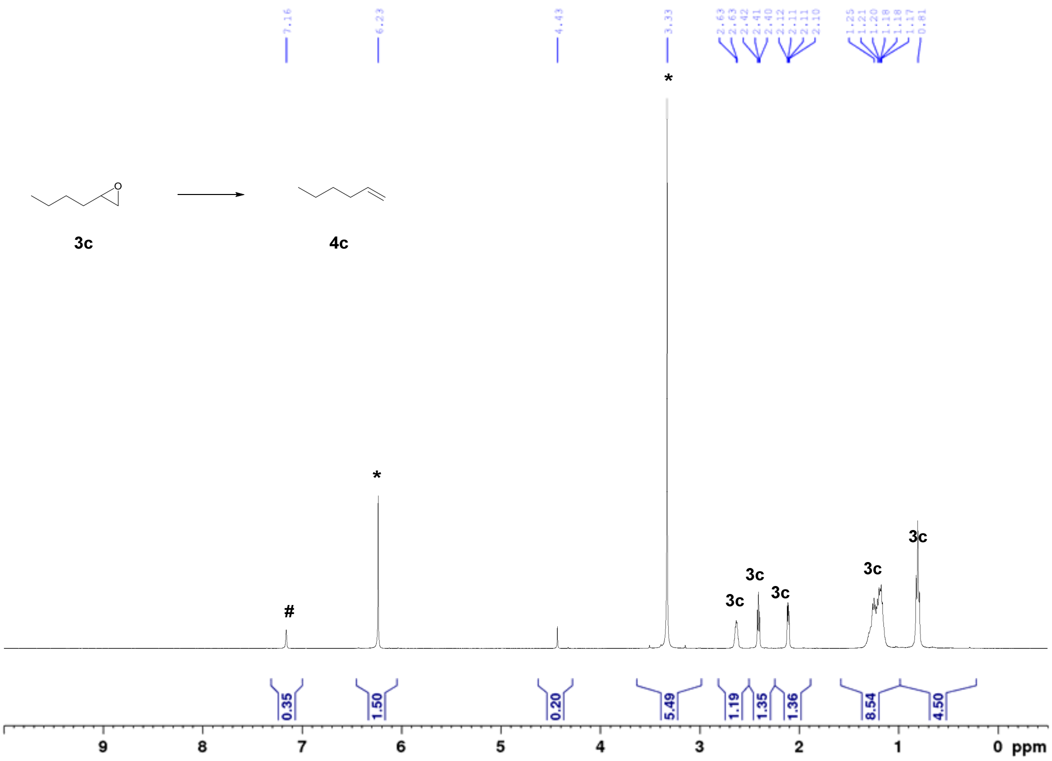

*Figure S17. ^{1}H NMR (C_6D_6 (#), 400 MHz) spectrum: deoxygenation of **3c** into **4c** after 90 **h** with [Co(Cp)(CO)$_2$] as a catalyst; internal standard (*).*

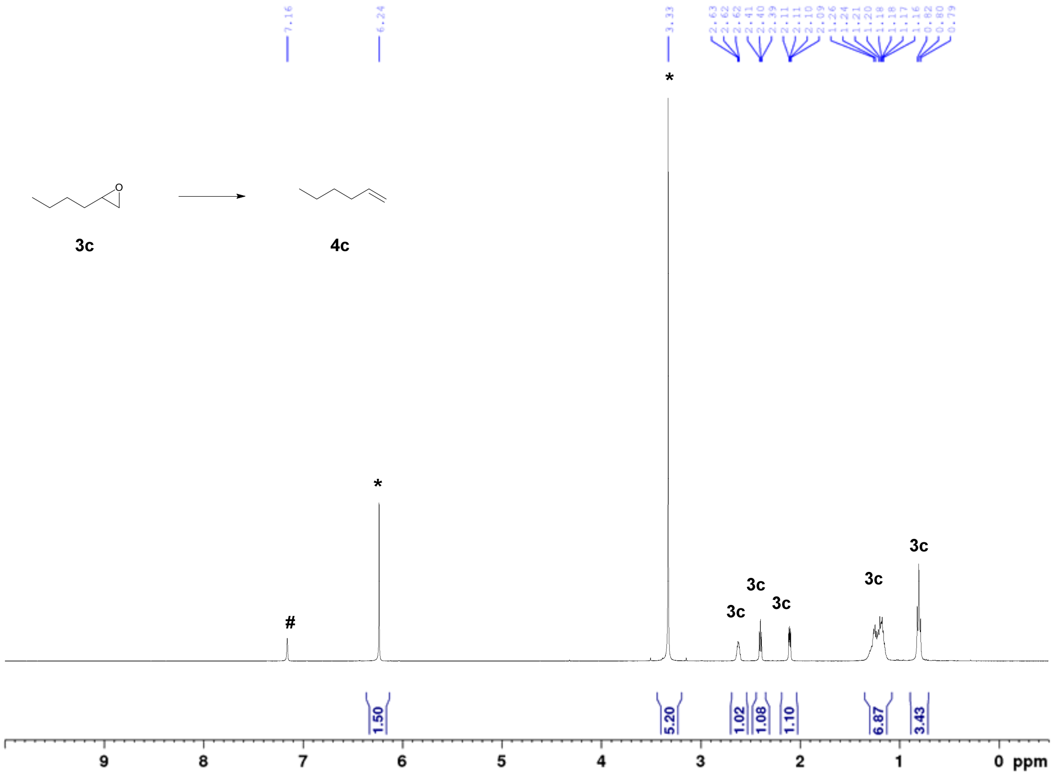

Figure S18. [1]*H NMR (*C_6D_6 *(#), 400 MHz) spectrum: deoxygenation of* ***3c*** *into* ***4c*** *after 90* ***h*** *with [*$Co_2(CO)_8$*] as a catalyst; internal standard (*).*

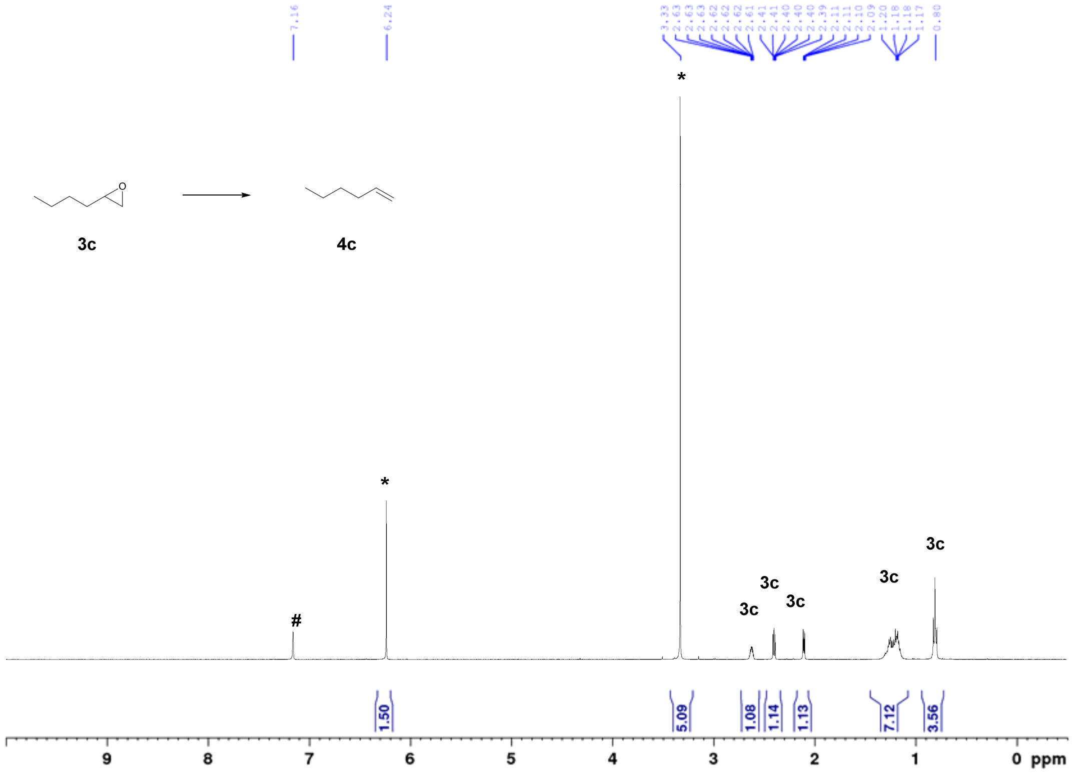

Figure S19. [1]*H NMR (*C_6D_6 *(#), 400 MHz) spectrum: deoxygenation of* ***3c*** *into* ***4c*** *after 90* ***h*** *with [*$Fe(CO)_5$*] as catalyst; internal standard (*).*

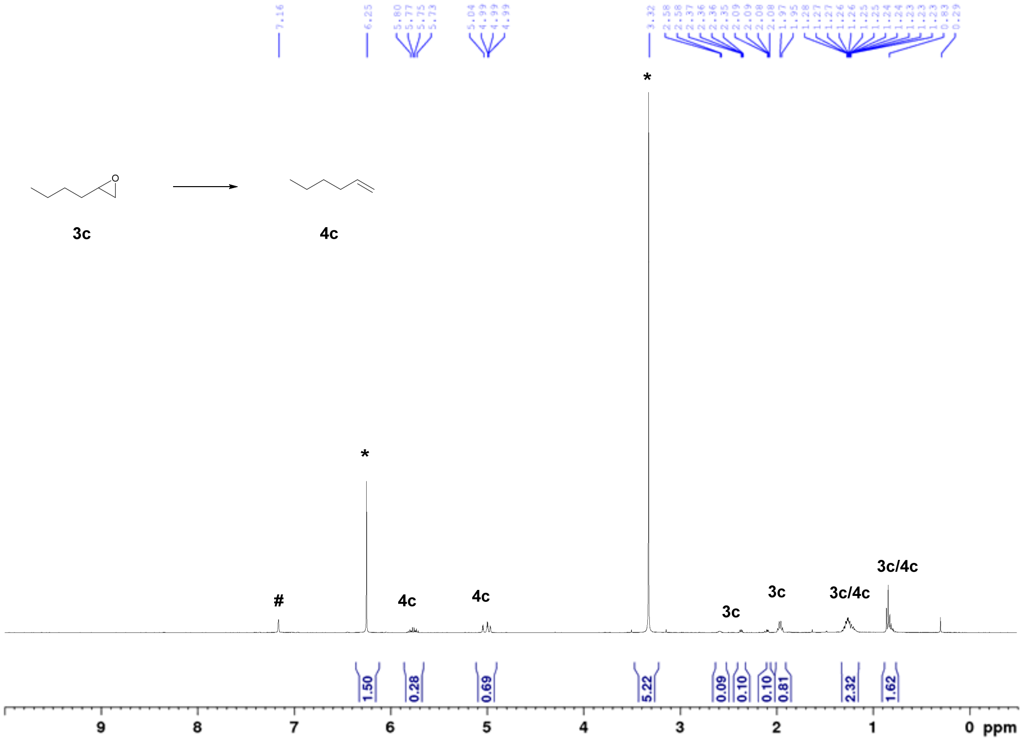

*Figure S20. ^{1}H NMR (C_6D_6 (#), 400 MHz) spectrum: deoxygenation of **3c** into **4c** at 0.1 M concentration; internal standard (*).*

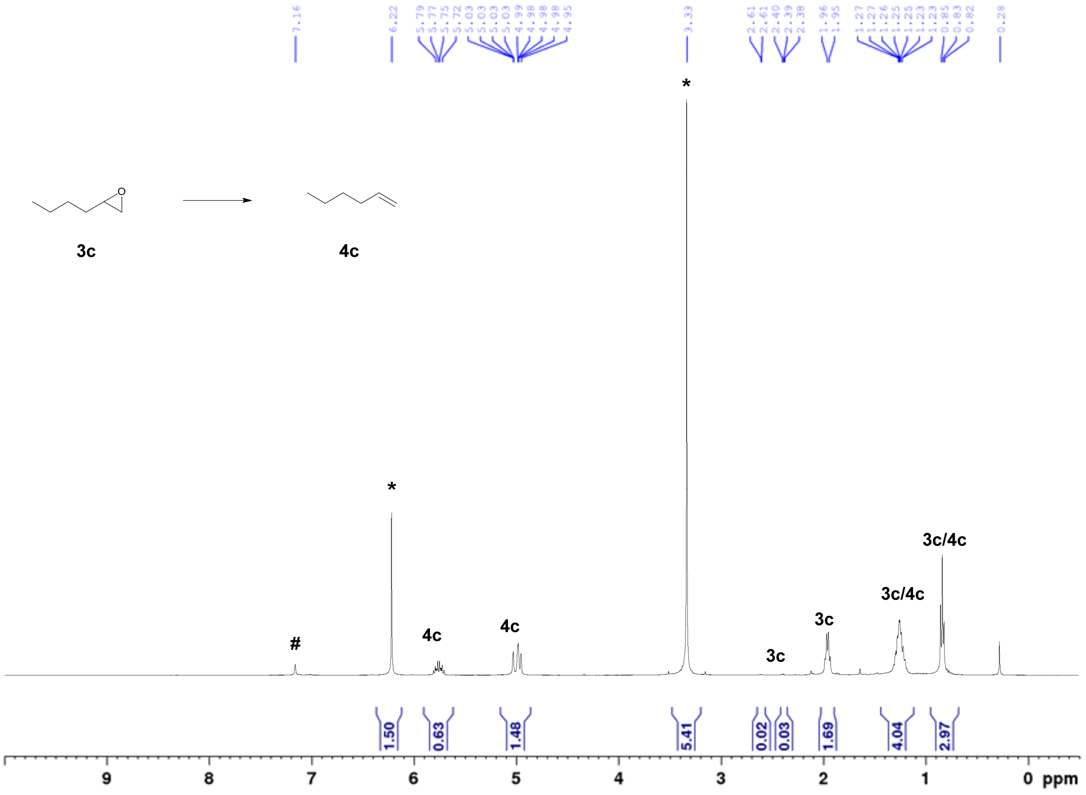

*Figure S21. ^{1}H NMR (C_6D_6 (#), 400 MHz) spectrum: deoxygenation of **3c** into **4c** at 0.5 M concentration; internal standard (*).*

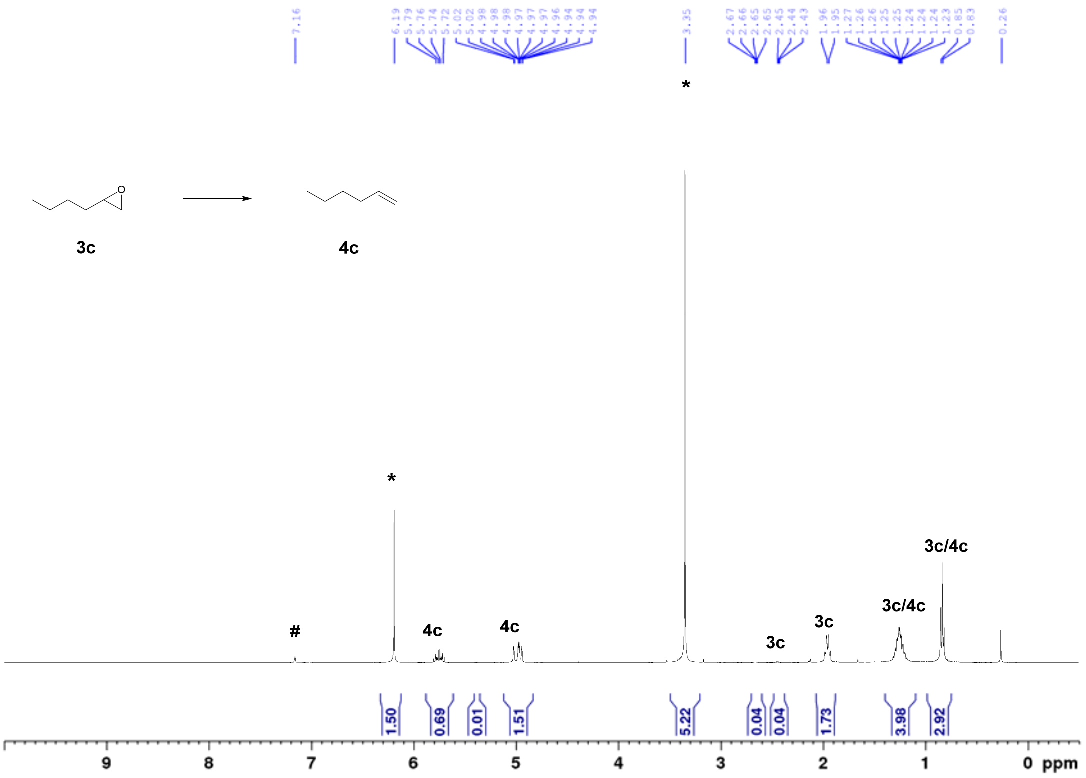

*Figure S22. ¹H NMR (C_6D_6 (#), 400 MHz) spectrum: deoxygenation of **3c** into **4c** at 1.0 M concentration; internal standard (*).*

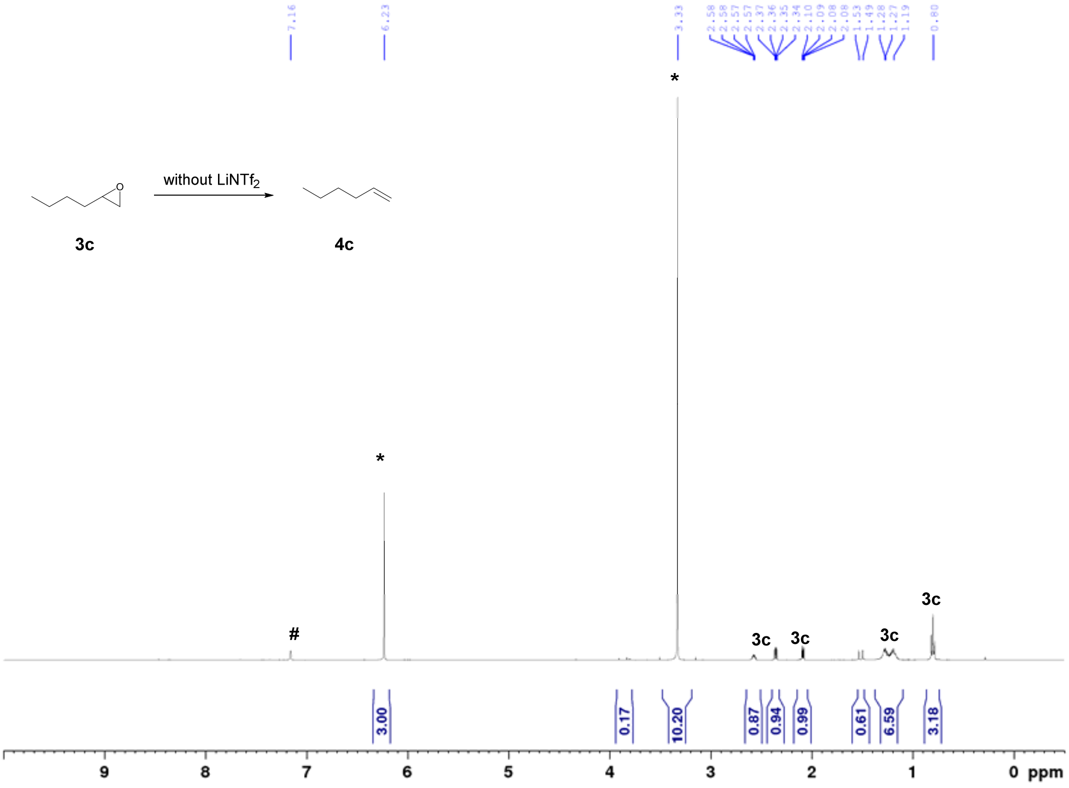

*Figure S23. ¹H NMR (C_6D_6 (#), 400 MHz) spectrum: deoxygenation of **3c** into **4c** without $LiNTf_2$; internal standard (*).*

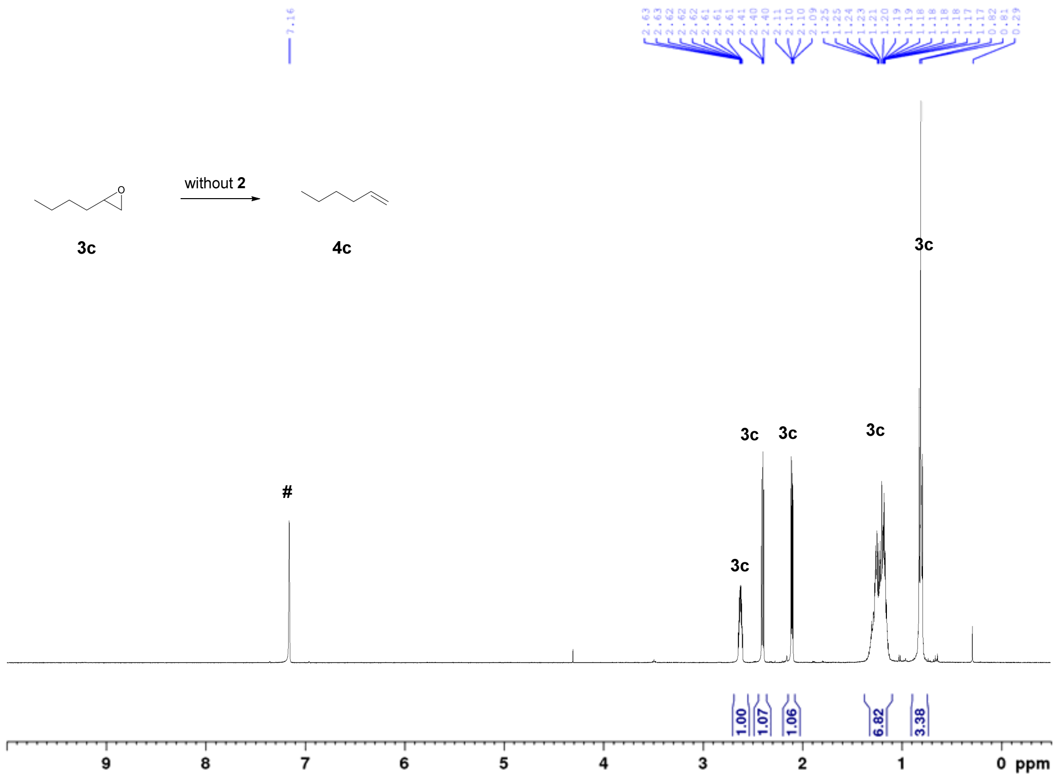

*Figure S24. ¹H NMR (C_6D_6 (#), 400 MHz) spectrum: deoxygenation of **3c** into **4c** without **2**.*

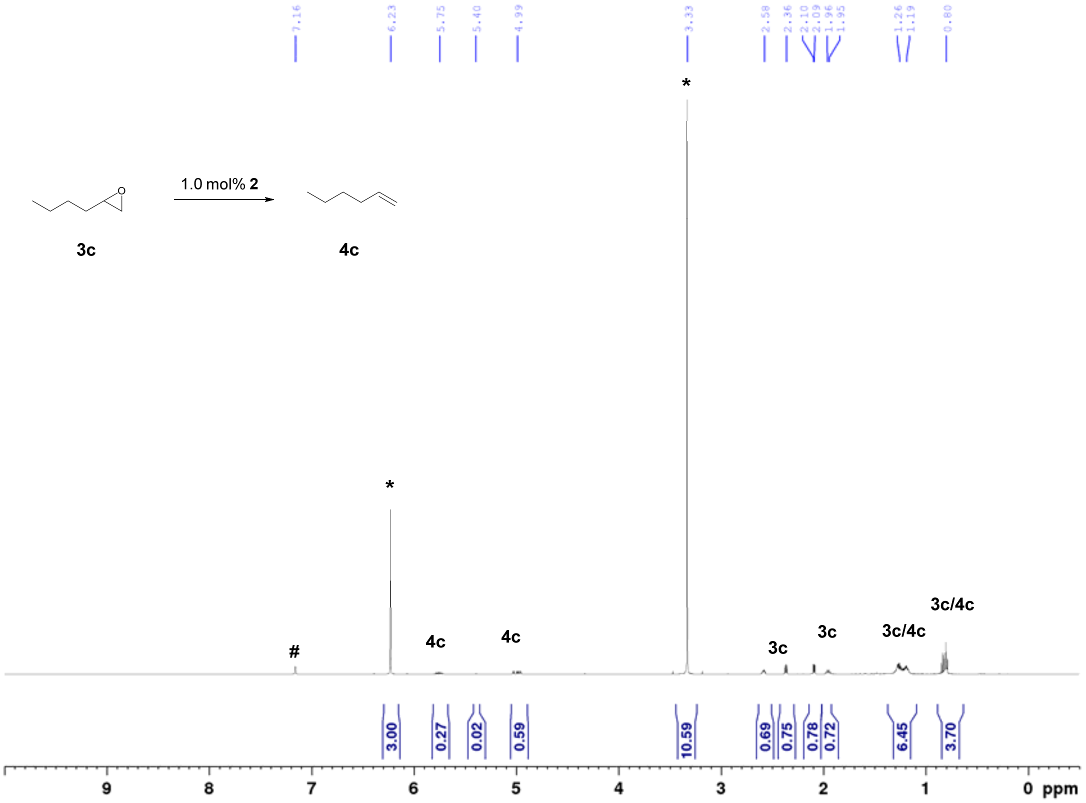

*Figure S25. ^{1}H NMR (C_6D_6 (#), 400 MHz) spectrum: deoxygenation of **3c** into **4c** at 1.0 % catalytic loading; internal standard (*).*

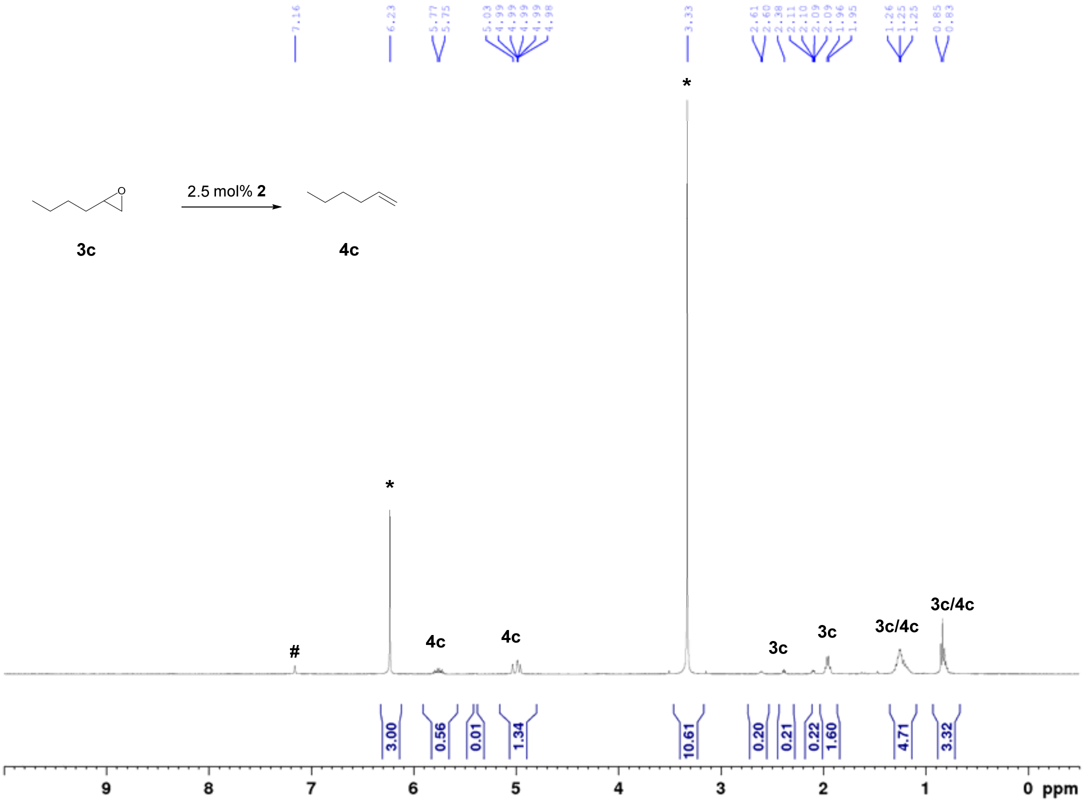

Figure S26. ^{1}H NMR (C_6D_6 (#), 400 MHz) spectrum: deoxygenation of **3c** into **4c** at 2.5 % catalytic loading; internal standard (*).

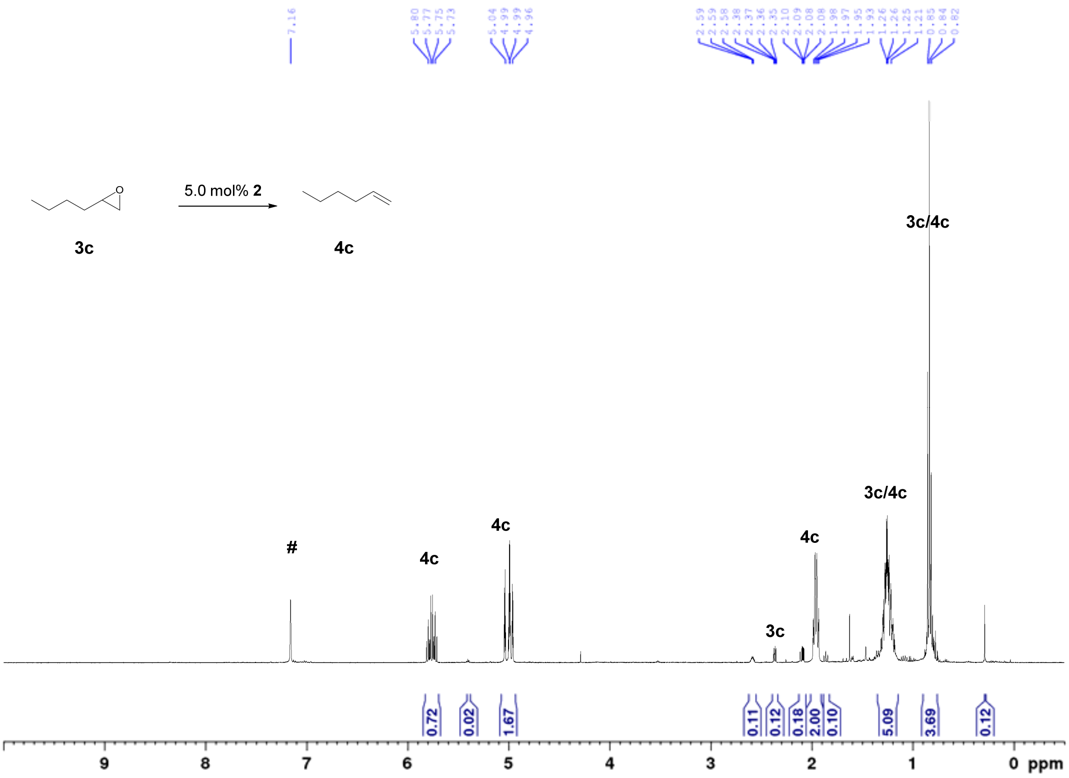

Figure S27. [1]*H NMR (C_6D_6 (#), 400 MHz) spectrum: deoxygenation of* ***3c*** *into* ***4c*** *at 5.0 % catalytic loading; internal standard (*).*

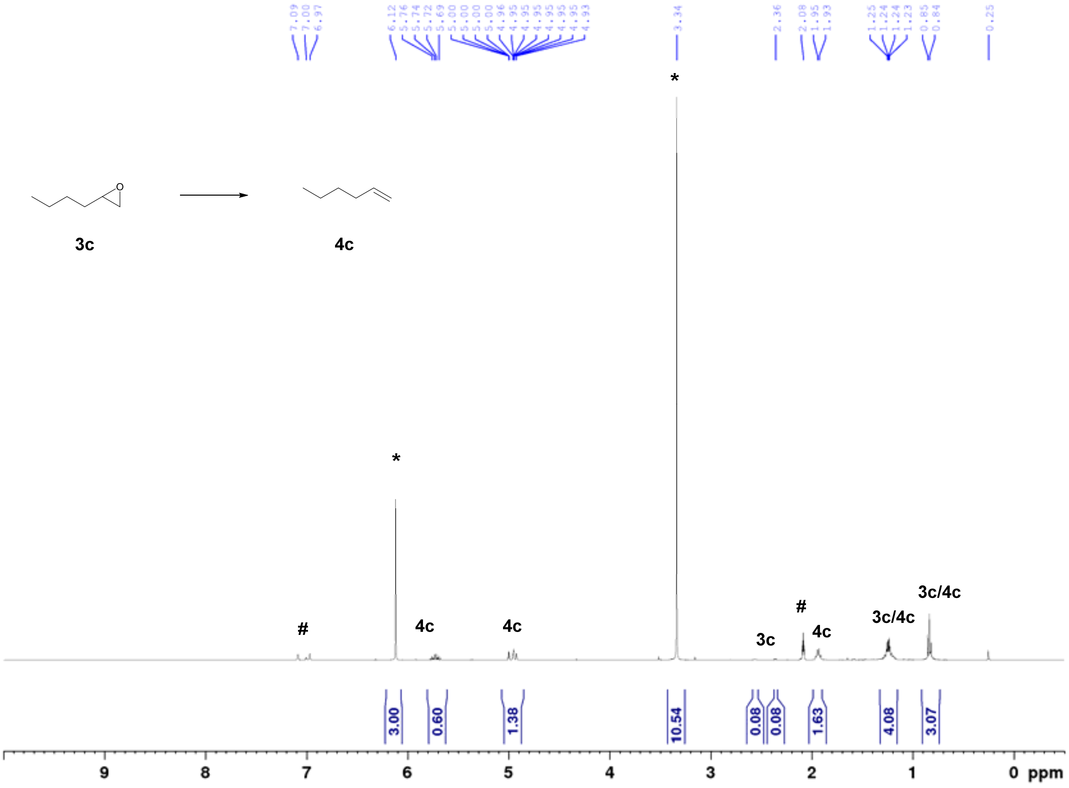

Figure S28. [1]*H NMR (tol-d_8 (#), 400 MHz) spectrum: deoxygenation of* ***3c*** *into* ***4c*** *in toluene as a solvent; internal standard (*).*

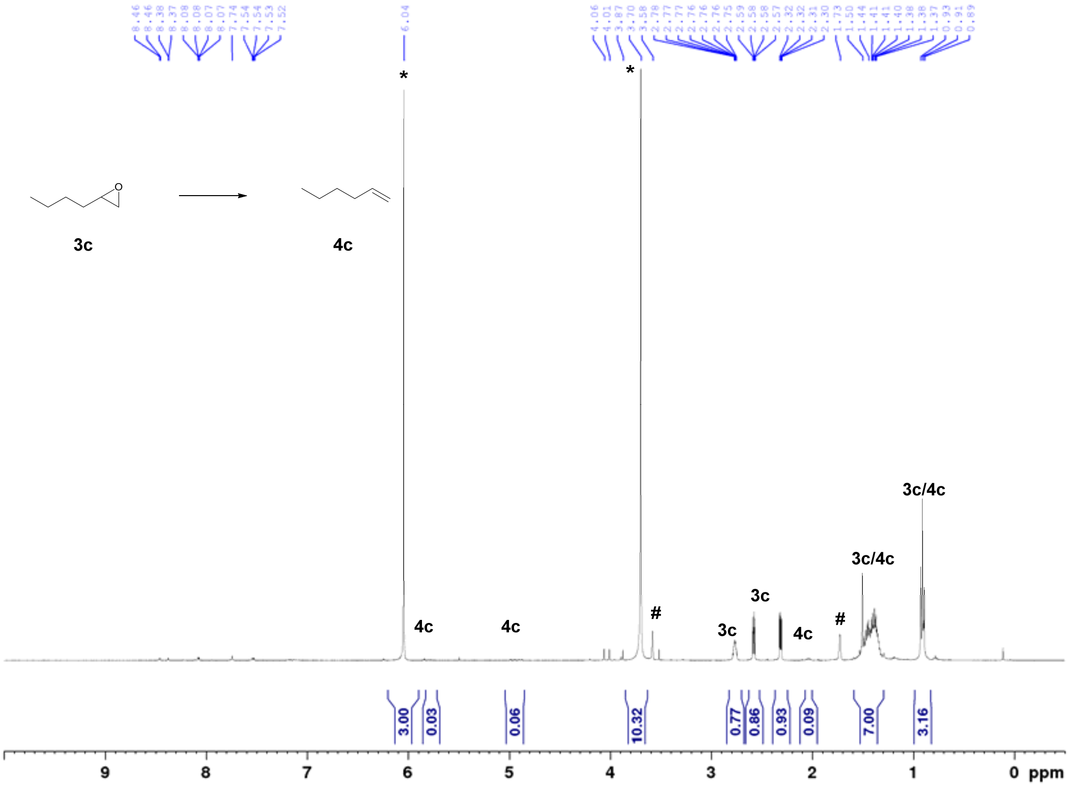

*Figure S29. ^{1}H NMR (thf-d_8 (#), 400 MHz) spectrum: deoxygenation of **3c** into **4c** in THF as a solvent; internal standard (*).*

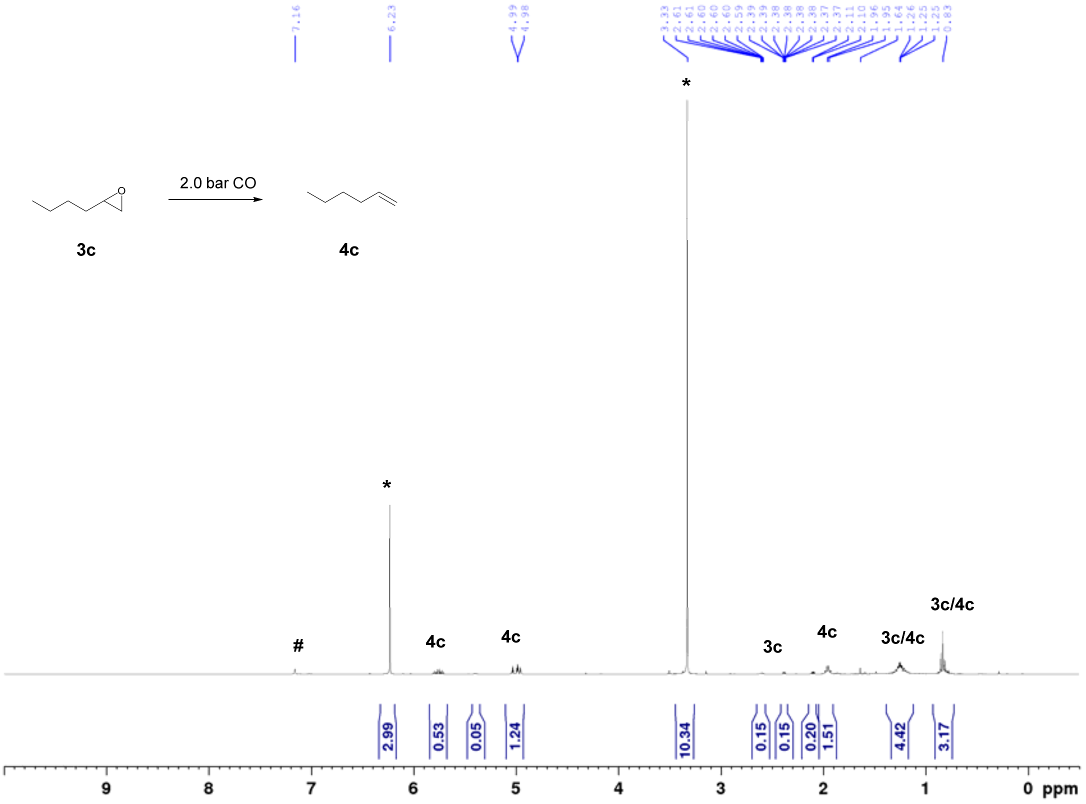

*Figure S30. ^{1}H NMR (C_6D_6 (#), 400 MHz) spectrum: deoxygenation of **3c** into **4c** at 2.0 bar CO pressure; internal standard (*).*

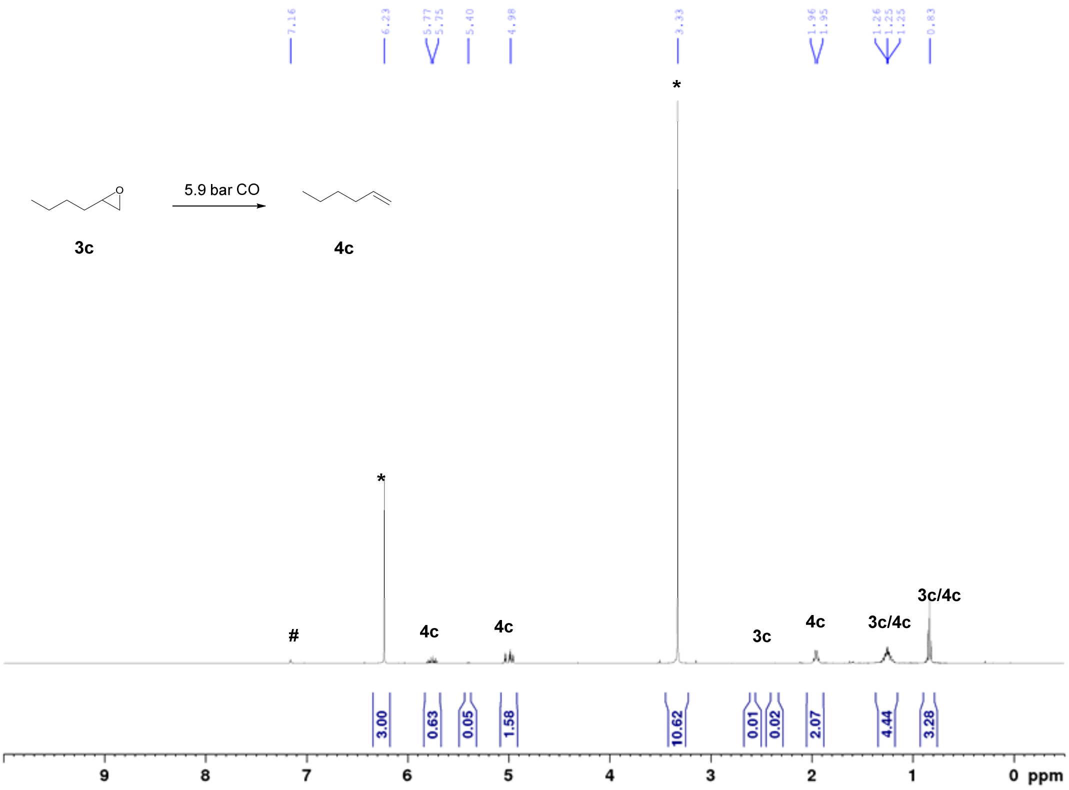

*Figure S31. ^{1}H NMR (C_6D_6 (#), 400 MHz) spectrum: deoxygenation of **3c** into **4c** at 5.9 bar CO pressure; internal standard (*).*

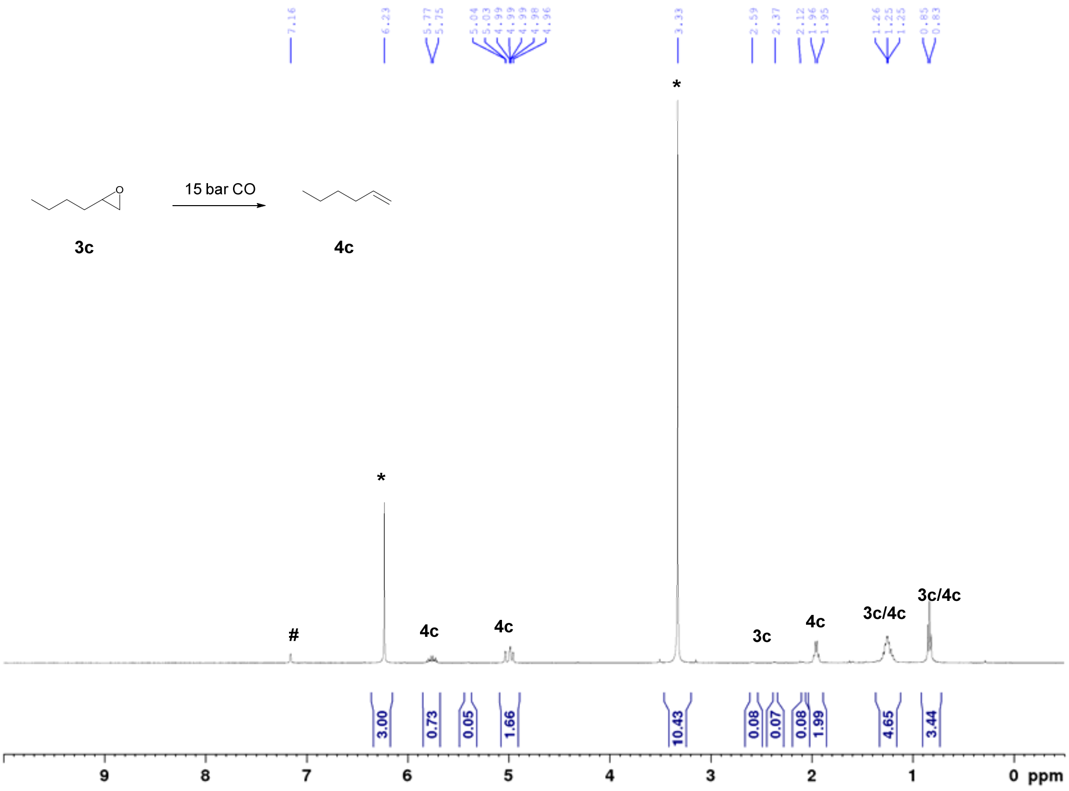

*Figure S32. ^{1}H NMR (C_6D_6 (#), 400 MHz) spectrum: deoxygenation of **3c** into **4c** at 15.4 bar CO pressure; internal standard (*).*

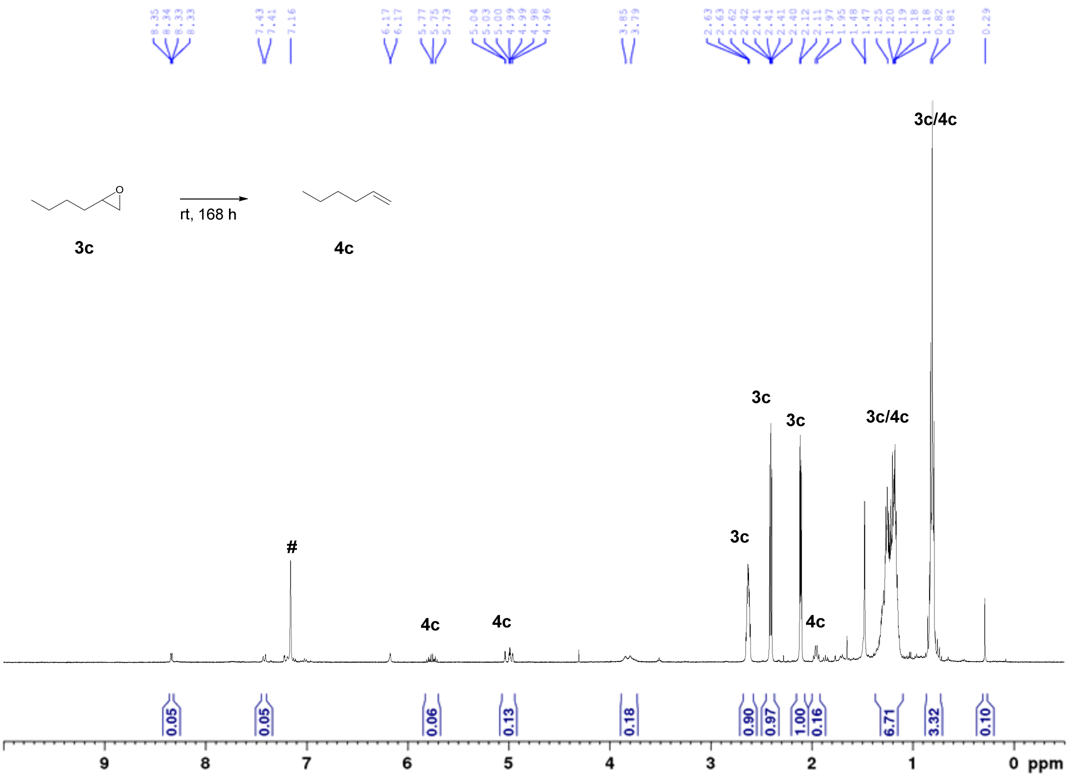

*Figure S33. ^{1}H NMR (C_6D_6 (#), 400 MHz) spectrum: deoxygenation of **3c** into **4c** at room temperature after 168 h; internal standard (*).*

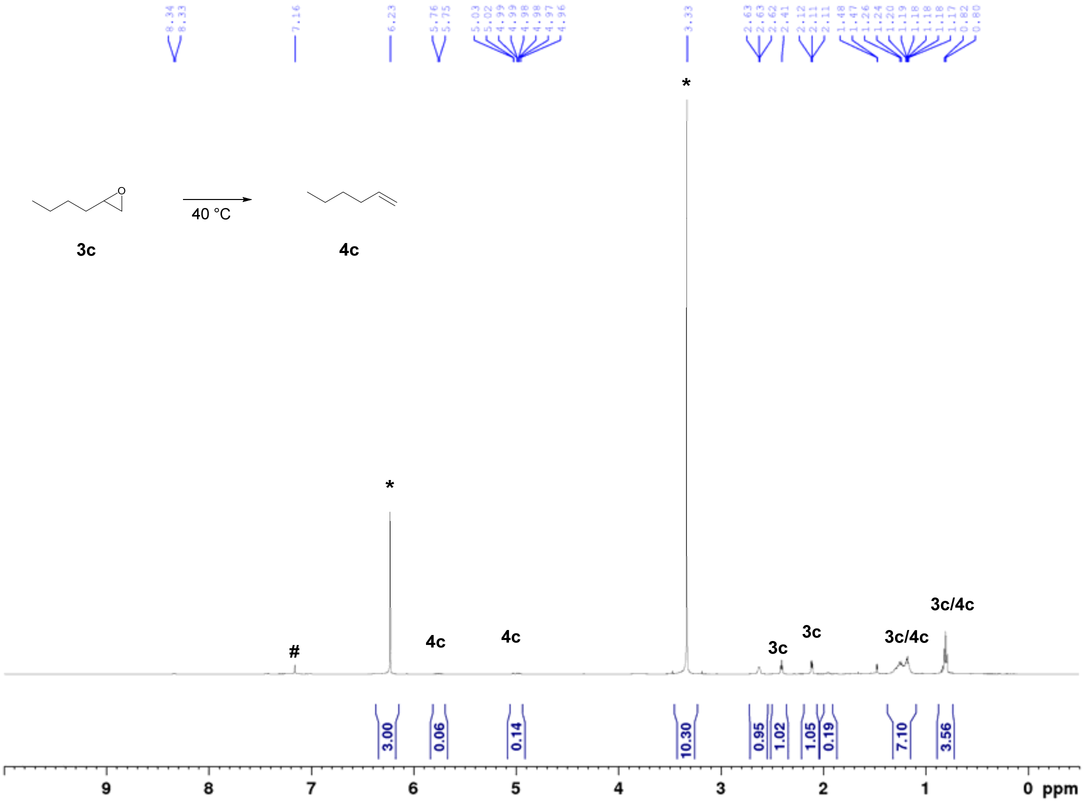

*Figure S34. ¹H NMR (C_6D_6 (#), 400 MHz) spectrum: deoxygenation of **3c** into **4c** at 40 °C; internal standard (*).*

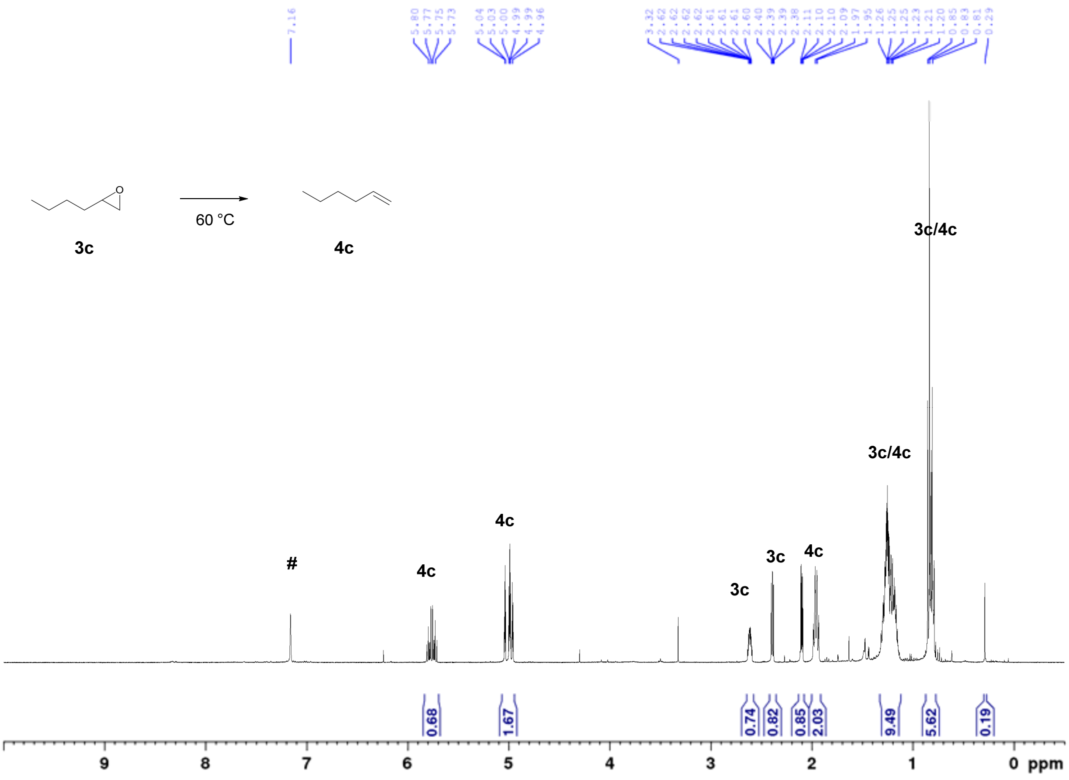

*Figure S35. ^{1}H NMR (C_6D_6 (#), 400 MHz) spectrum: deoxygenation of **3c** into **4c** at 60 °C; internal standard (*).*

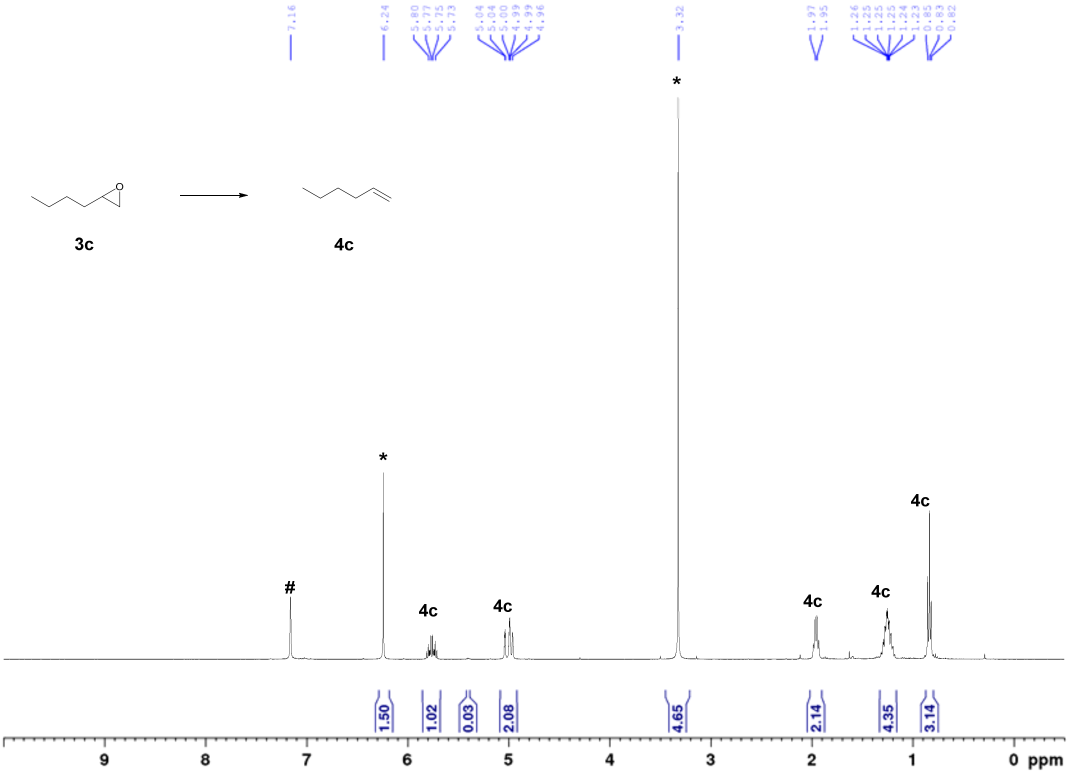

*Figure S36. ^{1}H NMR (C_6D_6 (#), 400 MHz) spectrum: deoxygenation of **3c** into **4c**; internal standard (*).*

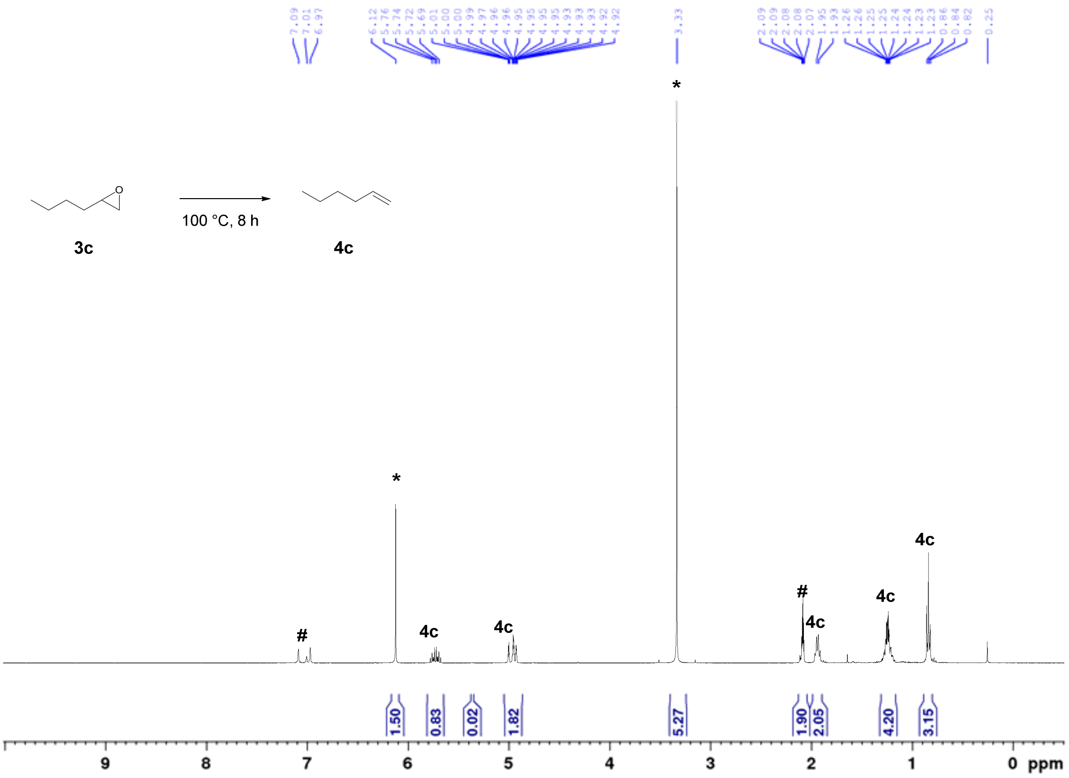

*Figure S37. ^{1}H NMR (toluene-d_8 (#), 400 MHz) spectrum: deoxygenation of **3c** into **4c** at 100 °C after 8 h; internal standard (*).*

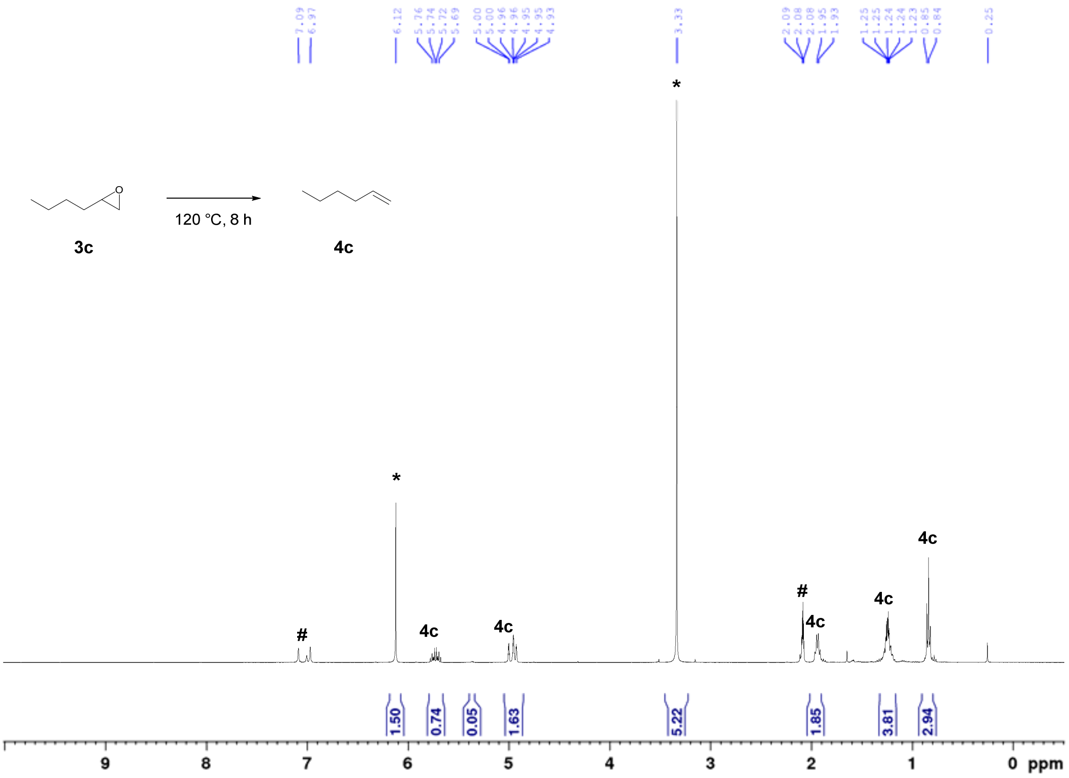

*Figure S38. ^{1}H NMR (toluene-d_8 (#), 400 MHz) spectrum: deoxygenation of **3c** into **4c** at 120 °C after 8 h; internal standard (*).*

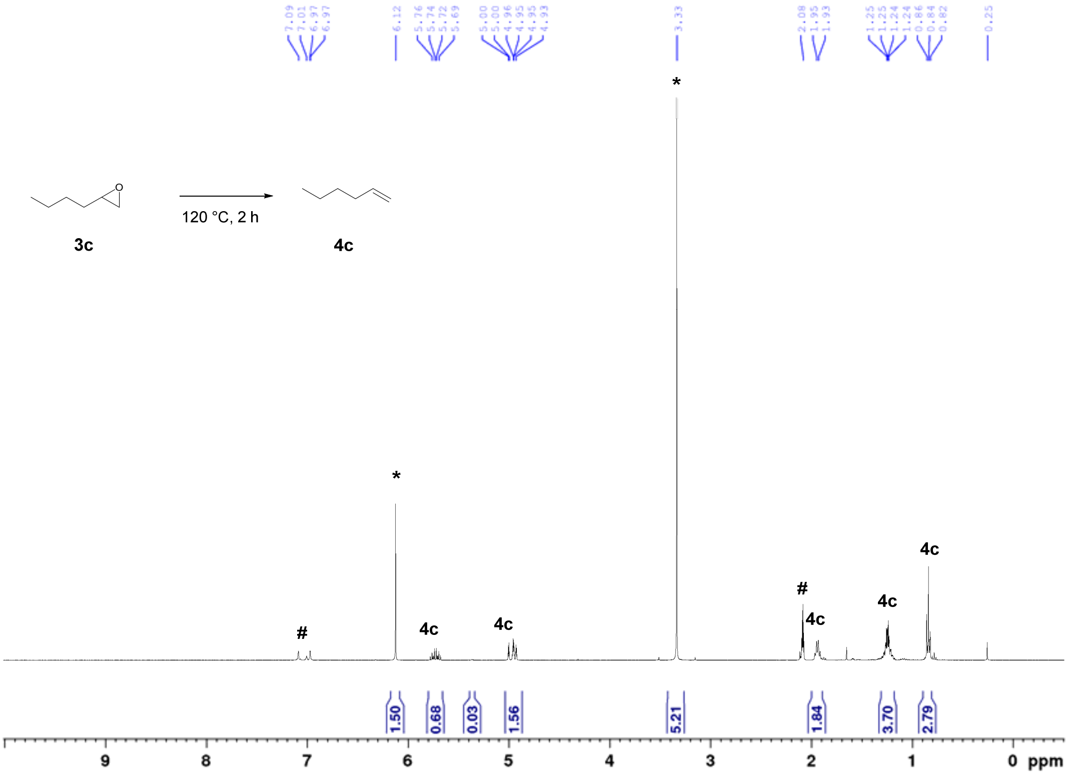

*Figure S39. ^{1}H NMR (toluene-d_8 (#), 400 MHz) spectrum: deoxygenation of **3c** into **4c** at 120 °C after 2 h; internal standard (*).*

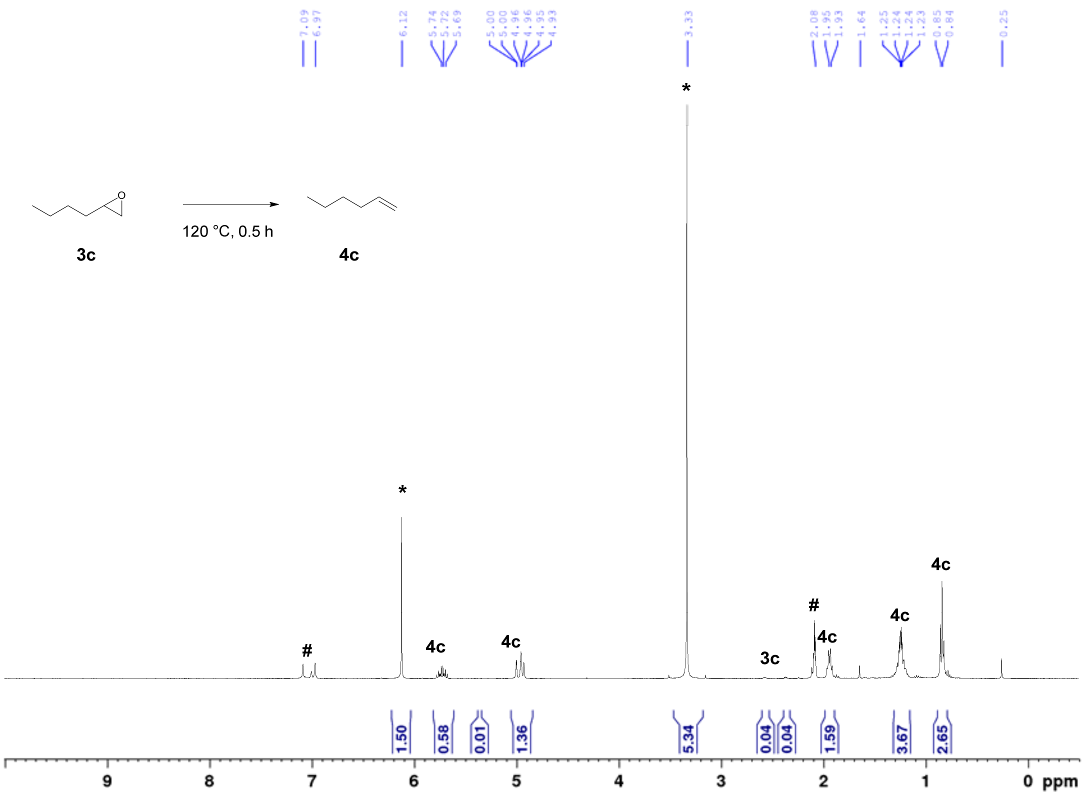

*Figure S40. ¹H NMR (toluene-d$_8$ (#), 400 MHz) spectrum: deoxygenation of **3c** into **4c** at 120 °C after 0.5 h; internal standard (*).*

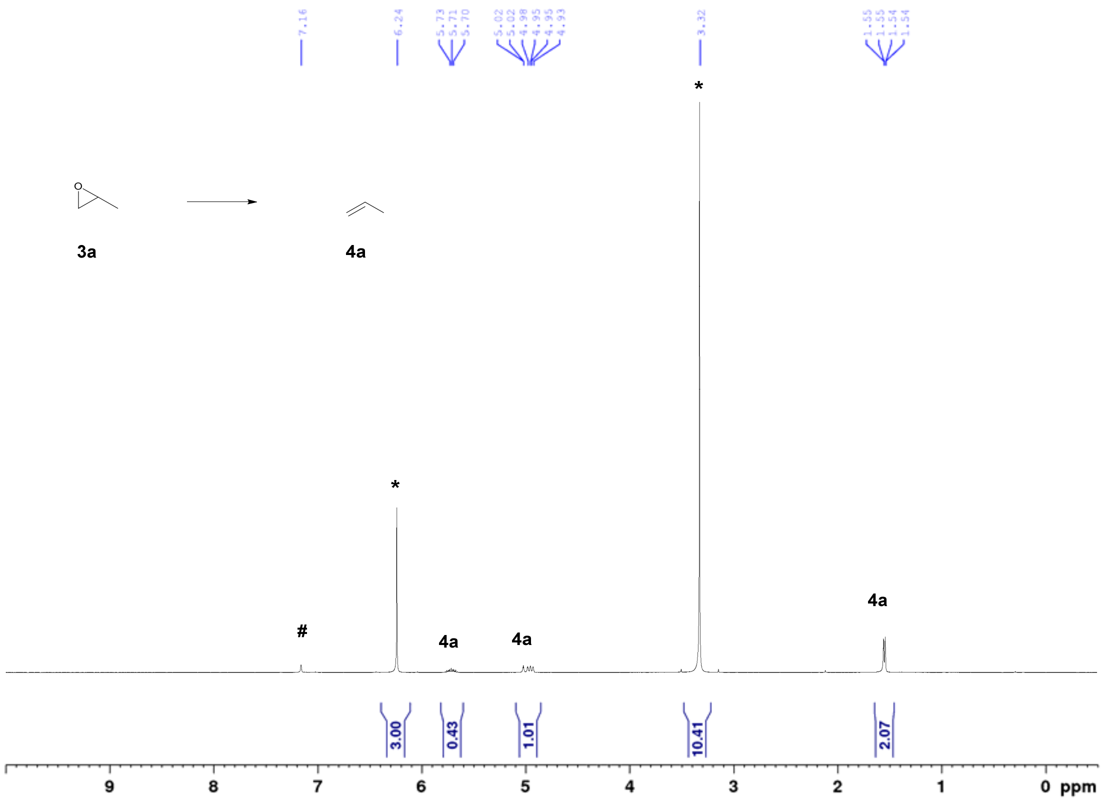

*Figure S41. ¹H NMR (C_6D_6 (#), 400 MHz) spectrum: deoxygenation of **3a** into **4a**; internal standard (*).*

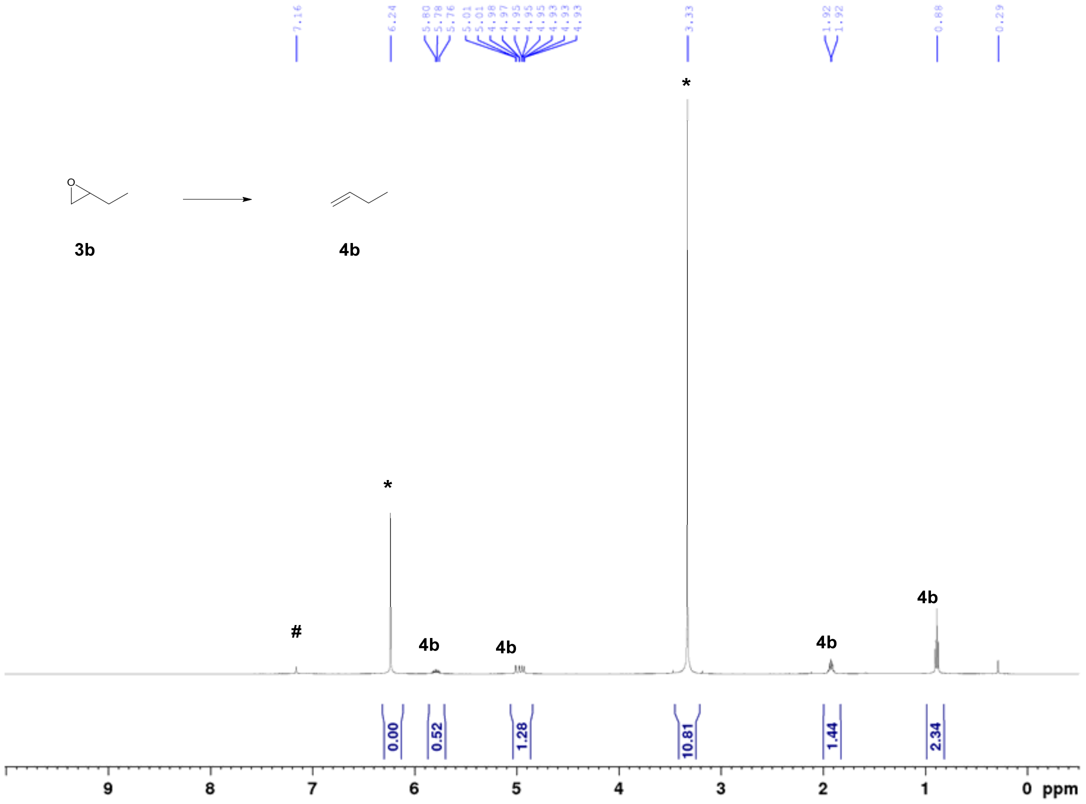

*Figure S42. ^{1}H NMR (C_6D_6 (#), 400 MHz) spectrum: deoxygenation of **3b** into **4b**; internal standard (*).*

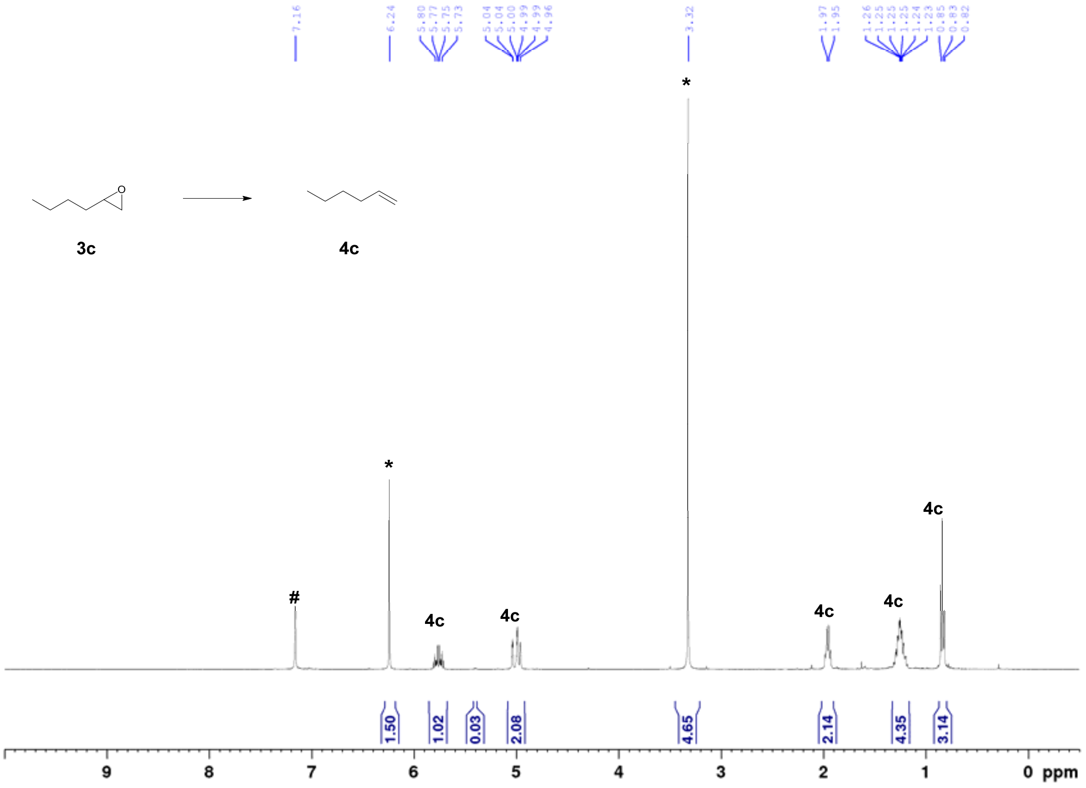

*Figure S43. ^{1}H NMR (C_6D_6 (#), 400 MHz) spectrum: deoxygenation of **3c** into **4c**; internal standard (*).*

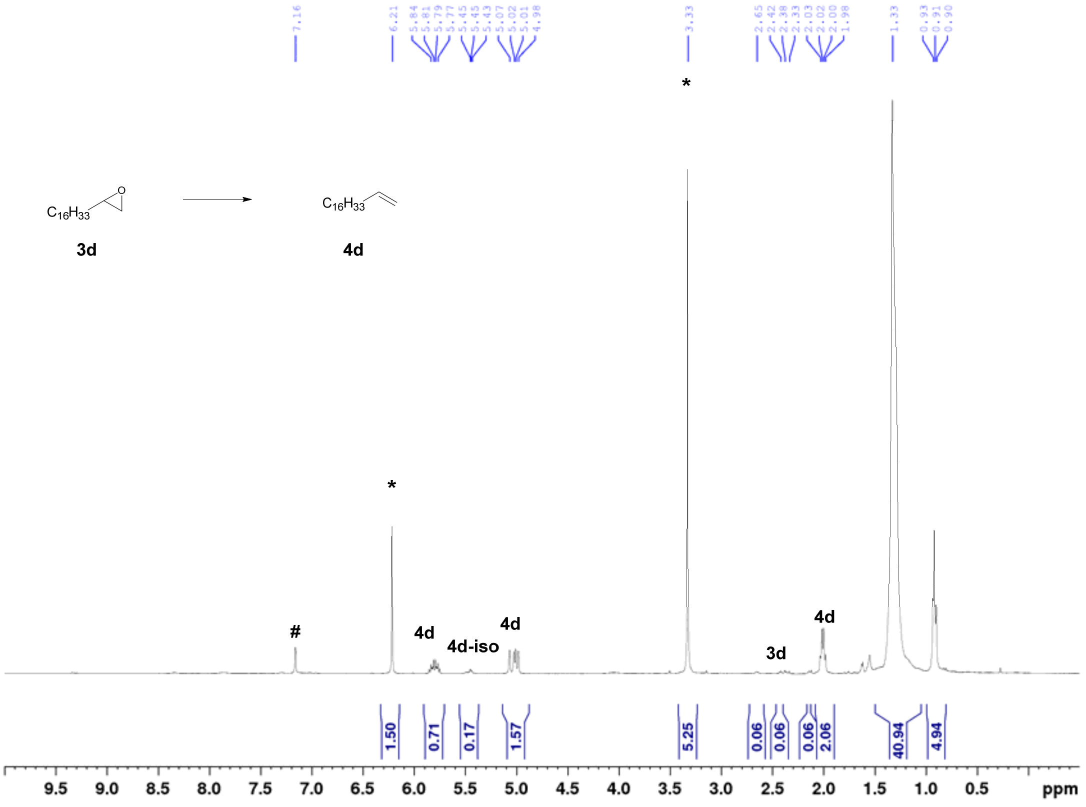

*Figure S44. ^1H NMR (C_6D_6 (#), 400 MHz) spectrum: deoxygenation of **3d** into **4d**; internal standard (*).*

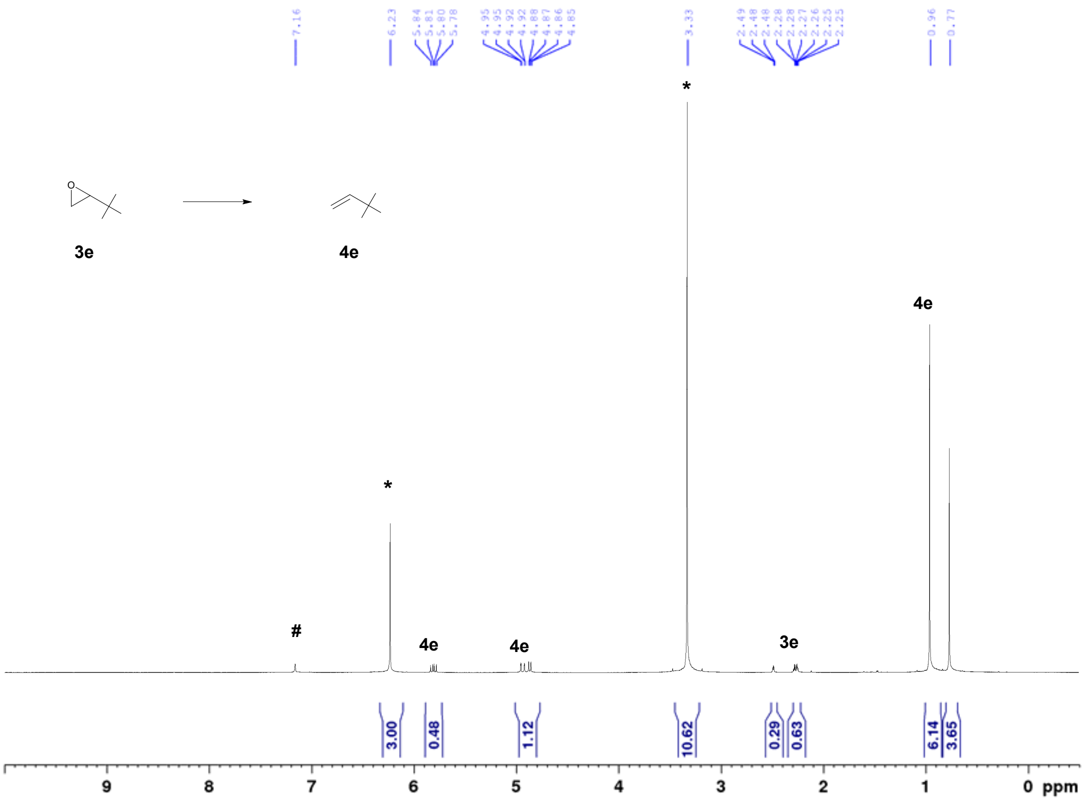

*Figure S45. ^1H NMR (C_6D_6 (#), 400 MHz) spectrum: deoxygenation of **3e** into **4e**; internal standard (*).*

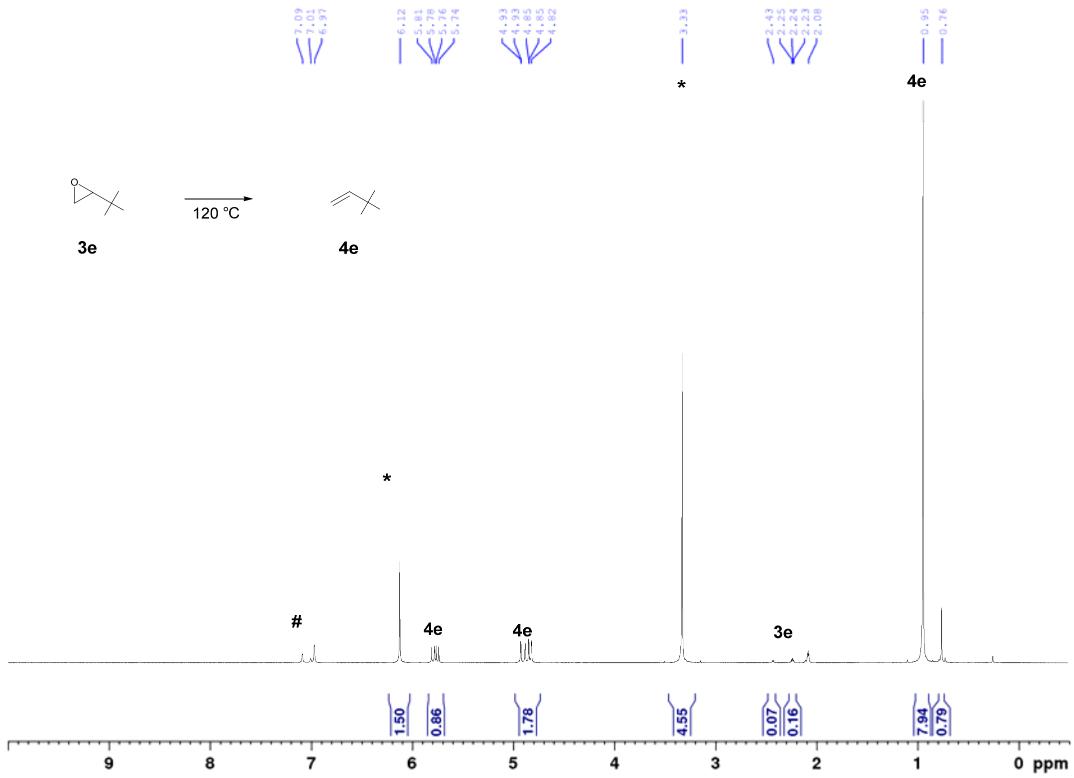

*Figure S46. ^{1}H NMR (toluene-d_8 (#), 400 MHz) spectrum: deoxygenation of **3e** into **4e** at 120 °C; internal standard (*).*

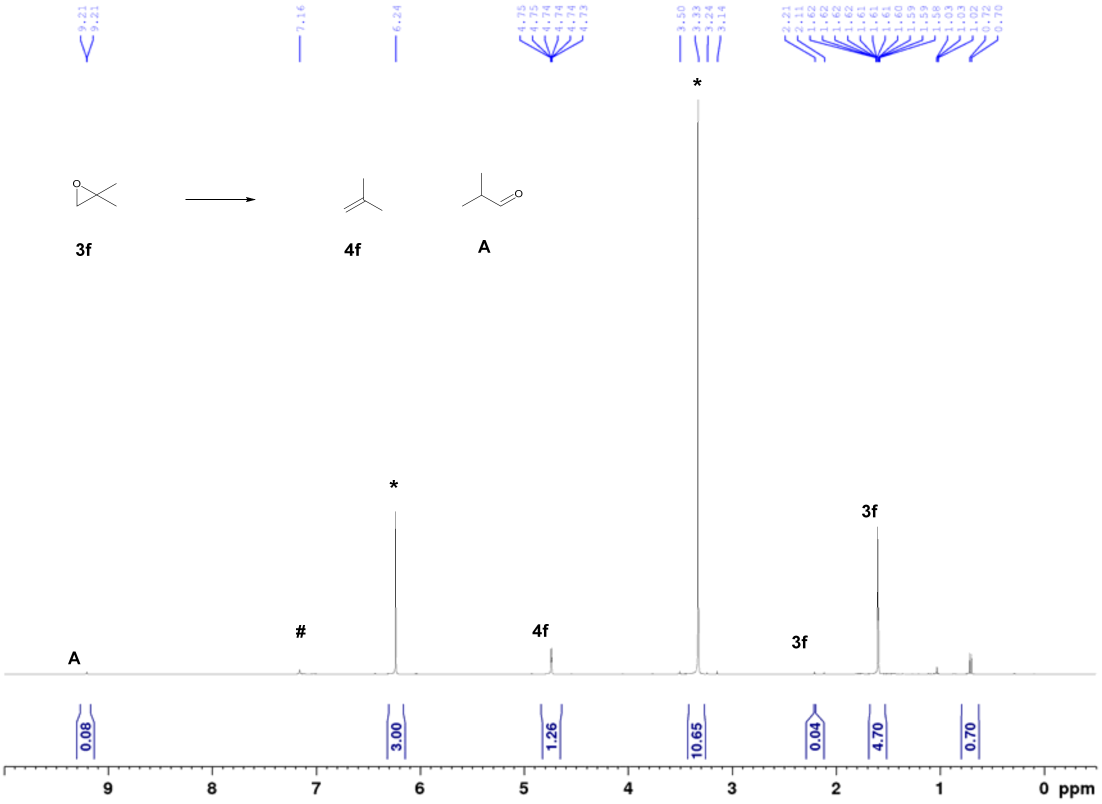

Figure S47. *^{1}H NMR (C_6D_6 (#), 400 MHz) spectrum: deoxygenation of* ***3f*** *into* ***4f****; internal standard (*).*

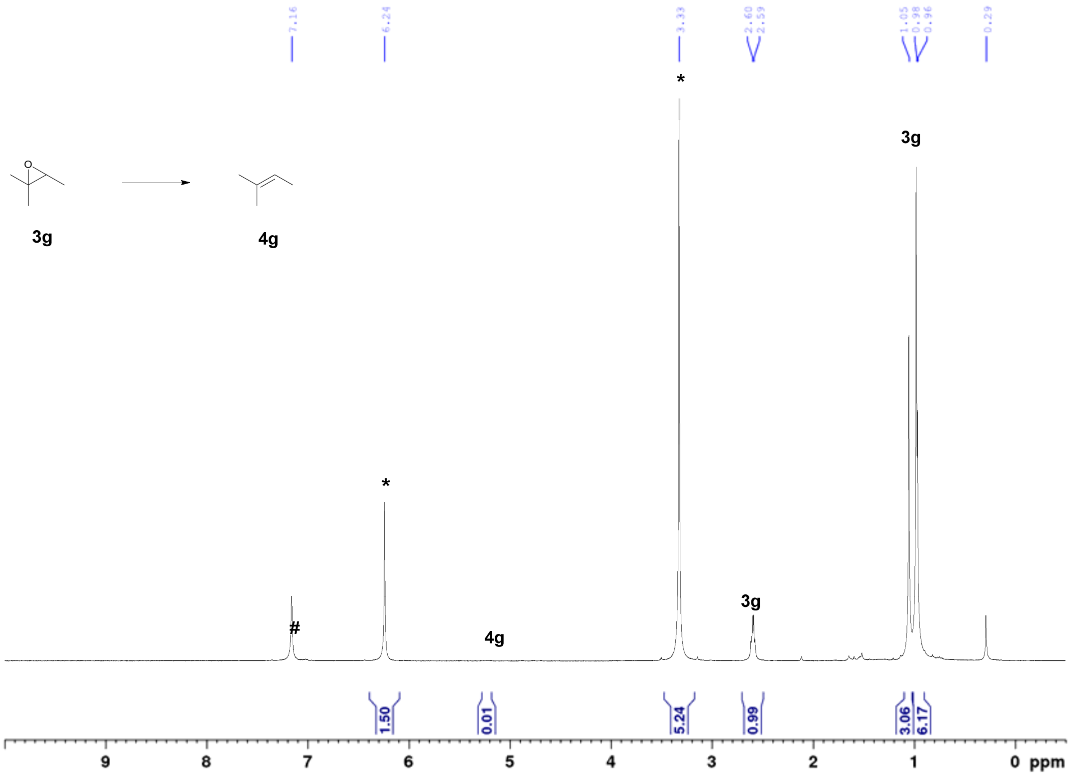

*Figure S48. ¹H NMR (C_6D_6 (#), 400 MHz) spectrum: deoxygenation of **3g** into **4g**; internal standard (*).*

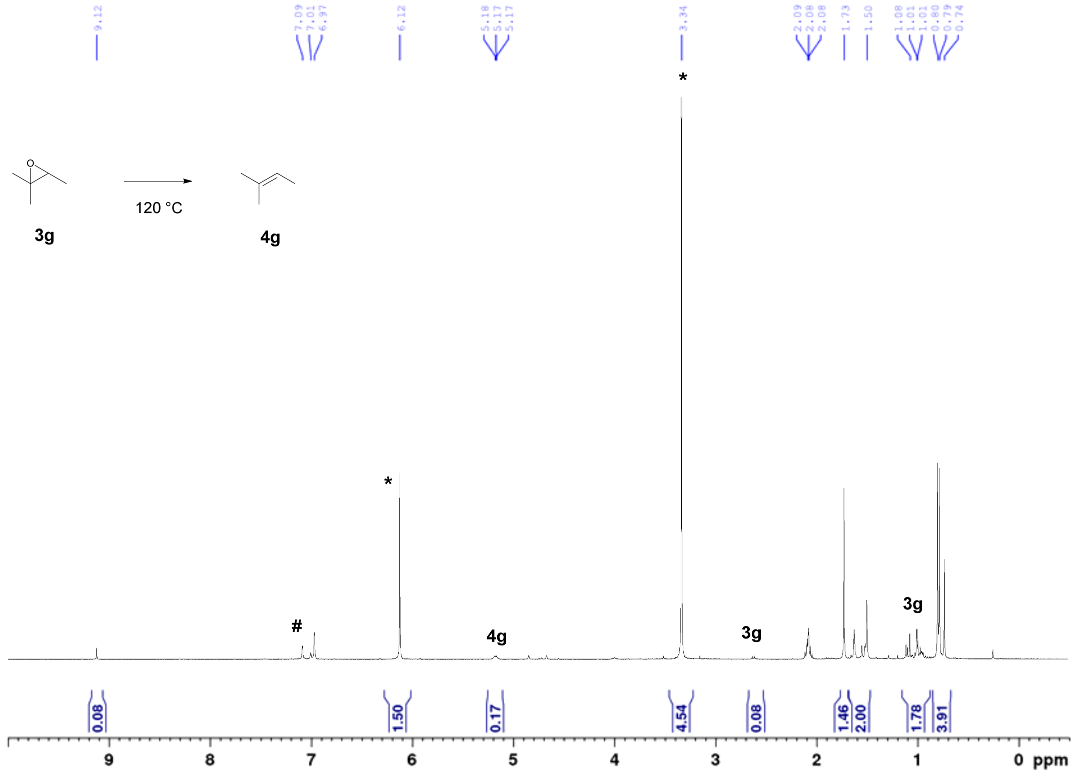

*Figure S49. ¹H NMR (toluene-d_8 (#), 400 MHz) spectrum: deoxygenation of **3g** into **4g** at 120 °C; internal standard (*).*

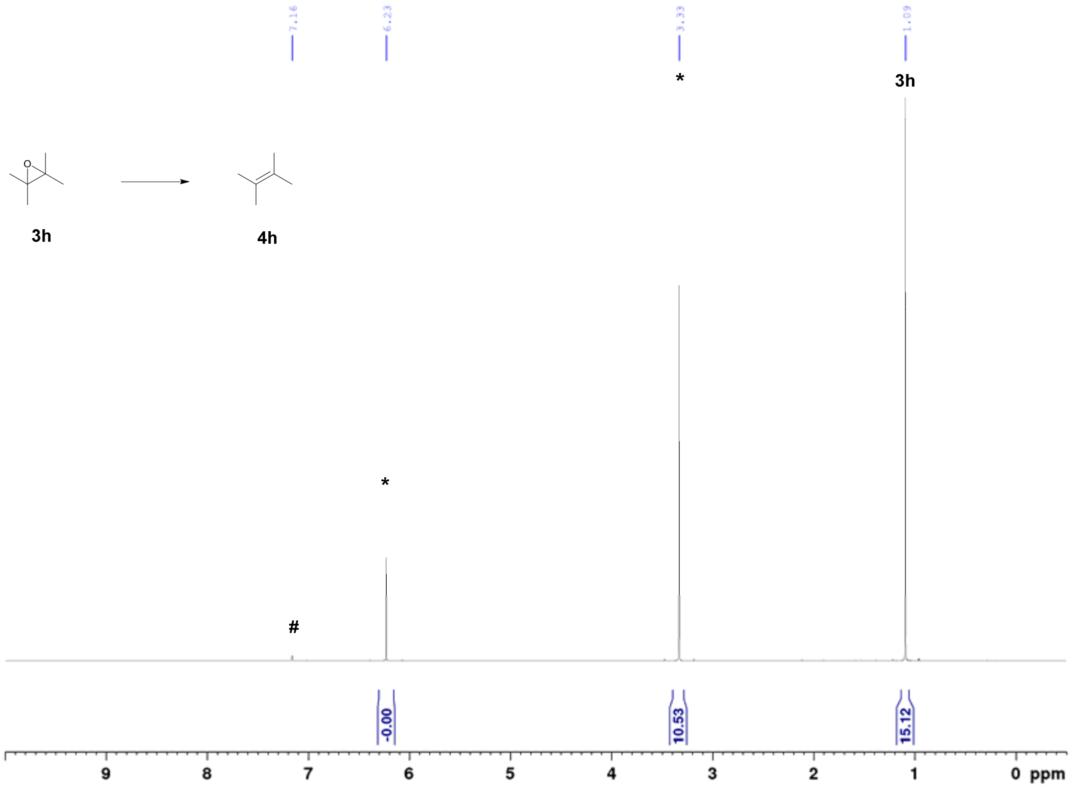

*Figure S50. ^{1}H NMR (C_6D_6 (#), 400 MHz) spectrum: deoxygenation of **3h** into **4h**; internal standard (*).*

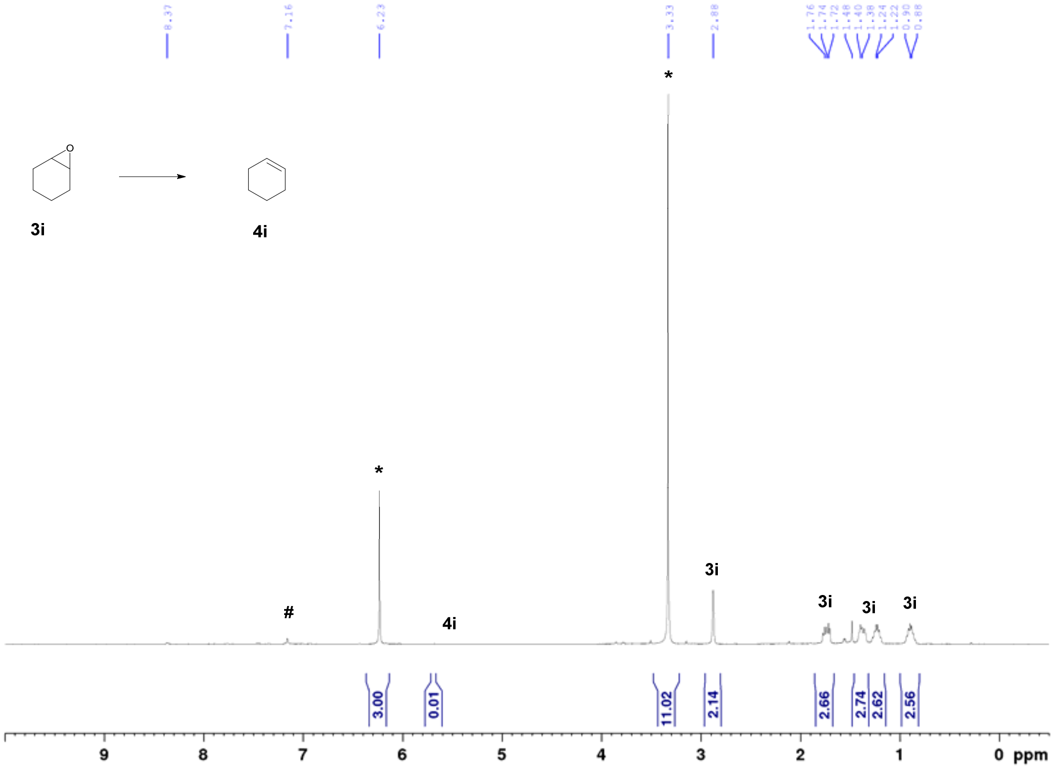

*Figure S51. ^{1}H NMR (C_6D_6 (#), 400 MHz) spectrum: deoxygenation of **3l** into **4l**; internal standard (*).*

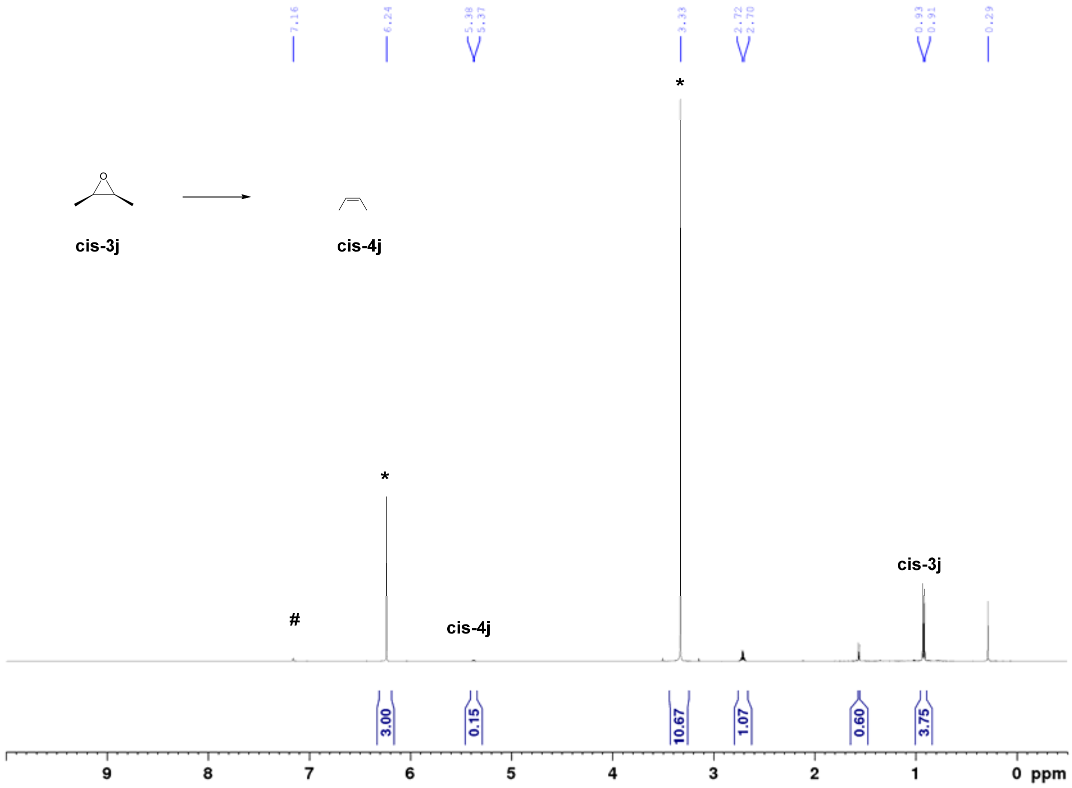

Figure S52. ¹H NMR (C_6D_6 (#), 400 MHz) spectrum: deoxygenation of ***cis-3j*** into ***cis-4j*** after 224 h; internal standard (*).

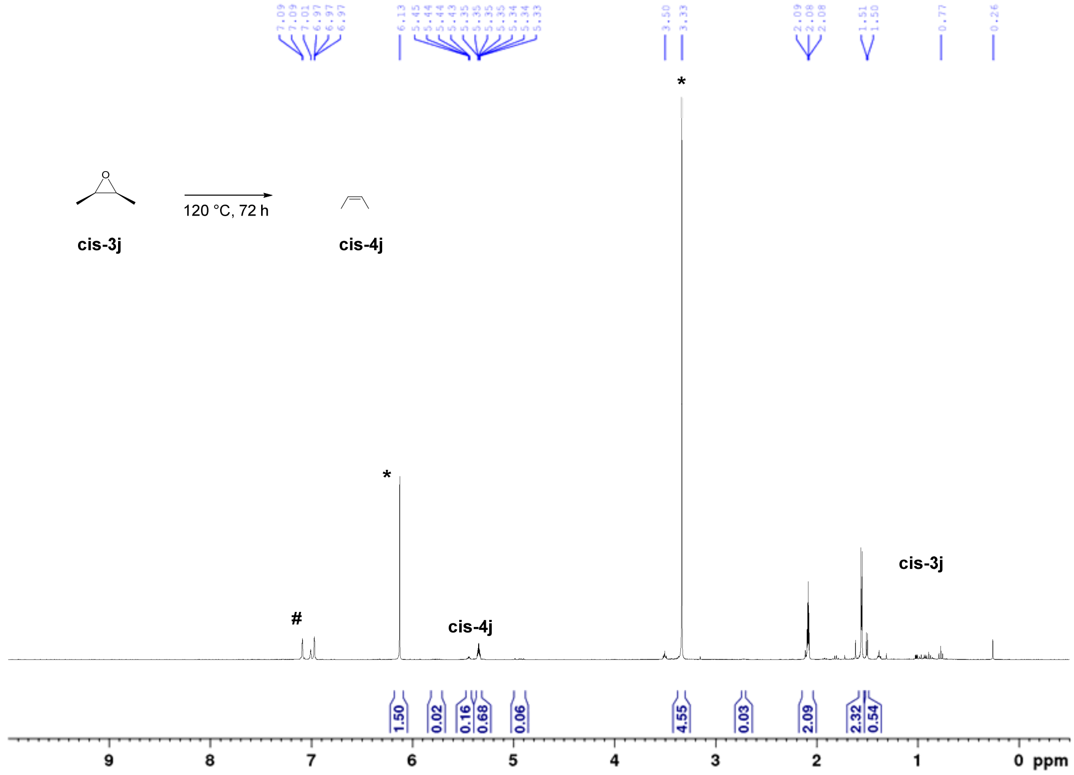

Figure S53. ¹H NMR (toluene-d_8 (#), 400 MHz) spectrum: deoxygenation of ***cis-3j*** into ***cis-4j*** at 120 °C after 72 h; internal standard (*).

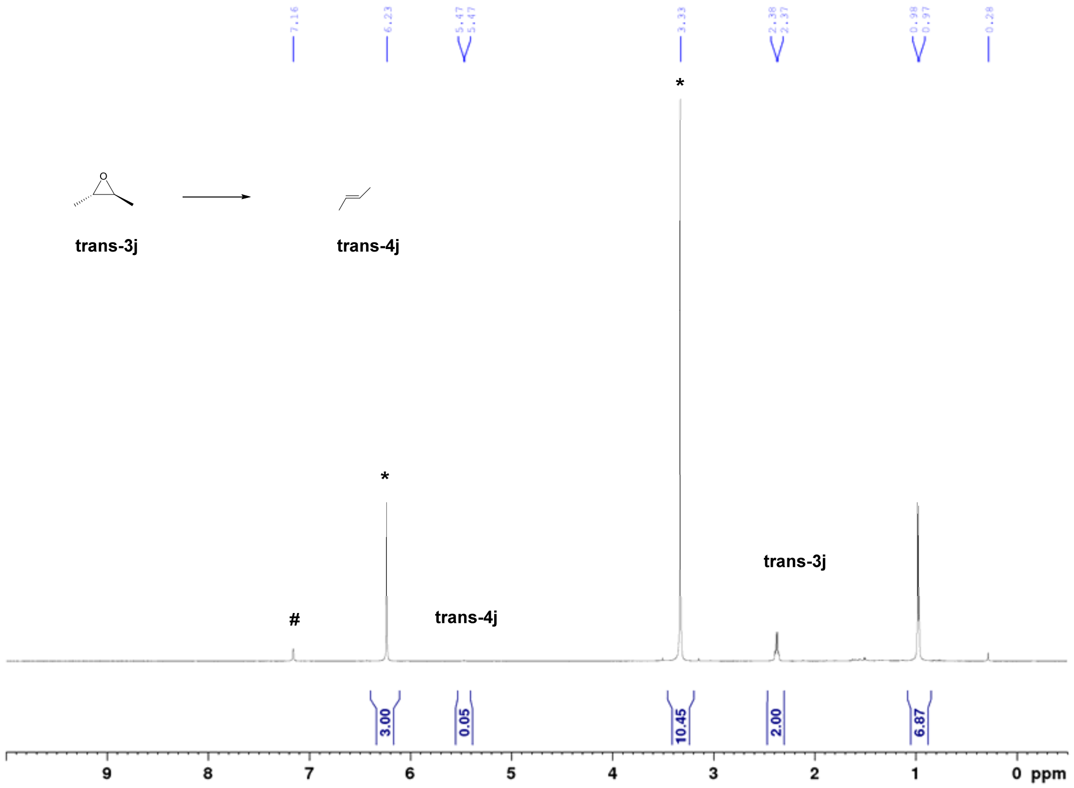

*Figure S54. ¹H NMR (C_6D_6 (#), 400 MHz) spectrum: deoxygenation of **trans-3j** into **trans-4j** after 224 h; internal standard (*).*

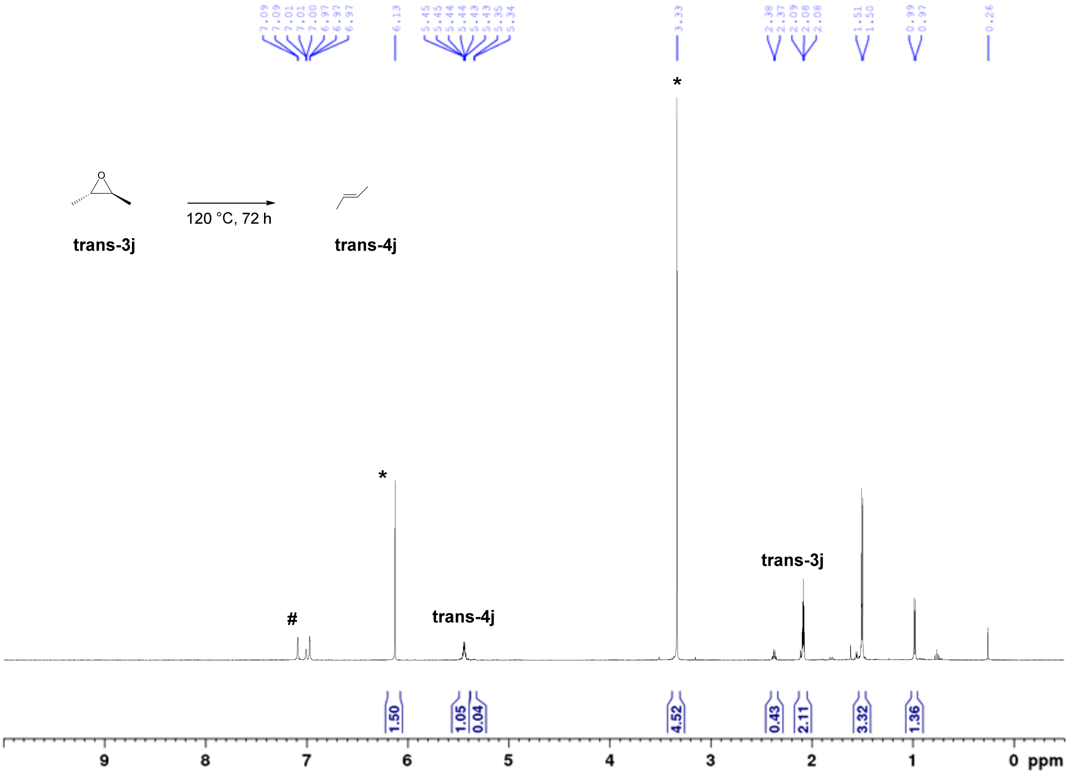

*Figure S55. ^{1}H NMR (toluene-d_8 (#), 400 MHz) spectrum: deoxygenation of **trans-3j** into **trans-4j** at 120 °C after 72 h; internal standard (*).*

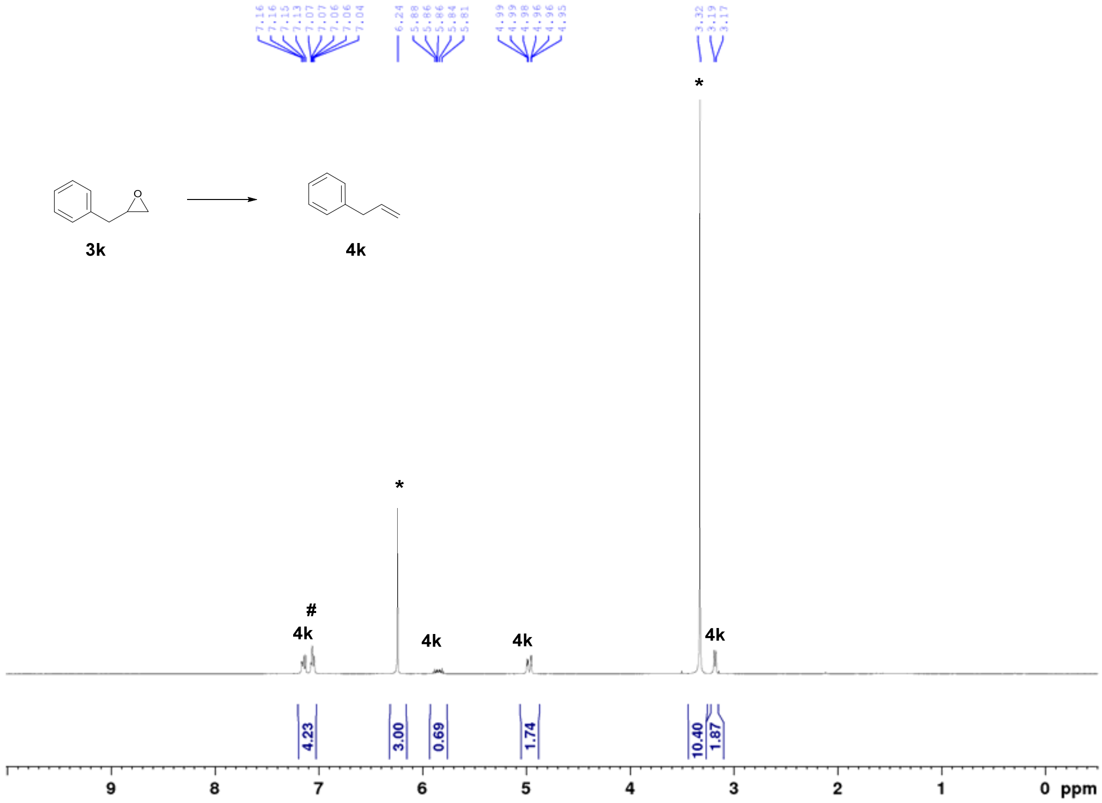

Figure S56. [1]*H NMR (C_6D_6 (#), 400 MHz) spectrum: deoxygenation of* ***3k*** *into* ***4k****; internal standard (*).*

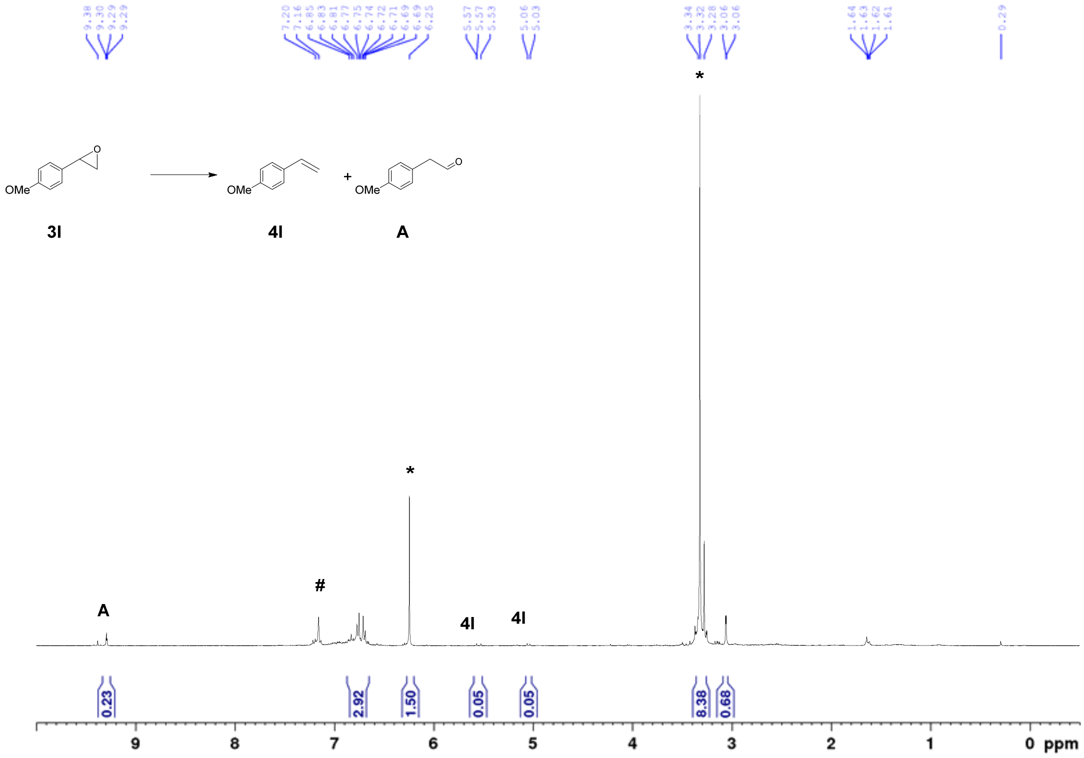

Figure S57. [1]*H NMR (C_6D_6 (#), 400 MHz) spectrum: deoxygenation of* ***3l*** *into* ***4l****; internal standard (*).*

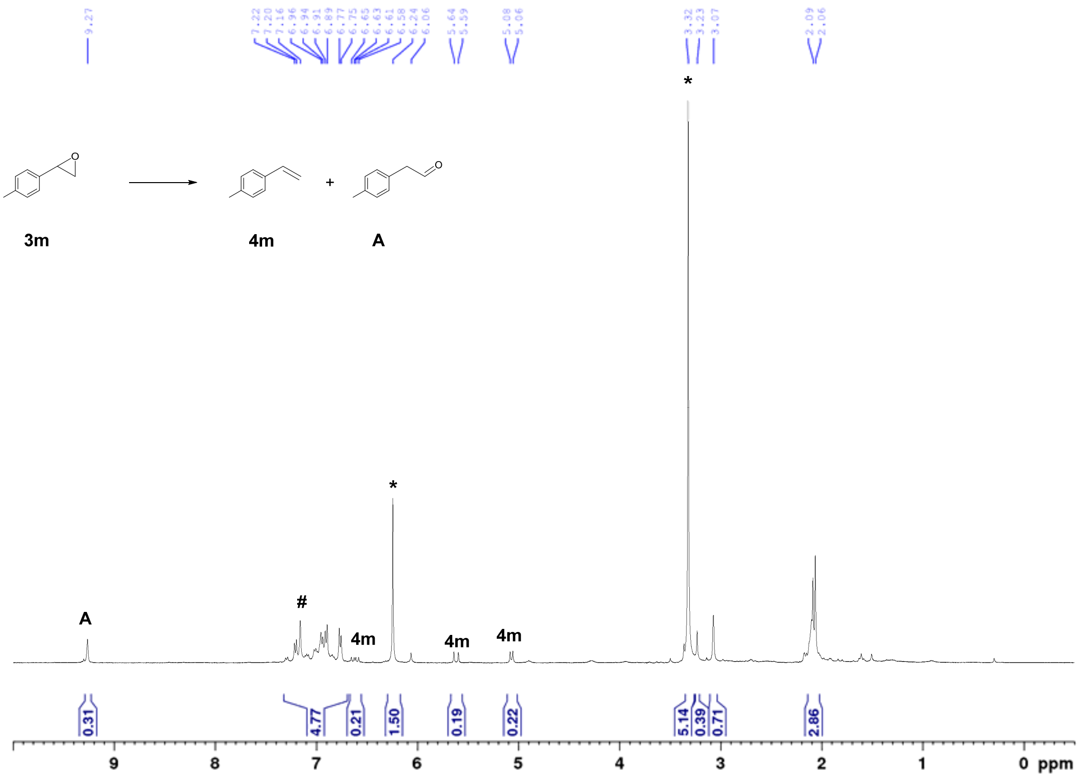

Figure S58. ^{1}H *NMR (C_6D_6 (#), 400 MHz) spectrum: deoxygenation of **3m** into **4m**; internal standard (*).*

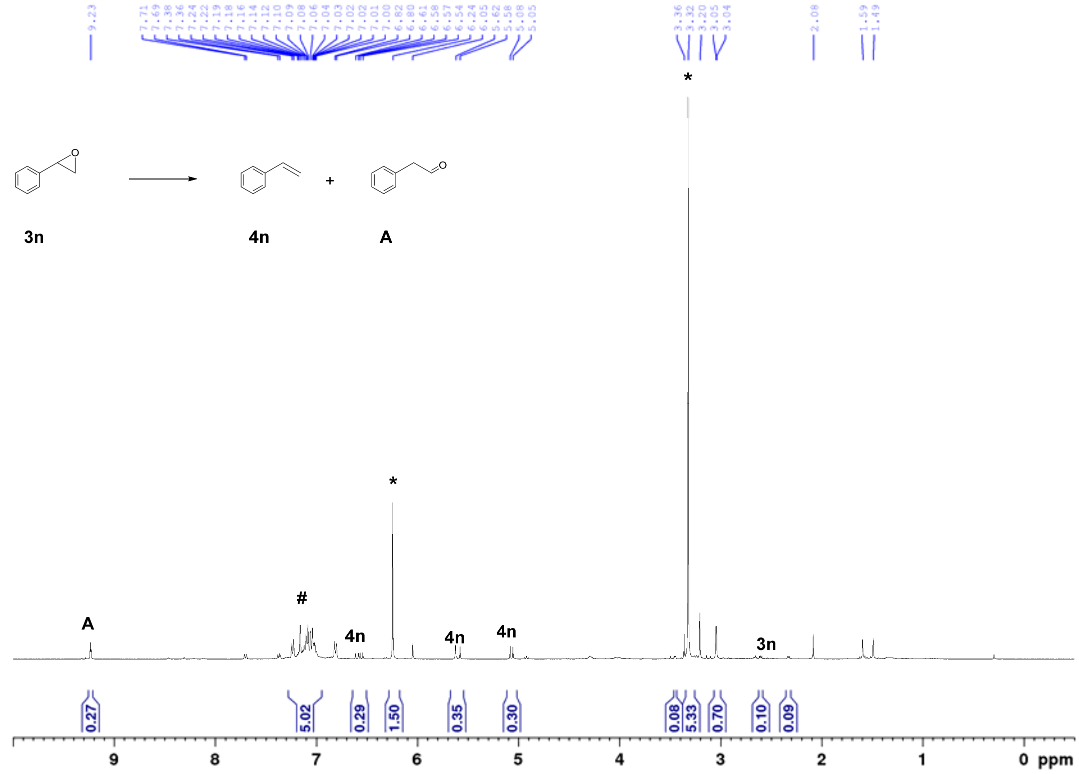

Figure S59. ^{1}H *NMR (C_6D_6 (#), 400 MHz) spectrum: deoxygenation of **3n** into **4n**; internal standard (*).*

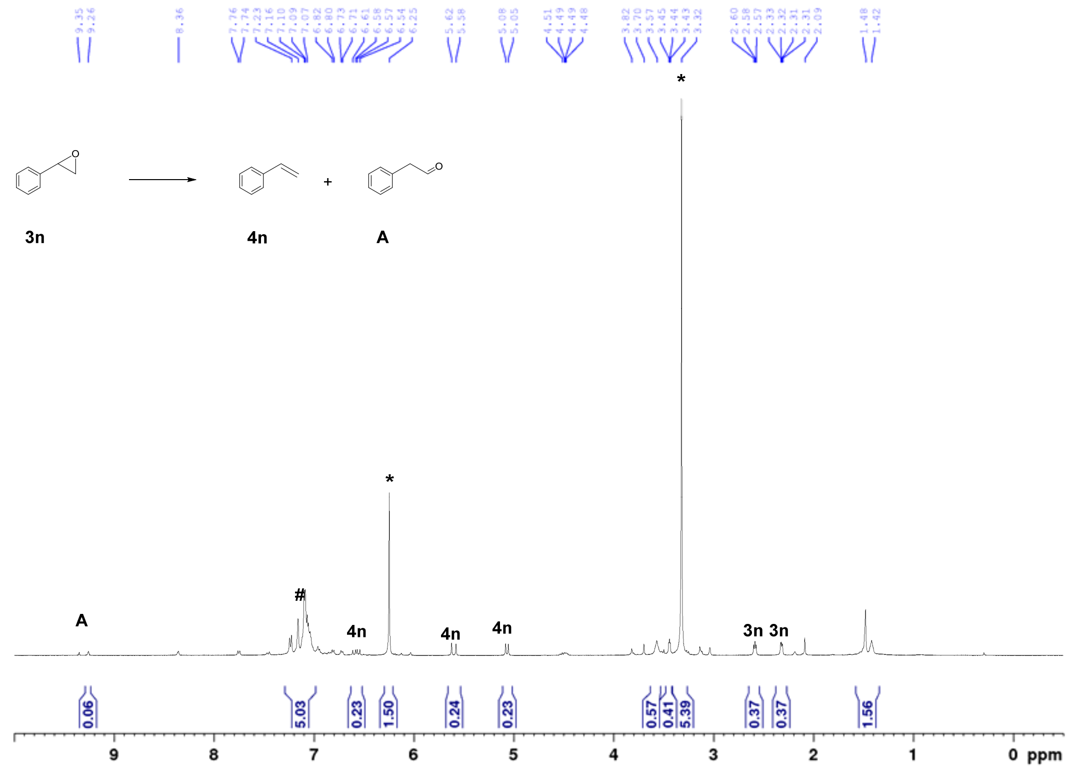

*Figure S60. ^{1}H NMR (C_6D_6 (#), 400 MHz) spectrum: deoxygenation of **3n** into **4n** with LiBr; internal standard (*).*

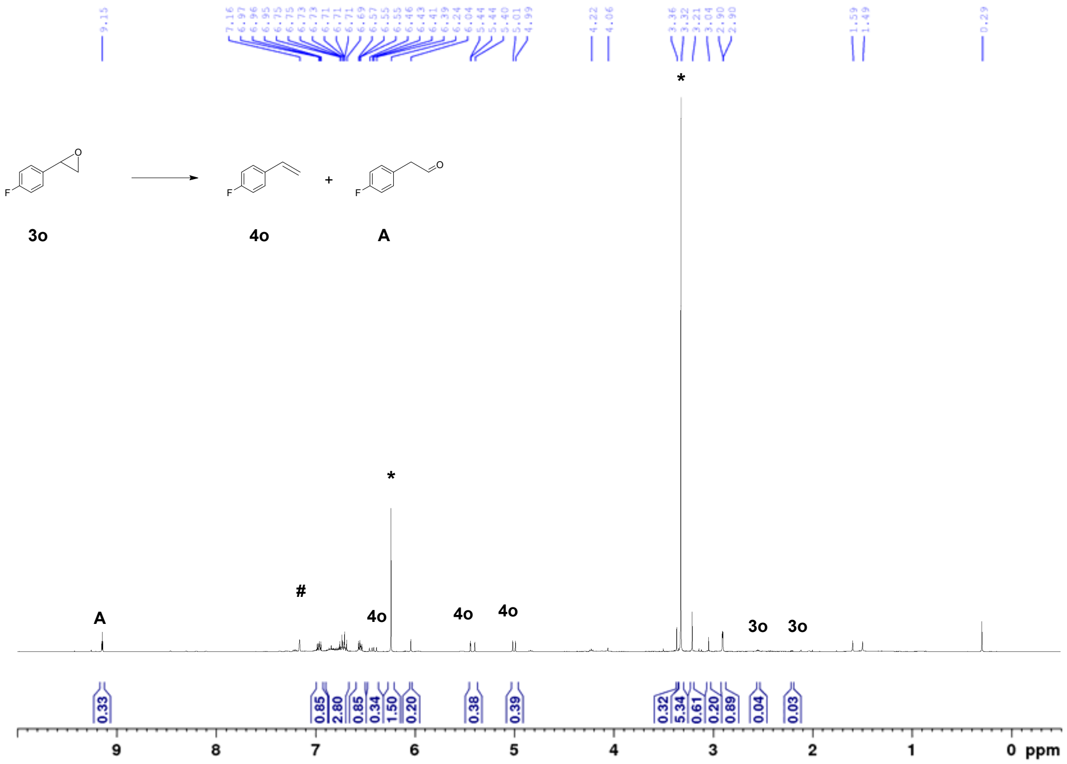

*Figure S61 . ^{1}H NMR (C_6D_6 (#), 400 MHz) spectrum: deoxygenation of **3o** into **4o**; internal standard (*).*

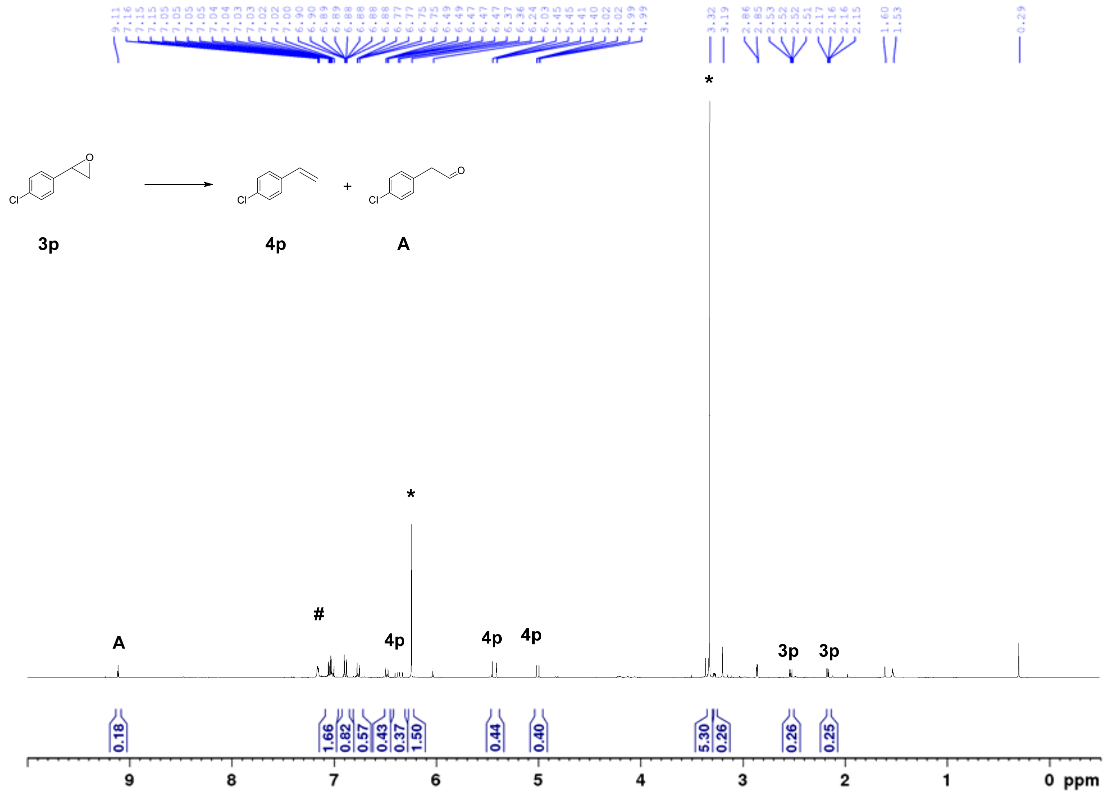

*Figure S62. ^{1}H NMR (C_6D_6 (#), 400 MHz) spectrum: deoxygenation of **3p** into **4p**; internal standard (*).*

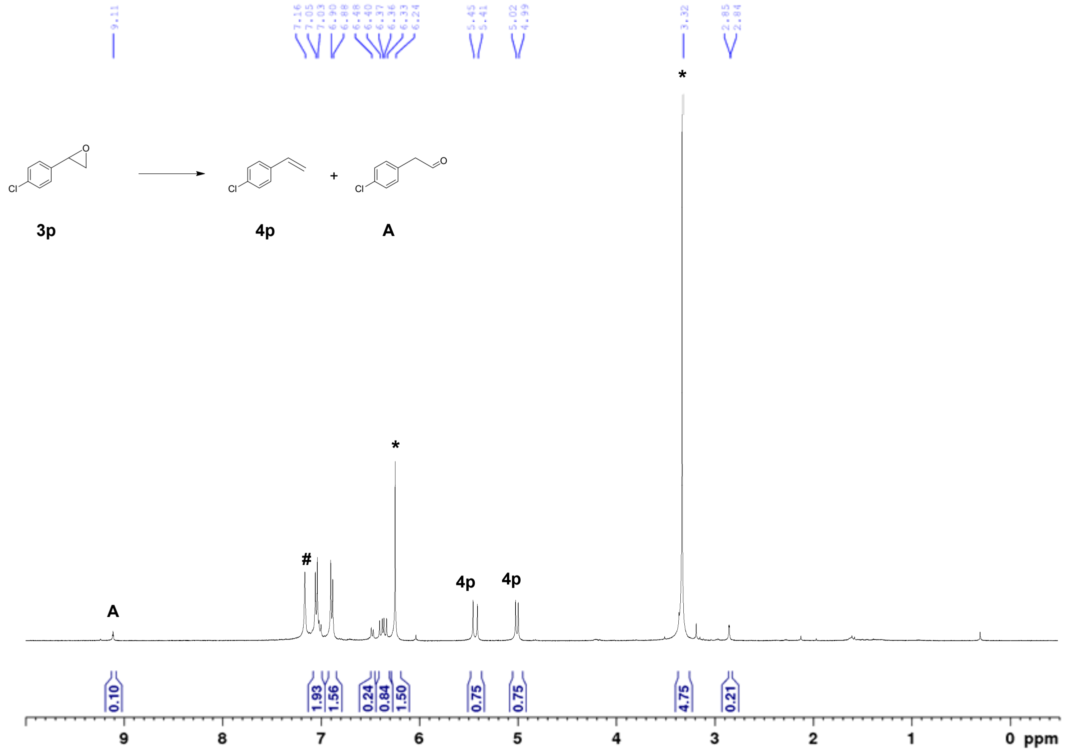

Figure S63. [1]*H NMR (C_6D_6 (#), 400 MHz) spectrum: deoxygenation of* ***3p*** *into* ***4p*** *at 100 °C; internal standard (*).*

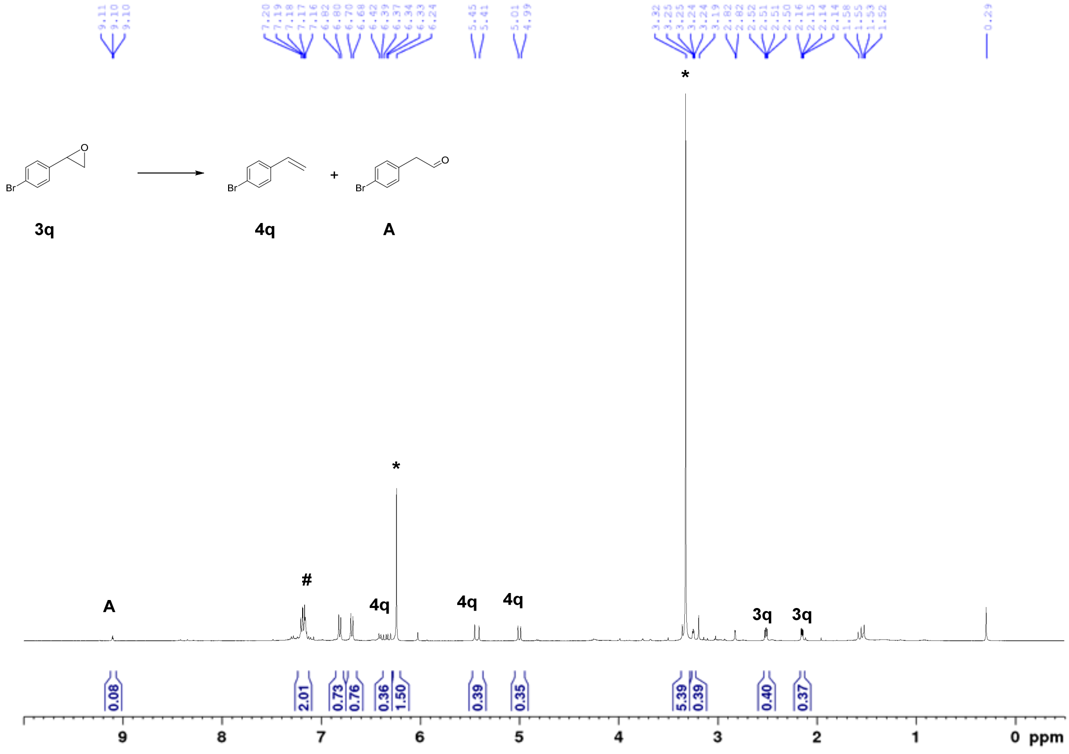

Figure S64 . [1]*H NMR (C_6D_6 (#), 400 MHz) spectrum: deoxygenation of* ***3q*** *into* ***4q****; internal standard (*).*

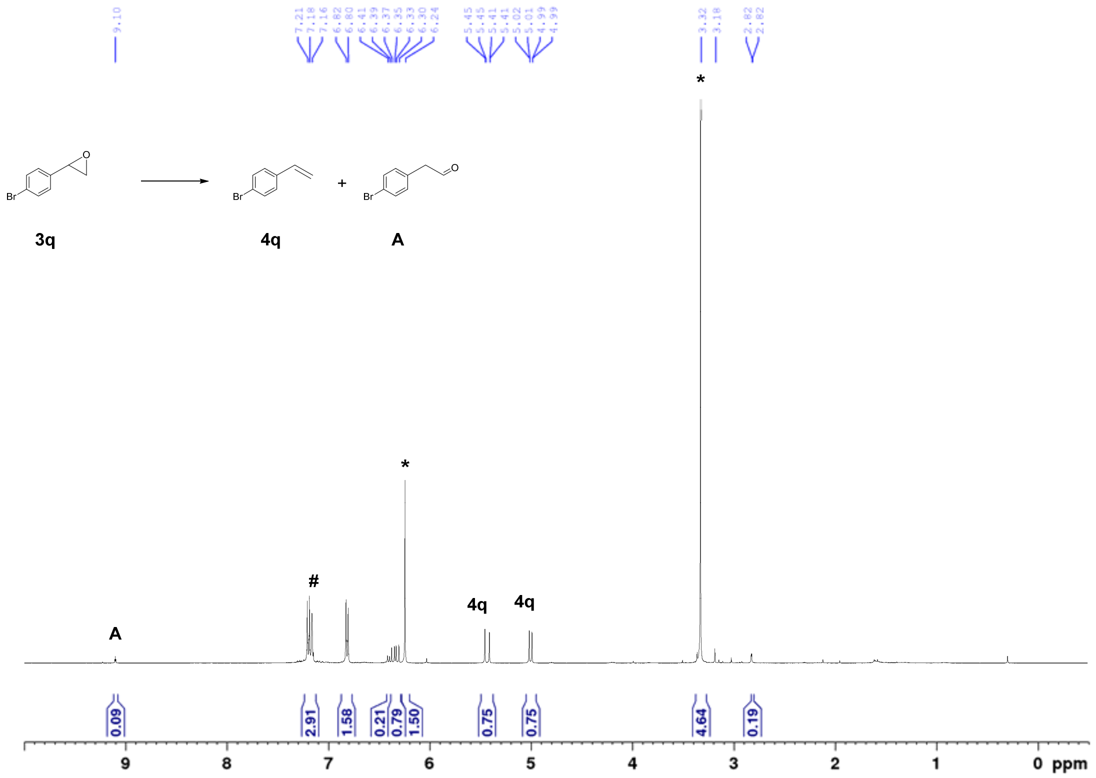

*Figure S65. ^{1}H NMR (C_6D_6 (#), 400 MHz) spectrum: deoxygenation of **3q** into **4q** at 100 °C; internal standard (*).*

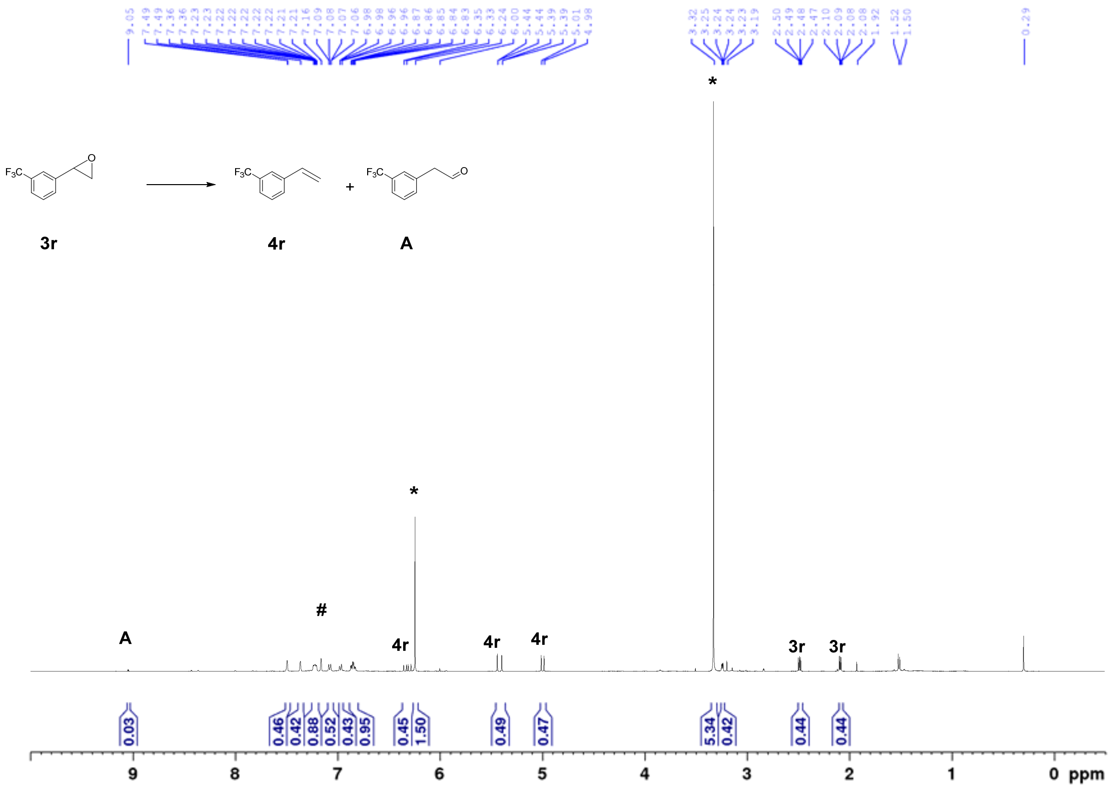

*Figure S66. ^{1}H NMR (C_6D_6 (#), 400 MHz) spectrum: deoxygenation of **3r** into **4r**; internal standard (*).*

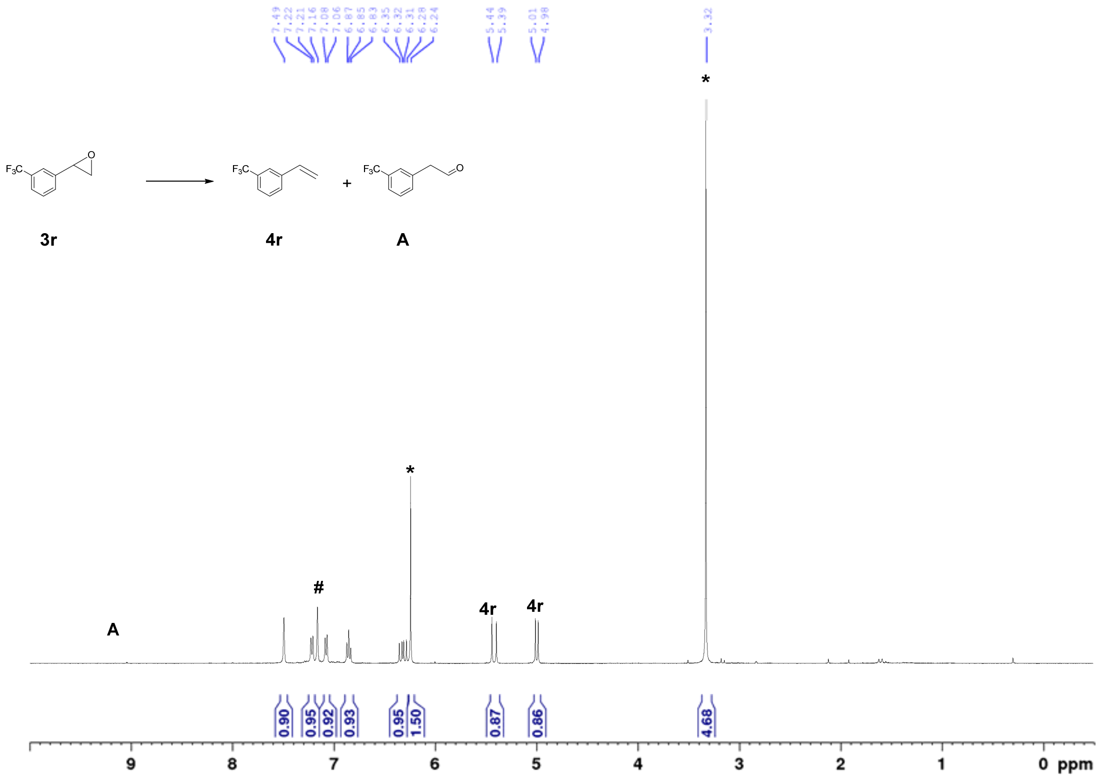

*Figure S67. ¹H NMR (C_6D_6 (#), 400 MHz) spectrum: deoxygenation of **3r** into **4r** at 100 °C; internal standard (*).*

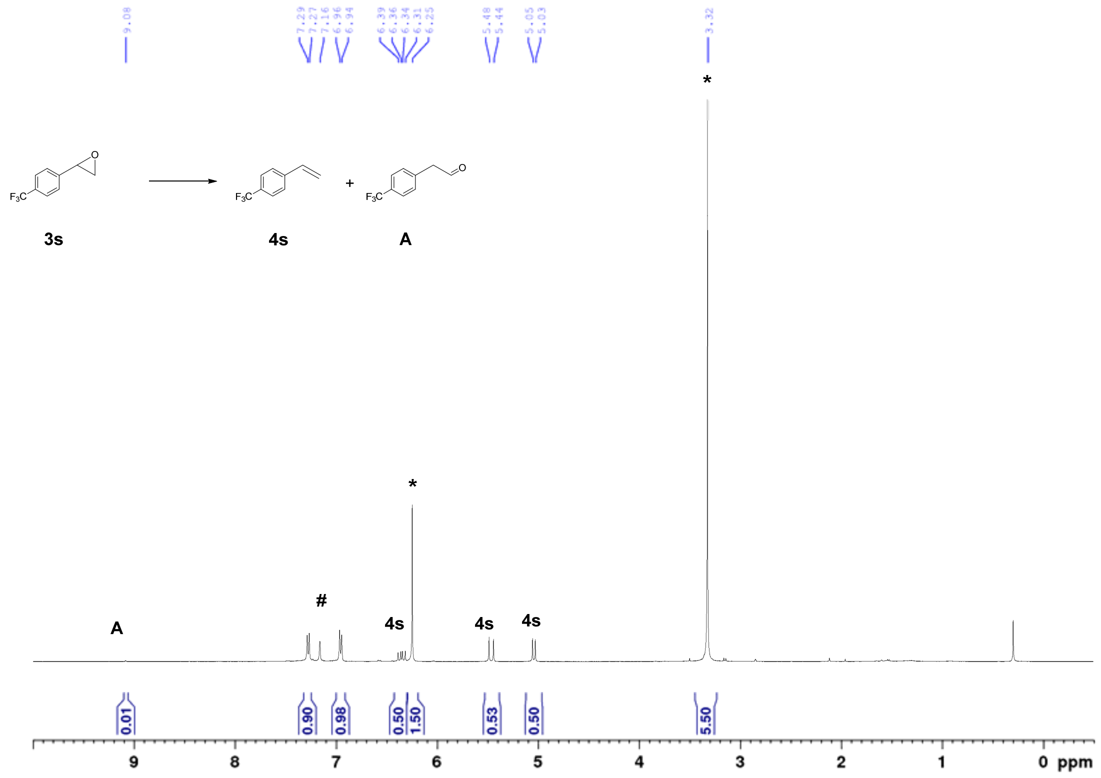

*Figure S68. ¹H NMR (C_6D_6 (#), 400 MHz) spectrum: deoxygenation of **3s** into **4s**; internal standard (*).*

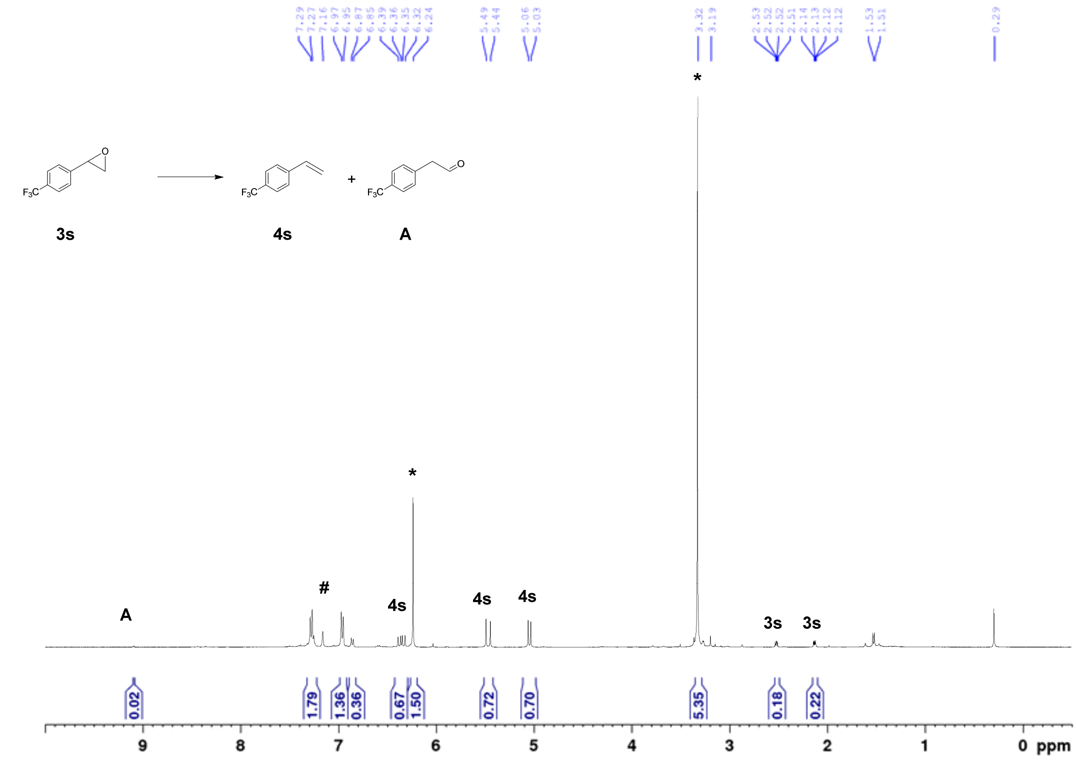

*Figure S69. ^{1}H NMR (C_6D_6 (#), 400 MHz) spectrum: deoxygenation of **3s** into **4s**; internal standard (*)*

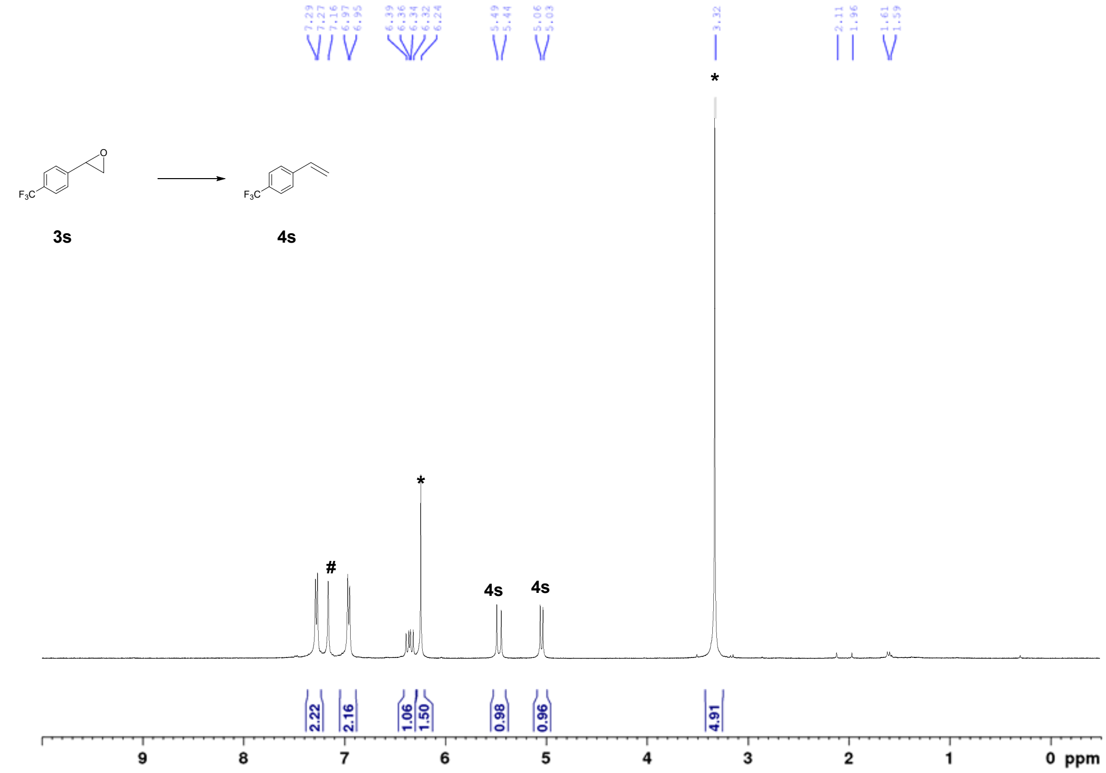

*Figure S70. ^{1}H NMR (C_6D_6 (#), 400 MHz) spectrum: deoxygenation of **3s** into **4s** at 100 °C; internal standard (*)*

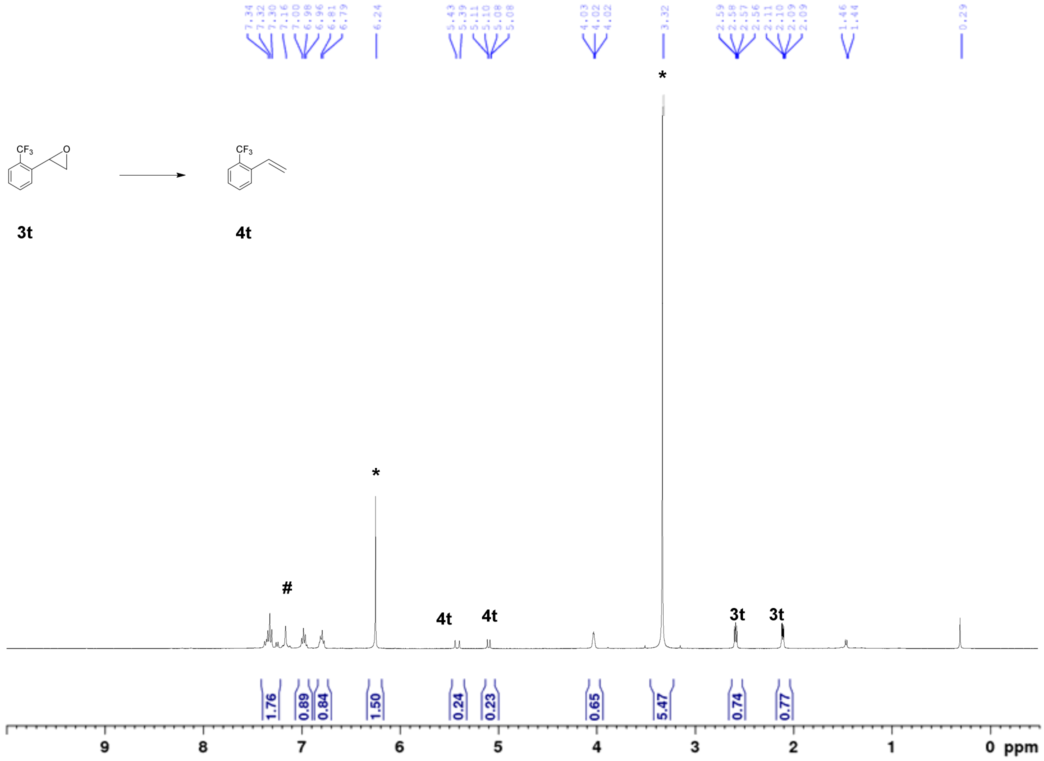

Figure S71. ¹H NMR (C_6D_6 (#), 400 MHz) spectrum: deoxygenation of **3t** into **4t**; internal standard (*).

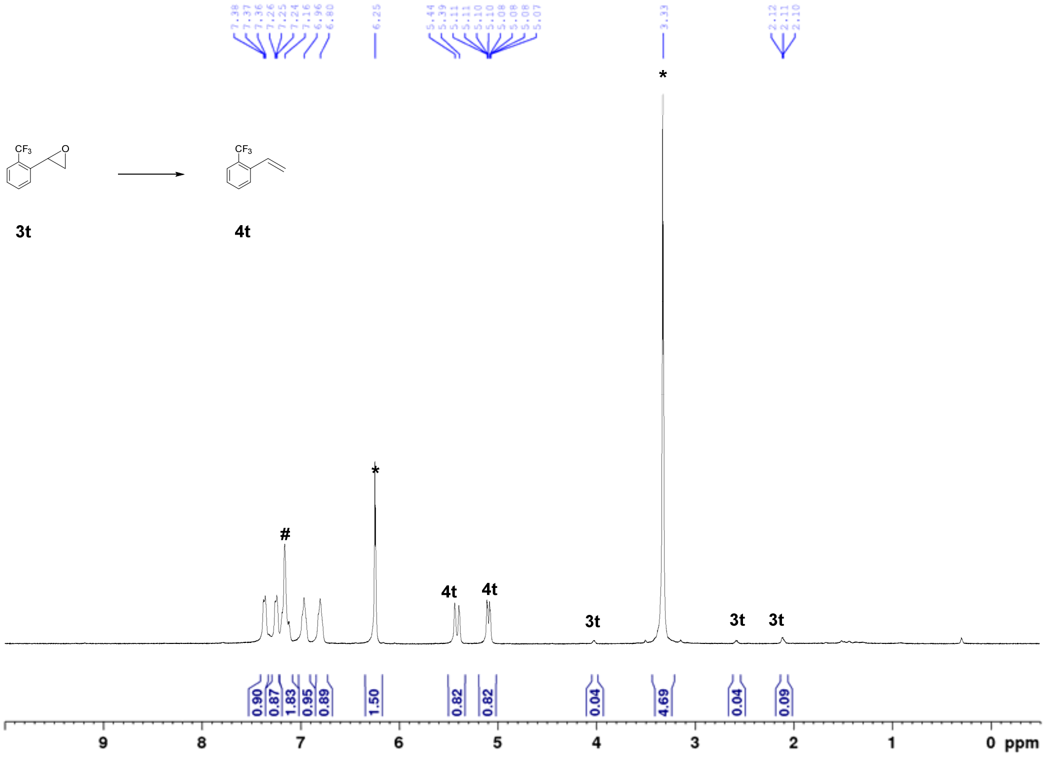

Figure S72. ¹H NMR (C_6D_6 (#), 400 MHz) spectrum: deoxygenation of **3t** into **4t** at 100 °C; internal standard (*).

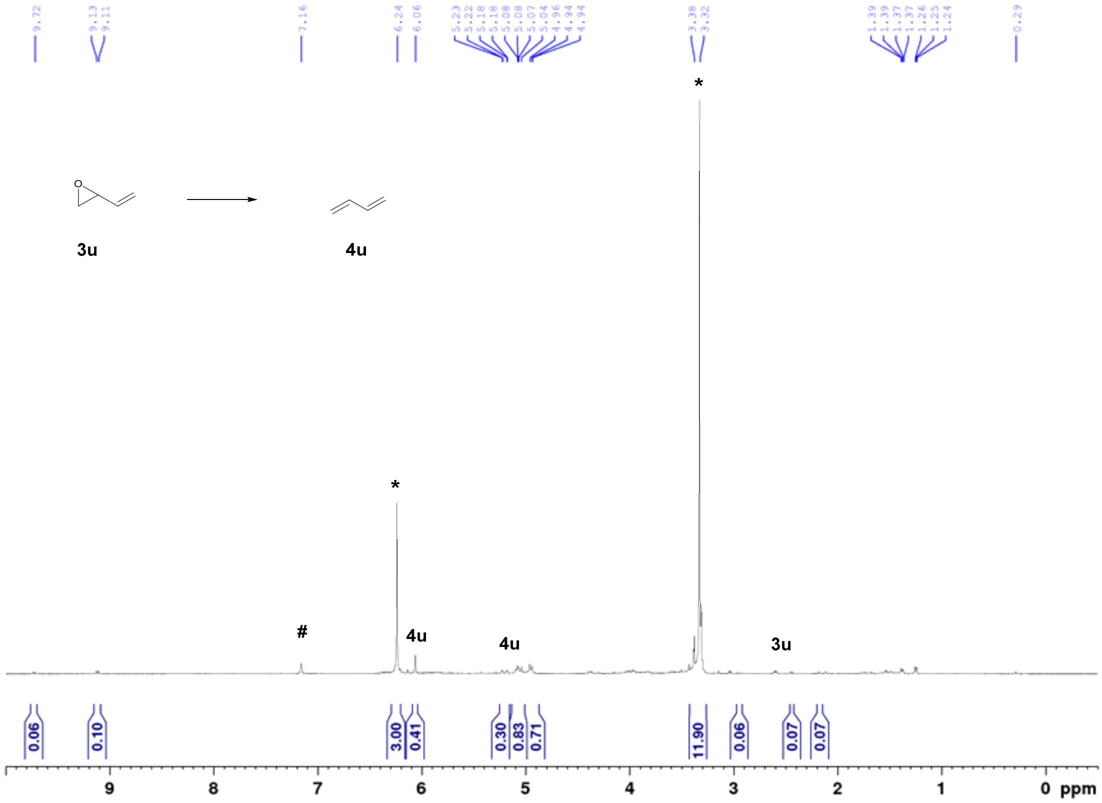

Figure S73. ^{1}H NMR (C_6D_6 (#), 400 MHz) spectrum: deoxygenation of **3u** into **4u**; internal standard (*).

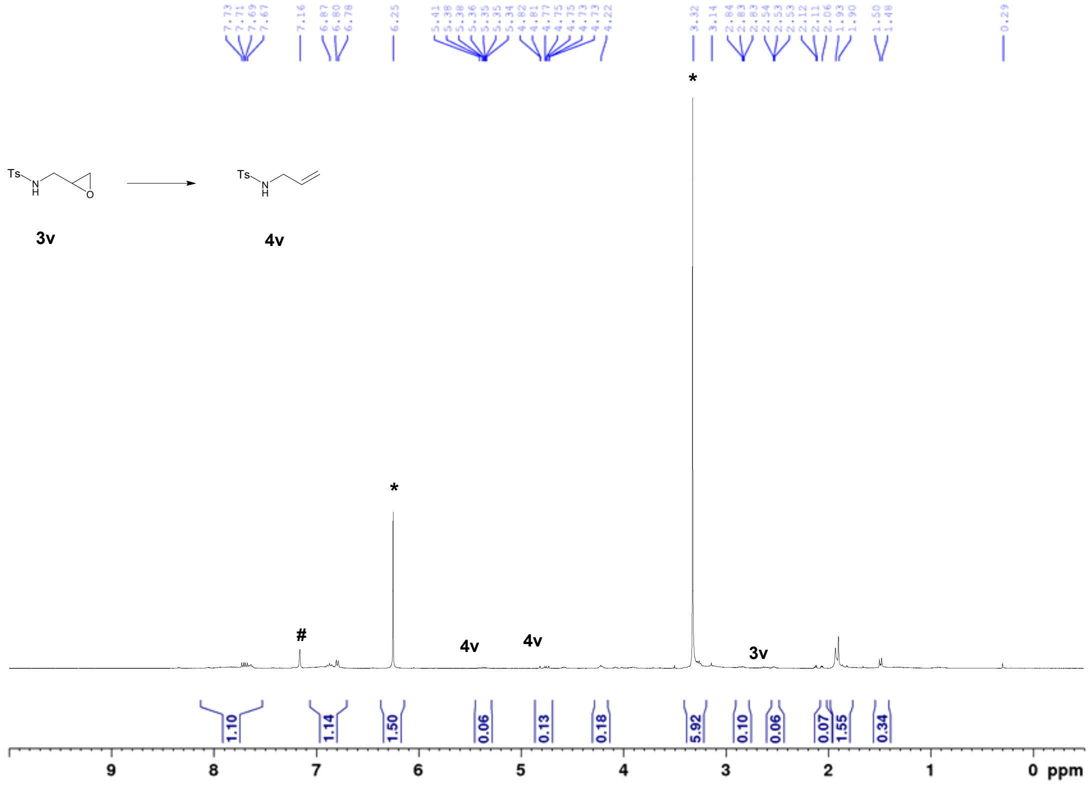

Figure S74. ^{1}H NMR (C_6D_6 (#), 400 MHz) spectrum: deoxygenation of **3v** into **4v**; internal standard (*).

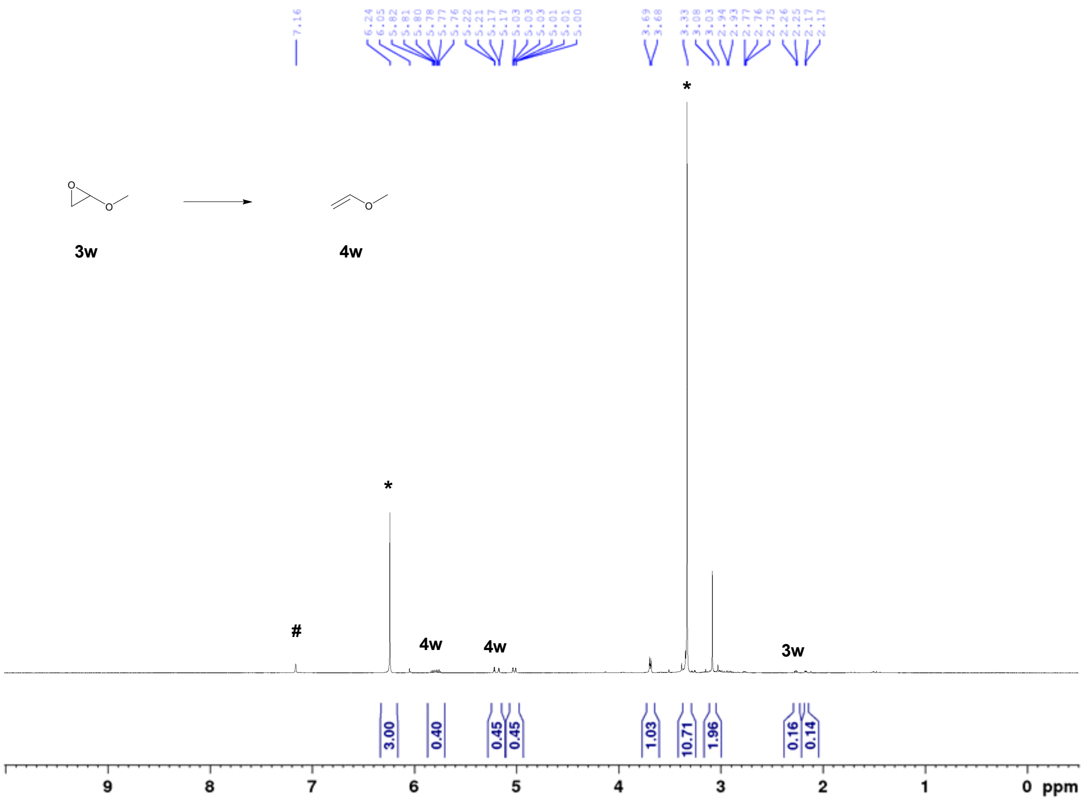

*Figure S75. ^{1}H NMR (C_6D_6 (#), 400 MHz) spectrum: deoxygenation of **3w** into **4w**; internal standard (*).*

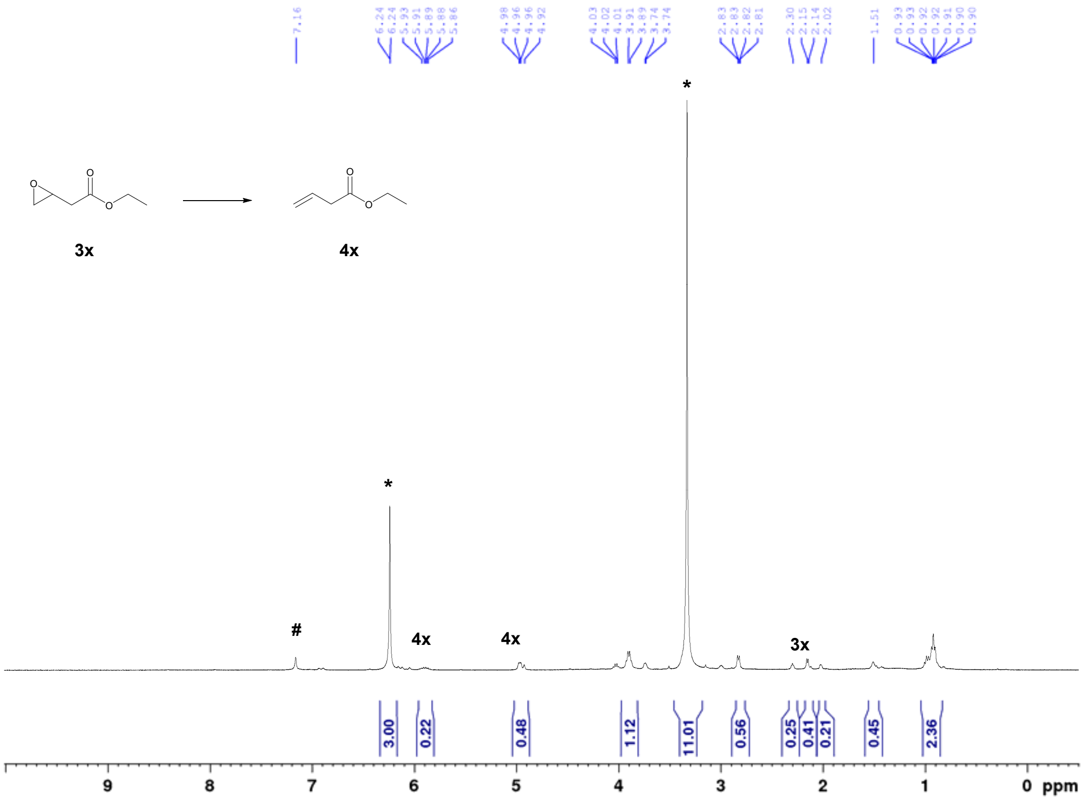

*Figure S76. ^{1}H NMR (C_6D_6 (#), 400 MHz) spectrum: deoxygenation of **3x** into **4x**; internal standard (*).*

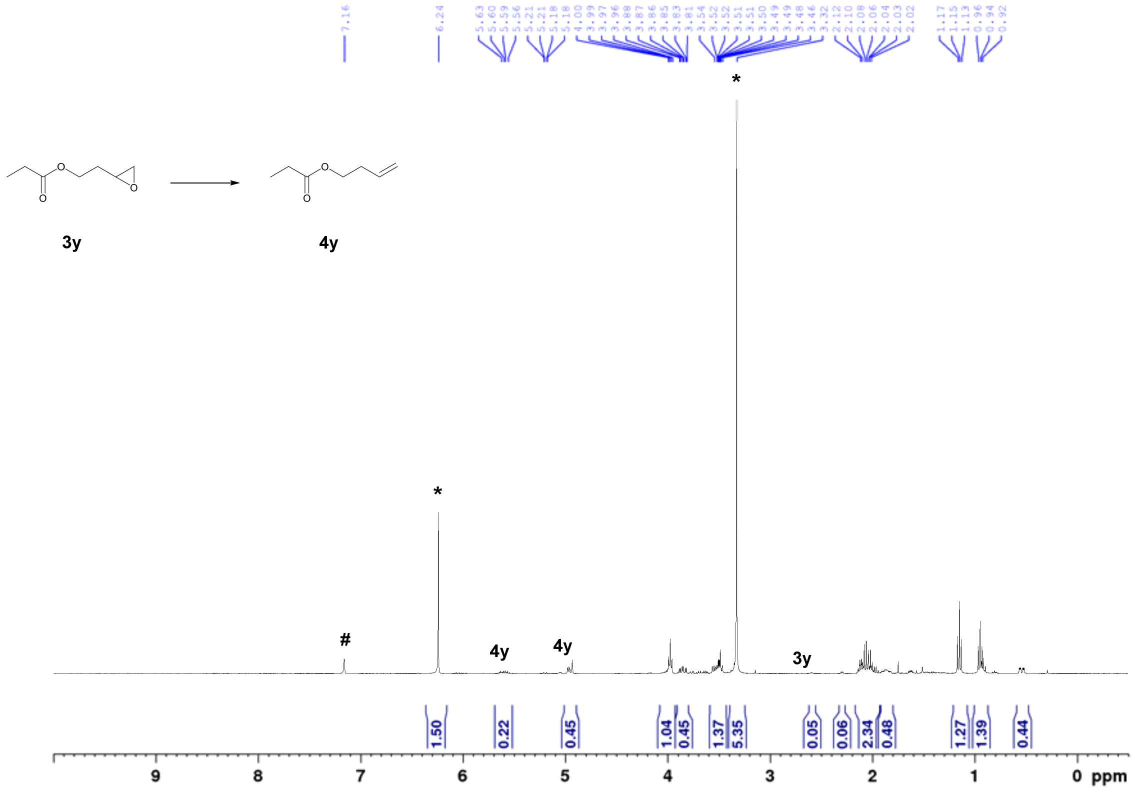

*Figure S77. ¹H NMR (C_6D_6 (#), 400 MHz) spectrum: deoxygenation of **3y** into **4y**; internal standard (*).*

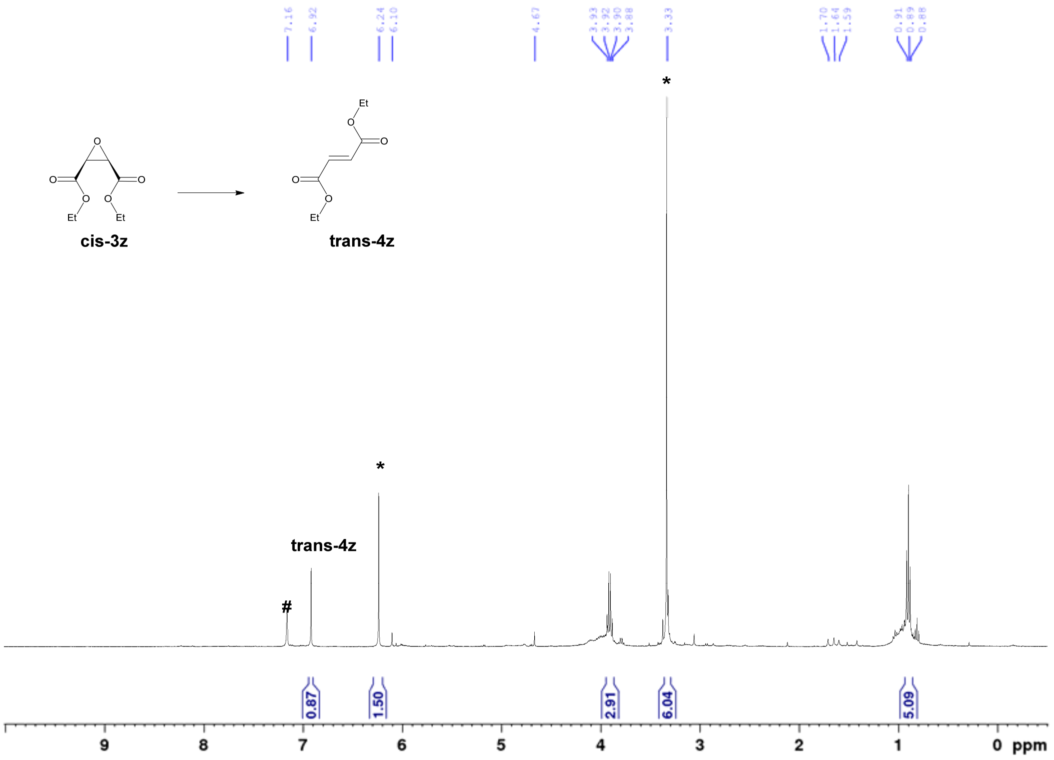

Figure S78. ^{1}H *NMR (C_6D_6 (#), 400 MHz) spectrum: deoxygenation of* ***cis-3z*** *into* ***trans-4z*** *after 240 h; internal standard (*).*

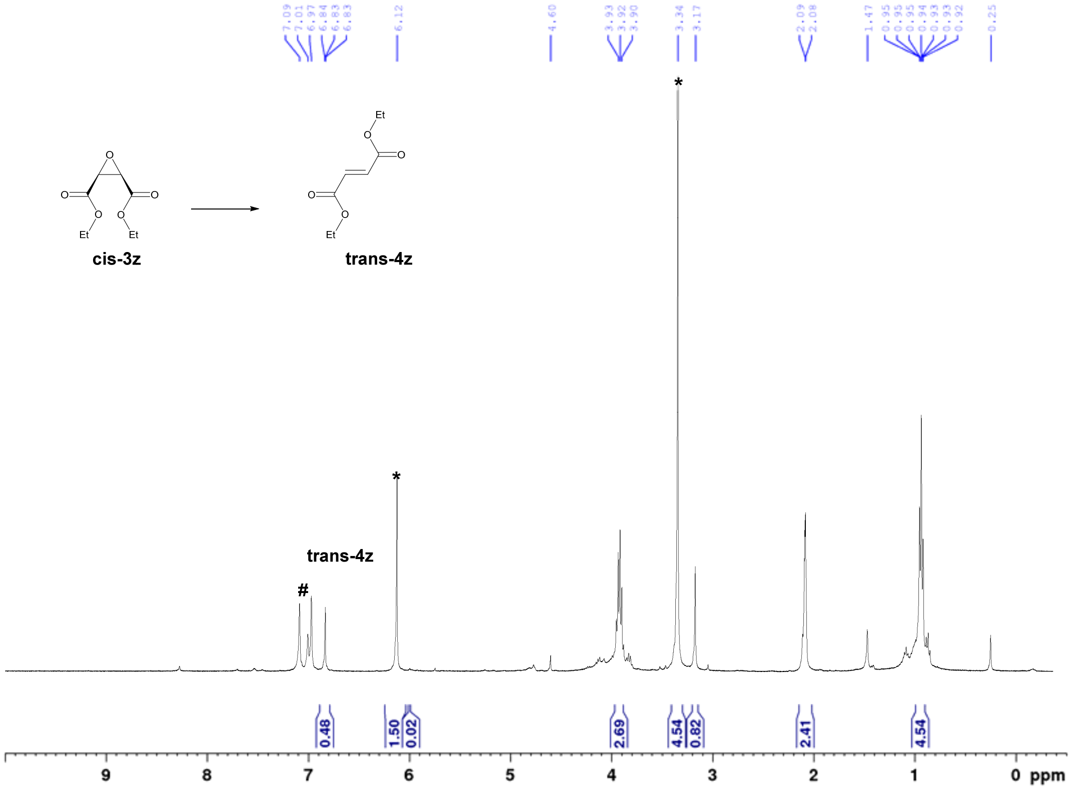

Figure S79. ^{1}H *NMR (toluene-d_8 (#), 400 MHz) spectrum: deoxygenation of* ***cis-3z*** *into* ***trans-4z*** *at 120 °C after 24 h; internal standard (*).*

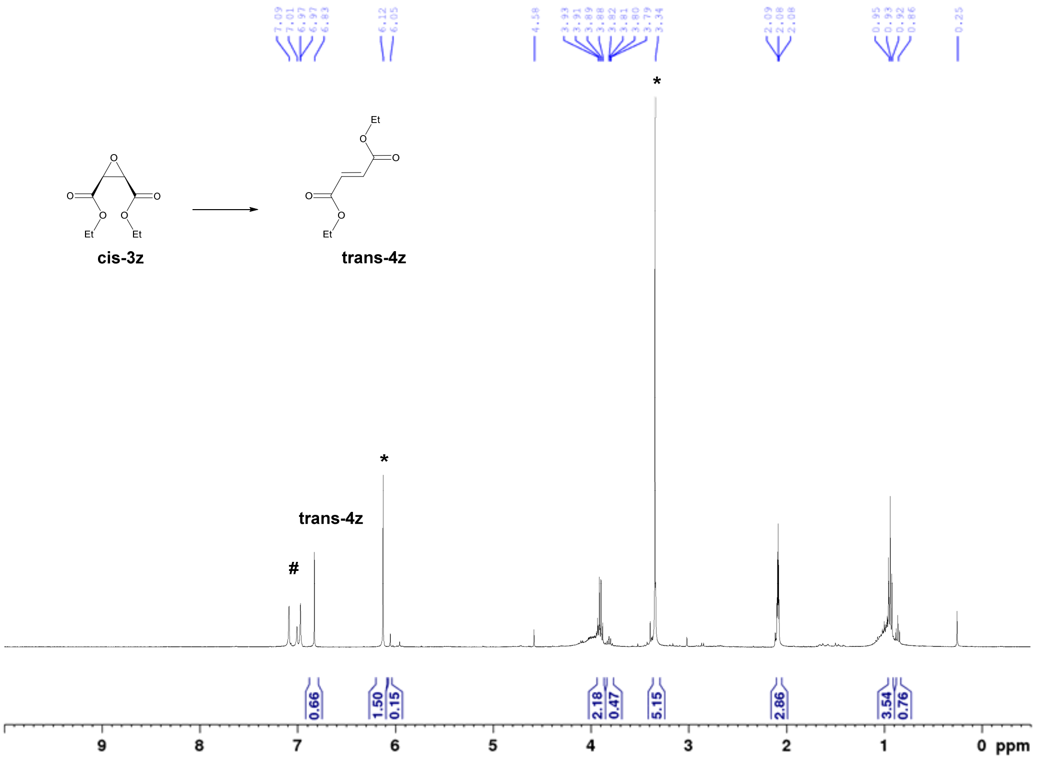

*Figure S80. ¹H NMR (toluene-d₈ (#), 400 MHz) spectrum: deoxygenation of **cis-3z** into **trans-4z** at 120 °C after 48 h; internal standard (*).*

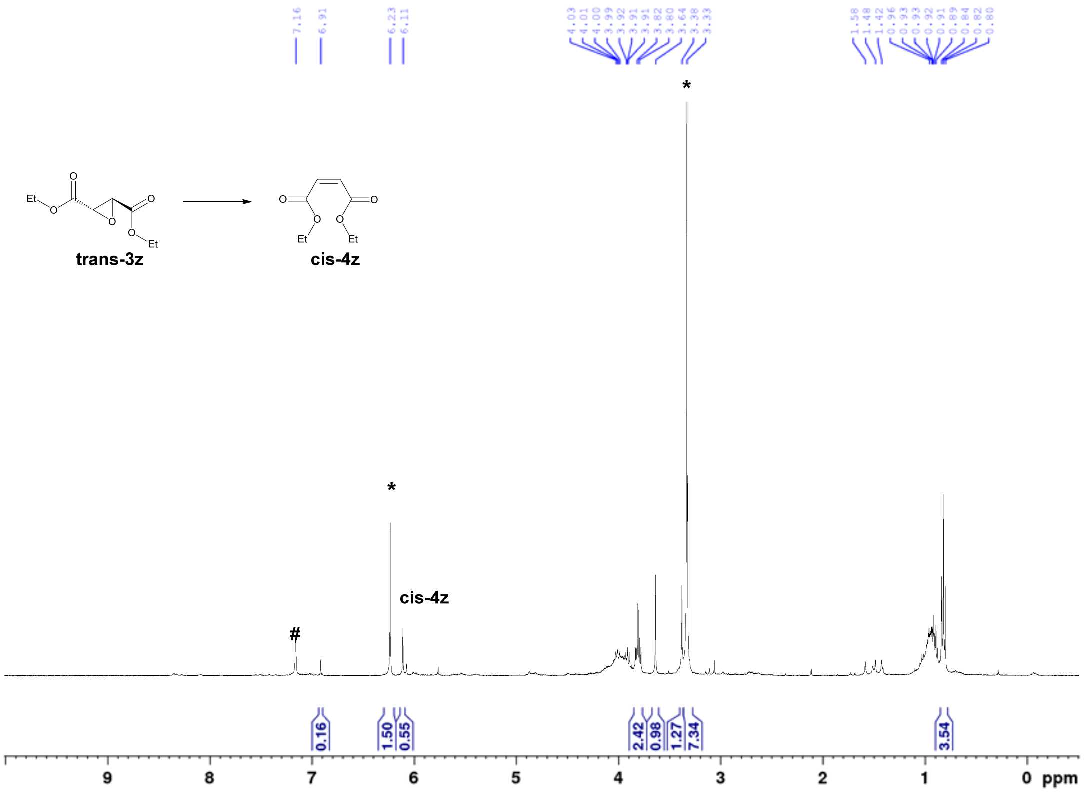

*Figure S81. ¹H NMR (C_6D_6 (#), 400 MHz) spectrum: deoxygenation of **trans-3z** into **cis-4z** after 240 h; internal standard (*).*

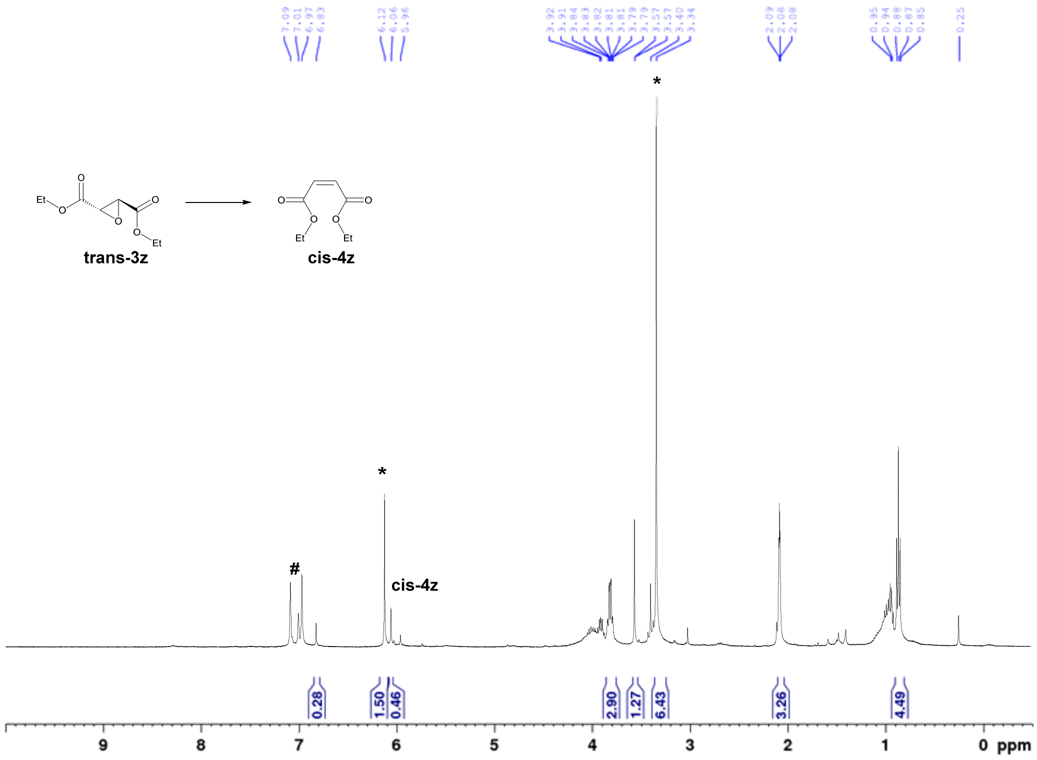

Figure S82. ^{1}H *NMR (toluene-*d_8 *(#), 400 MHz) spectrum: deoxygenation of* ***trans-3z*** *into* ***cis-4z*** *at 120 °C after 24 h; internal standard (*).*

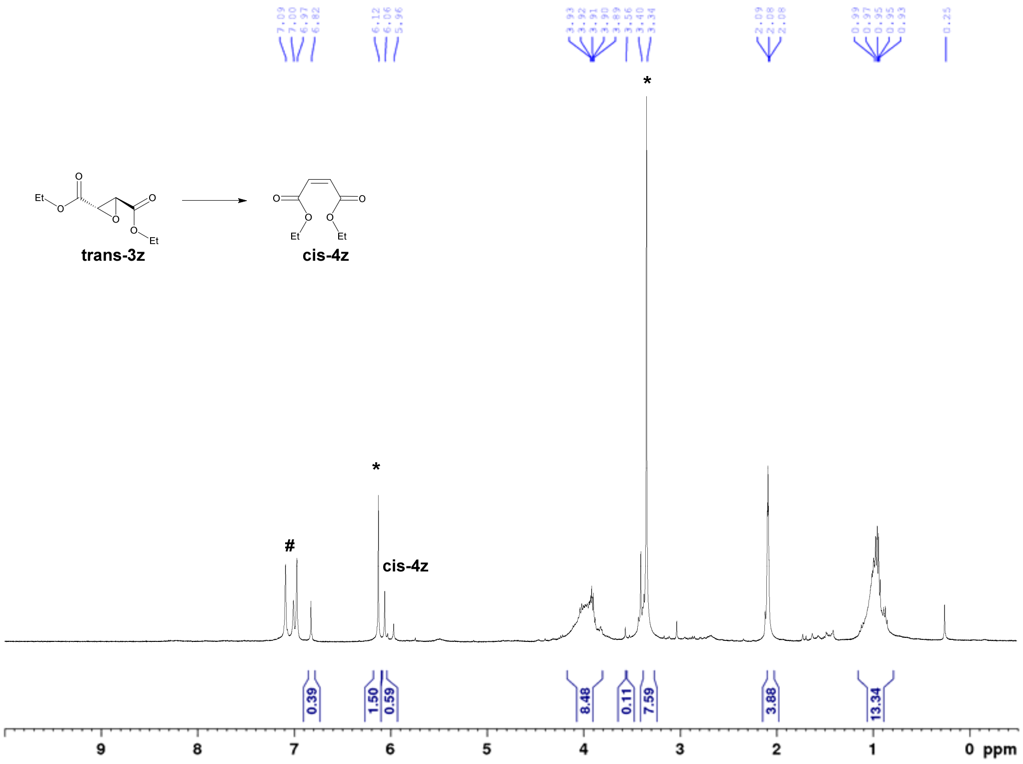

Figure S83. ^{1}H *NMR (toluene-*d_8 *(#), 400 MHz) spectrum: deoxygenation of* ***trans-3z*** *into* ***cis-4z*** *at 120 °C after 48 h; internal standard (*)*

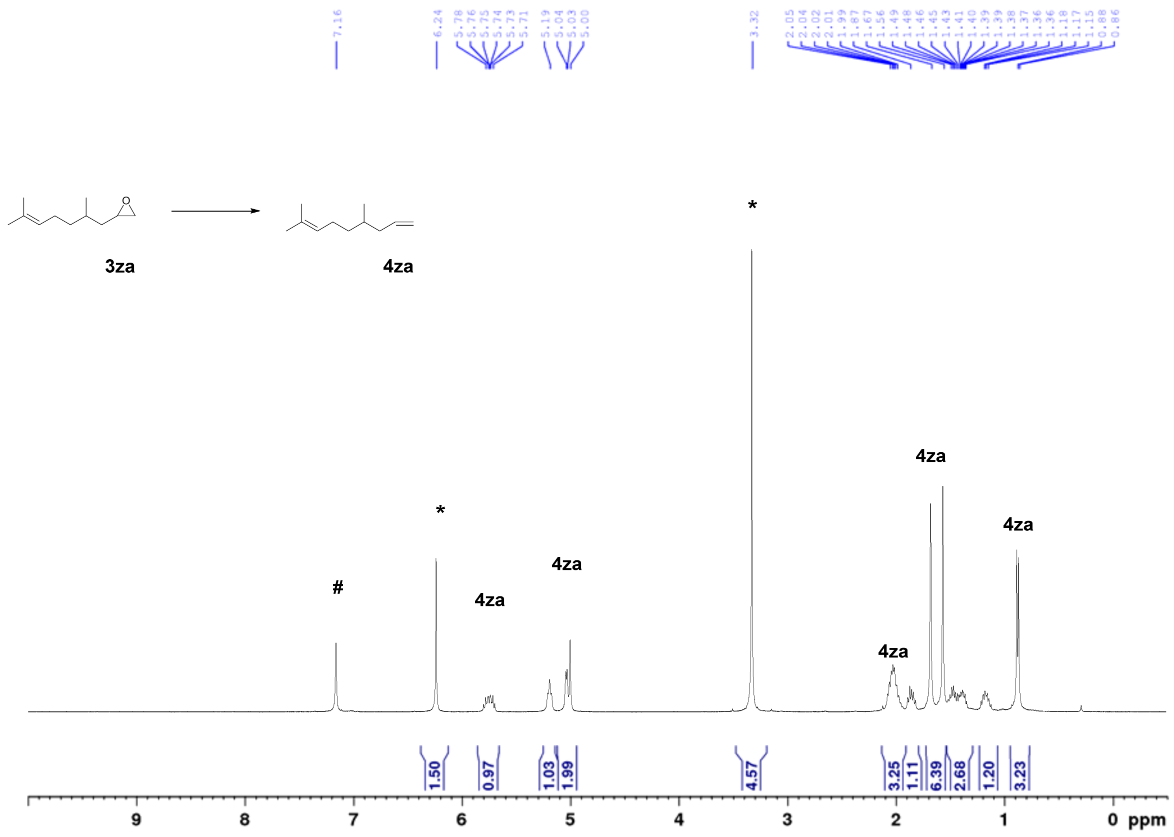

Figure S84. ¹H NMR (C_6D_6 (#), 400 MHz) spectrum: deoxygenation of **3za** into **4za**; internal standard (*).

Isolated products

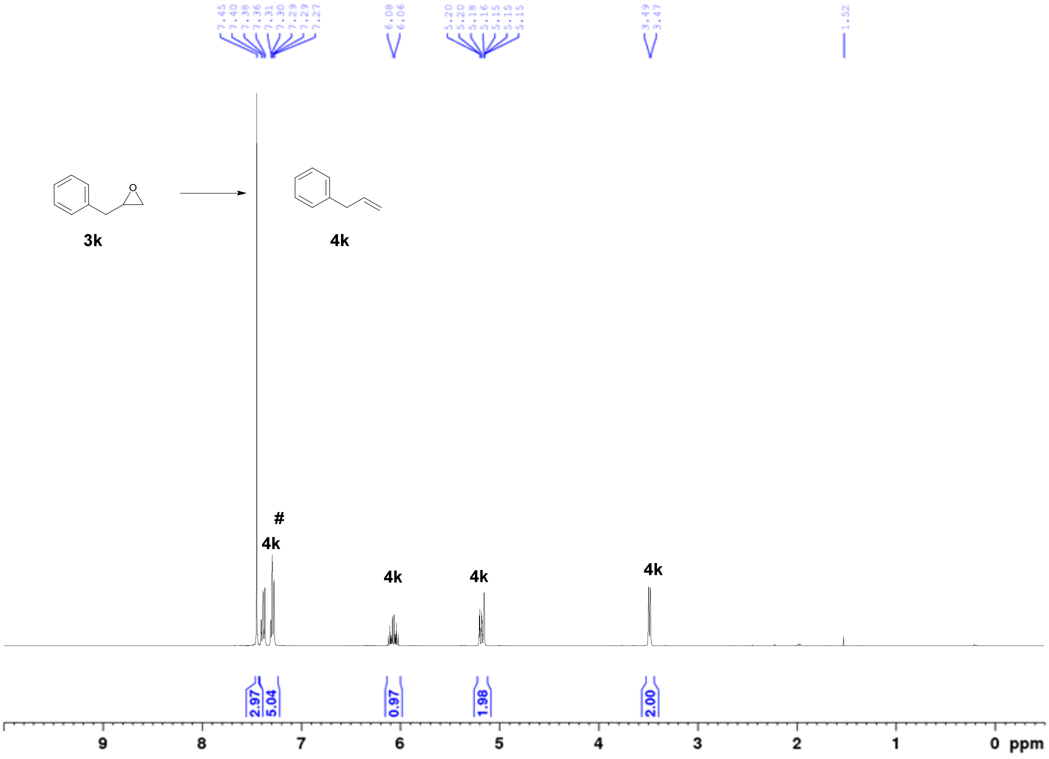

Figure S85. ^{1}H *NMR (C_6D_6 (#), 400 MHz) spectrum: deoxygenation of* ***3k*** *and isolation of* ***4k****.*

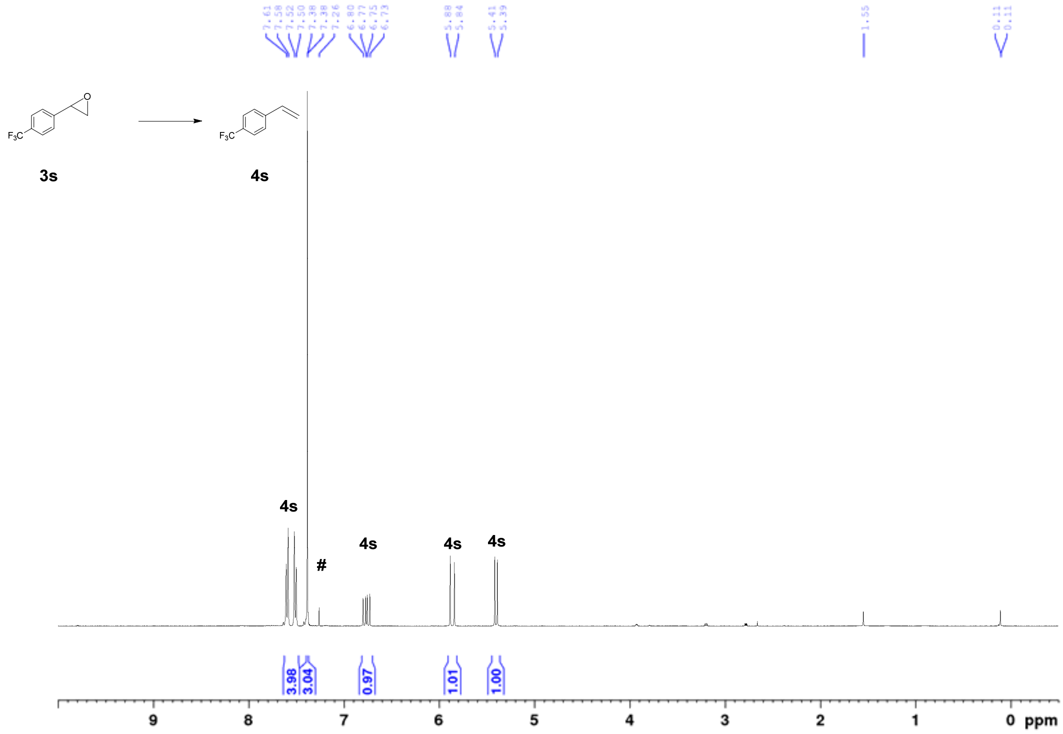

Figure S86. ^{1}H *NMR (C_6D_6 (#), 400 MHz) spectrum: deoxygenation of* ***3s*** *with isolation of* ***4s****.*

3.3 Isomerization of 1-Hexene

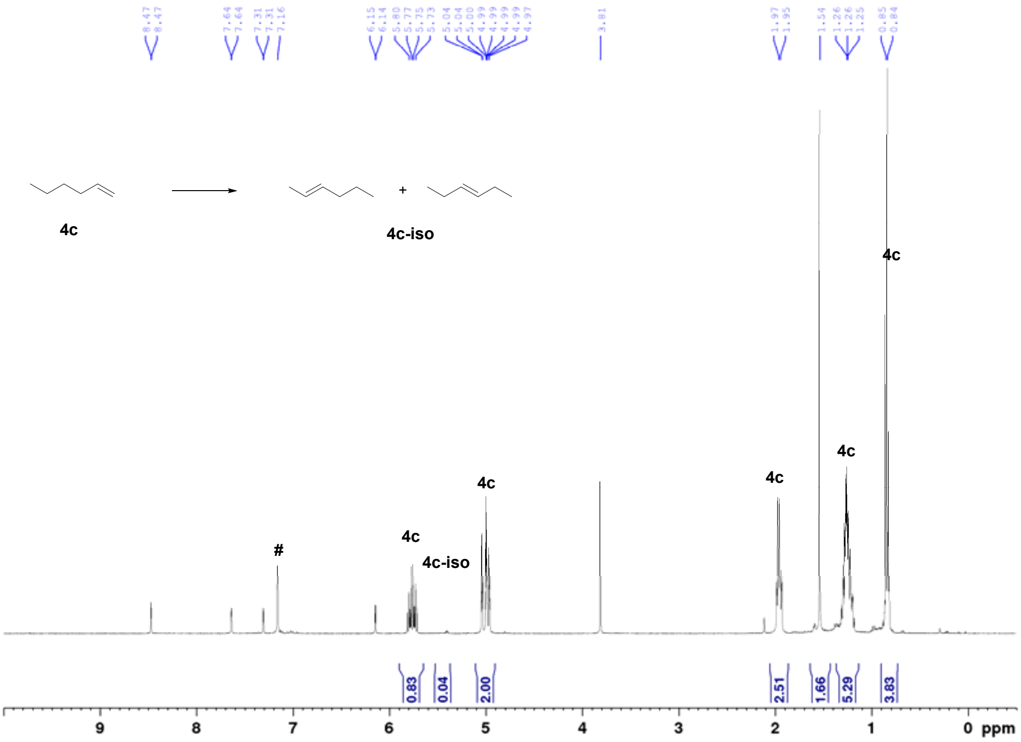

Figure S87. *¹H NMR (C_6D_6 (#), 400 MHz) spectrum: isomerization of* ***4c*** *with* ***2*** *for 7 d at 80 °C.*

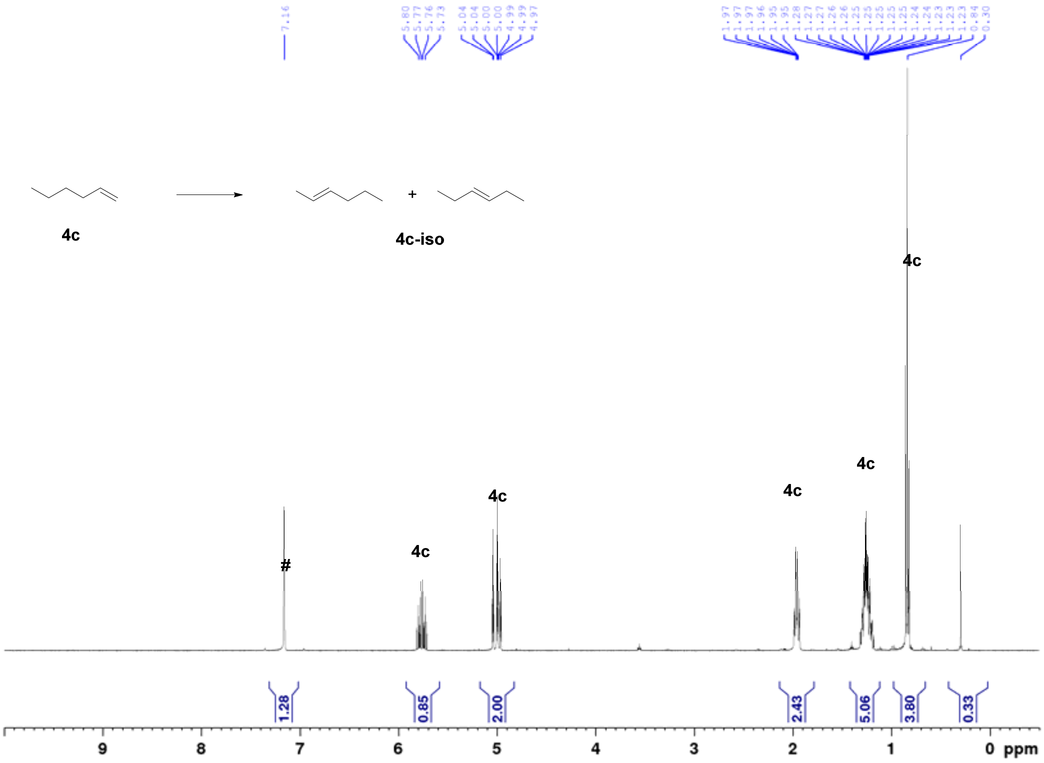

Figure S88. *¹H NMR (C_6D_6 (#), 400 MHz) spectrum: isomerization of* ***4c*** *with $LiNTf_2$ for 7 d at 80 °C.*

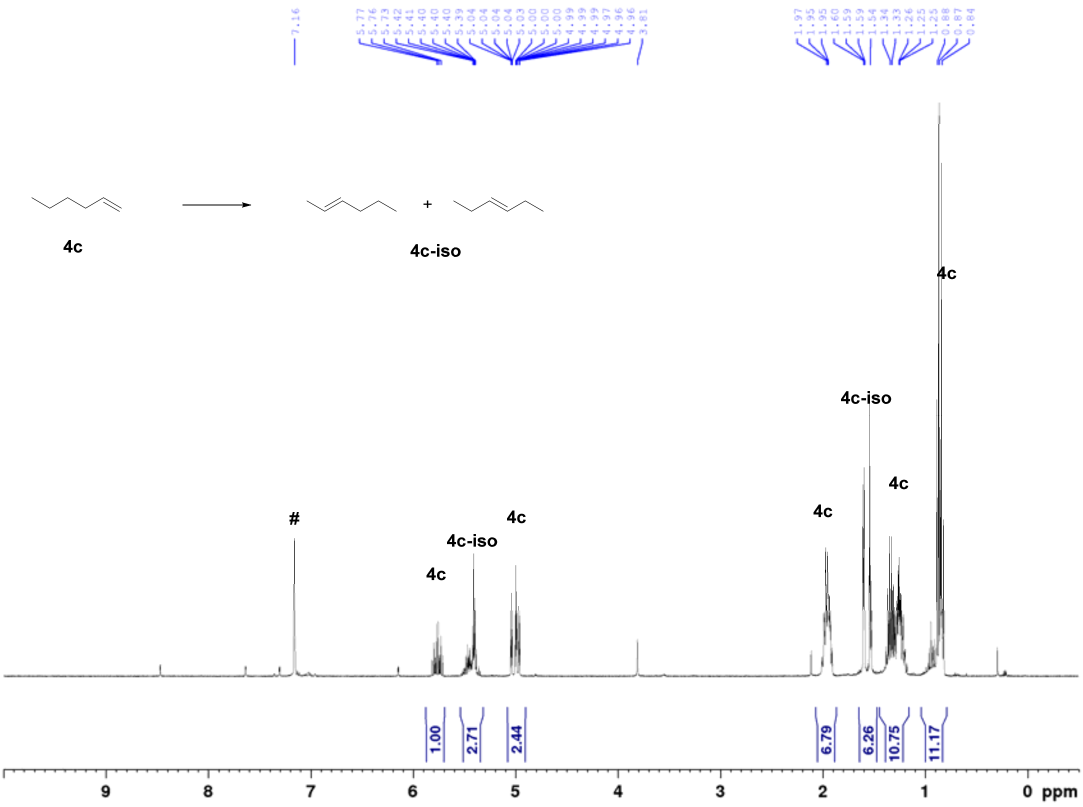

*Figure S89. ^{1}H NMR (C_6D_6 (#), 400 MHz) spectrum: isomerization of **4c** with **2** and $LiNTf_2$ for 7 d at 80 °C.*

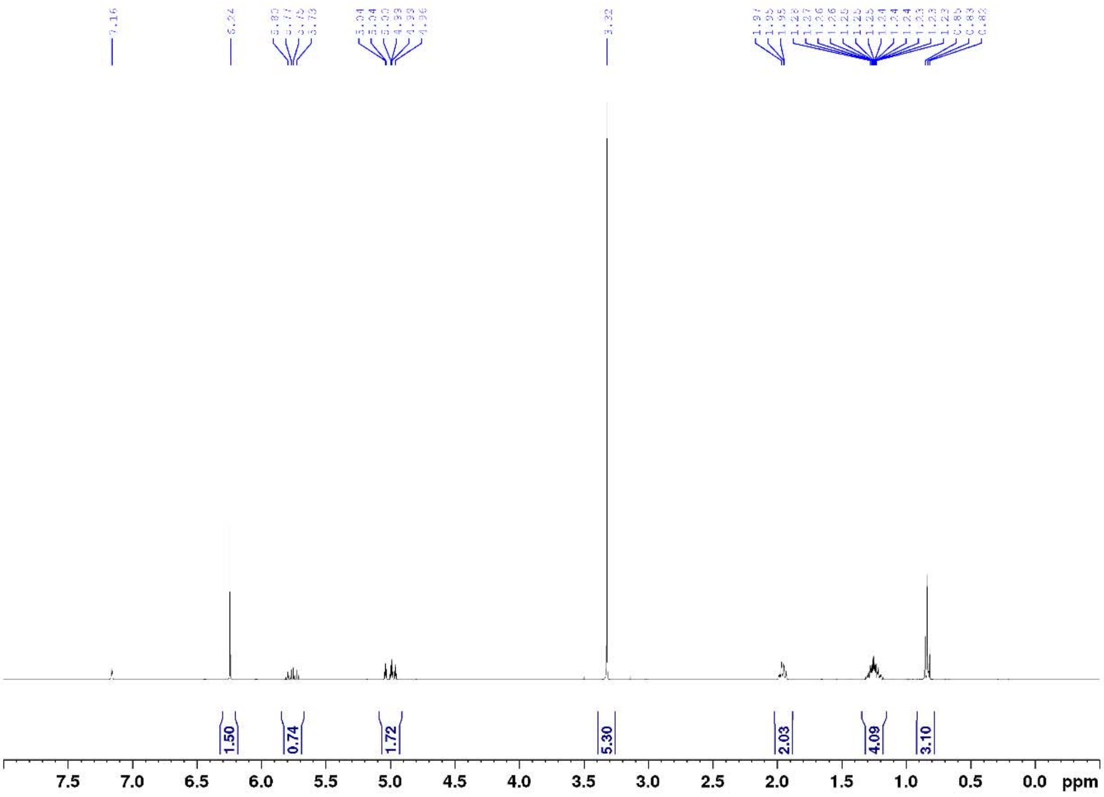

Figure S90. ^1H NMR (C_6D_6, 400 MHz) spectrum: 1-hexene with an internal standard, d_1= 1s.

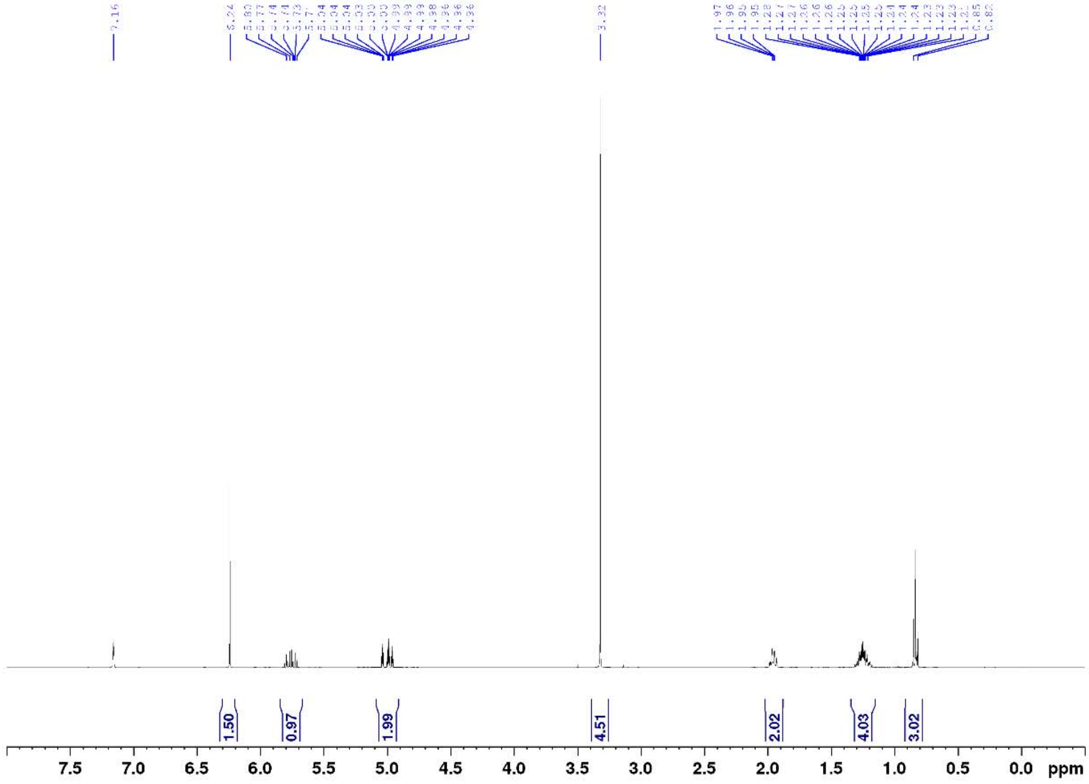

Figure S91. ^1H NMR (C_6D_6, 400 MHz) spectrum: 1-hexene with an internal standard, d_1= 64s.

4. References

[1] Zhao, D.; Liu, X.-H.; Shi, Z.-Z.; Zhu, C.-D.; Zhao, Y.; Wang, P.; Sun, W.-Y. *Dalton Trans*. **2016**, *45*, 14184−14190.

[2] Rintjema, J.; Epping, R.; Fiorani, G.; Martín, E.; Escudero-Adán, E. C.; Kleij, A. W. *Angew. Chem. Int. Ed*. **2016**, *55*, 3972−3976.

[3] Cook, C.; Liron, F.; Guinchard, X.; Roulland, E. *J. Org. Chem*. **2012**, *77*, 6728−6742.

[4] Schmidt, J. A. R.; Lobkovsky, E. B.; Coates, G. W. *J. Am. Chem. Soc*. **2005**, *127*, 11426−11435.

[5] Payne, G. B.; Williams, P. H. *J. Org. Chem* **1959**, *24*, 54–55.

[6] Sheldrick, G. M. SADABS 2012/1; University of Göttingen, Göttingen, Germany, **2012**.

[7] a) Sheldrick, G. M. *Acta Cryst.* **2008**, *A64*, 112−122; b) Sheldrick, G. M. *Acta Cryst.* **2015**, *C71*, 3−8; c) Hübschle, C. B.; Sheldrick, G. M.; Dittrich, B. *J. Appl. Cryst.* **2011**, *44*, 1281−1284.
